# Molecularly Engineered Amphiphilic Anions Enable Flame-Retarding Fluorous Electrolytes for Lithium Metal Batteries

**DOI:** 10.1021/acscentsci.5c01711

**Published:** 2025-12-25

**Authors:** Li Chen, Jiajia Fan, Xuan Luo, Hehe Zhang, Digen Ruan, Yuxuan Li, Shunqiang Chen, Lijiang Tan, Qingshun Nian, Bingqing Xiong, Zihong Wang, Jun Ma, Shuping Wang, Yifeng Cheng, Qingsong Wang, Qiang Zhao, Zhuo Kang, Lianfeng Zou, Xiaodi Ren

**Affiliations:** † Hefei National Research Center for Physical Sciences at the Microscale, School of Chemistry and Materials Science, 12652 University of Science and Technology of China, Anhui 230026, China; ‡ Institutes of Physical Science and Information Technology, Anhui University, Hefei 230601, China; § Clean Nano Energy Center, State Key Laboratory of Metastable Material Science and Technology, 26472 Yanshan University, Qinhuangdao 066004, China; ∥ State Key Laboratory of Fire Science, University of Science and Technology of China, Hefei 230601, China; ⊥ State Grid Anhui Electric Power Research Institute, State Grid Laboratory of Fire Protection for Transmission and Distribution Facilities, Anhui Province Key Laboratory of Electric Fire and Safety Protection, Hefei 230601, China; # School of Chemical Engineering, Sichuan University, Chengdu 610065, China; 7 Academy for Advanced Interdisciplinary Science and Technology, Beijing Key Laboratory for Advanced Energy Materials and Technologies, State Key Laboratory for Advanced Metals and Materials and Beijing Advanced Innovation Center for Materials Genome Engineering, School of Materials Science and Engineering, Key Laboratory of Advanced Materials and Devices for Post-Moore Chips Ministry of Education, University of Science and Technology Beijing, Beijing 100083, China

## Abstract

Developing high-energy-density lithium metal batteries (LMBs) is challenging due to critical safety concerns and cycling instability. A highly fluorinated diluent offers improved safety features but fails to form miscible electrolytes. Herein, we address these key issues through the design of miscible fluorous electrolytes enabled by molecular engineering of anions with fluoro-alkyl moieties, creating an effective molecular bridge between solvents and fluorous diluents. Detailed spectroscopy and molecular dynamics simulations reveal the critical amphiphilic anion chemistry inward and outward of the Li^+^ solvation sheath: fluorophilic interactions (F···F) with the diluent and atypical hydrogen-bonding (F···H) with the solvent. The designed miscible fluorous electrolyte, featuring diluents with ultrahigh F/H atomic ratios of 4.33 or higher, exhibits not only remarkable nonflammability safety properties, but also dendrite-free Li plating/stripping with a high Coulombic efficiency (CE) of 99.53% and long-term cycling stability in Li||NCM811 batteries. LiF-rich interphases formed at the electrode–electrolyte interface and the unique electrolyte formulation greatly enhance the battery performance and safety profile, as characterized by delayed onset and peak temperatures of thermal runaway reactions. This study demonstrates a general approach for engineering high-safety electrolytes, advancing next-generation LMBs that overcome the traditional trade-off between performance and safety.

## Graphical abstract

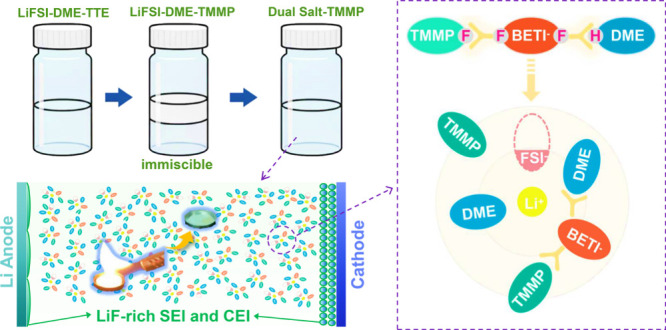

## Introduction

Lithium metal batteries (LMBs) with high voltage cathodes are attracting intensive attention from academic and industrial researchers because of their superior energy densities (>500 Wh kg^–1^) compared to lithium-ion batteries.^1–5^ Li metal, considered a promising anode, has an exceptional theoretical specific capacity (3860 mAh g^–1^) and a low electrochemical redox potential (−3.04 V vs. the standard hydrogen electrode).^6–9^ Nevertheless, LMBs encounter major obstacles in commercialization due to safety and stability limitations.^10–14^ The inherent reactivity of lithium metal triggers side reactions with the electrolyte, resulting in active lithium and electrolyte consumption, dendrite growth, and battery self-heating. These issues compromise battery performance and simultaneously raise serious safety concerns. Under extreme conditions, including overcharging, short-circuiting, or high thermal impact, the highly flammable electrolytes can ignite, potentially leading to catastrophic failure.^15,16^ Ni-rich layered cathodes further heighten safety risks because their delithiated states contain highly reactive Ni^4+^ species, which can trigger severe parasitic reactions with electrolytes at high voltages and accelerate thermal runaway.^17–19^

Addressing these issues has motivated the design of innovative electrolytes to strengthen the stability of lithium metal batteries.^5,15^ High concentration electrolytes (HCEs) with reduced free solvents and reactive anions can suppress the side reactions by generating inorganic-rich solid electrolyte interphases (SEI).^20–22^ Ether-based HCEs (e.g., 4 M lithium bis­(fluorosulfonyl)­imide (LiFSI) in 1,2-dimethoxyethane (DME)) enable high Li Coulombic efficiency (99.10%) and provide stability to high-voltage (>4 V) cathodes.^23,24^ Nevertheless, HCEs are constrained by high costs and increased viscosity, limiting their practical application. Moreover, the flammability of organic solvents in these systems can only be suppressed to a limited extent.^25^ To overcome these limitations, localized high concentration electrolyte (LHCE) emerged through the use of partially fluorinated ethers as diluents. Compounds such as bis­(2,2,2-trifluoroethyl) ether (BTFE) and 1,1,2,2-tetrafluoroethyl-2,2,3,3-tetrafluoropropyl ether (TTE) have been successfully employed in LHCEs.^26–28^ Fluorine atoms in these partially fluorinated ethers exert an electron-withdrawing influence, which reduces their ability to solvate Li^+^ ions and preserves the favorable solvation complexes and electrochemical properties of HCEs.^29,30^

Despite significant progress in electrolyte design, the current options for LHCE diluents are limited to weakly polar or fluorinated solvents with relatively low degrees of fluorination, which presents a significant challenge in further enhancing electrolyte safety. The safety concern stems from the general inverse relationship between the degree of fluorination and the flash point of these diluents. The commonly reported fluorinated diluents with low flash points (the lowest ignition temperature after liquid vaporization) are still prone to ignition due to their low F/H ratios (BTFE, F/H ratio = 1.5, flash point = 1 °C; TTE, F/H ratio = 2, flash point = 27.5 °C). Conversely, fluorous diluents with higher F/H ratios offer improved flame retardancy. However, these compounds tend to induce phase separation in electrolytes. This phase separation is primarily due to the increased solvent-phobicity and reduced polarity of highly fluorinated molecules, which makes them immiscible with the more polar components of the electrolyte.^31–33^

To address this critical issue, we delve into the immiscibility issue of fluorous diluents and propose a novel amphiphilic anion chemistry to bridge the solvent and fluorous diluents. Fluorinated anions have long been recognized for their ability to stabilize interfaces and improve ionic transport. In particular, Passerini and co-workers demonstrated that the BETI^–^ anion [bis­(perfluoroethylsulfonyl)­imide, N­(SO_2_CF_2_CF_3_)_2_^–^] provides excellent interfacial stability with lithium metal and high ionic conductivity in polymer electrolytes.^34,35^ By introducing anions with long fluoro-alkyl moieties (e.g., BETI^–^), we induce favorable fluorophilic (F···F) interactions with fluorous diluents (e.g., 2-trifluoromethyl-3-methoxyperfluoropentane, TMMP, F/H = 4.33; 1H-perfluorohexane, F/H = 13) and atypical hydrogen-bonding (F···H) interactions with the solvent (DME), successfully resolving the immiscibility issue. Moreover, the designed LiBETI-LiFSI-DME-TMMP electrolyte (1:0.25:2:2 by mol) (denoted as Dual Salt-TMMP) achieves a high Li CE of approximately 99.53%. Li||NCM811 cells exhibit stable cycling with ∼87% capacity retention over 200 cycles at 4.4 V, facilitated by LiF-enriched anode/cathode electrolyte interphases. Moreover, the delithiated cathodes shows enhanced thermal stability in the fluorous electrolyte. This study offers a generic strategy to address the incompatibility of fluorous cosolvents in battery electrolytes and provides a promising avenue for high-safety and high stability energy storage solutions.

## Results and Discussion

### The Mechanisms Underlying Phase Separation of LHCE

The safety of LMBs using LHCE largely relies on the characteristics of diluents, a major electrolyte component. Hydrofluoroethers (HFEs) have emerged as promising diluents due to their flame-retardant characteristics. The high bond dissociation energy of C–F bonds, reaching up to 130 kcal/mol, plays a key role in enhancing their stability. Moreover, the substitution of hydrogen with fluorine not only reduces the availability of labile hydrogen but also enables the scavenging of hydrogen radicals during combustion, effectively interrupting chain reactions.^36^ Figure 1a and Table S1 present a comparative analysis of flash points and other physical properties for various HFEs from public records. The trend indicates that as the F/H molar ratio increases, so does the flash point (Figure 1a). The flash point of TMMP was determined using the Pensky-Martens closed-cup method, which minimizes electrolyte evaporation during heating.^37,38^ Notably, TMMP, with its exceptionally high F/H ratio of 4.33, exhibits no measurable flash point within the upper limit of the test temperature (200 °C). This characteristic represents a significant advancement over widely used diluents like TTE, which itself possesses a relatively low flash point of 27 °C. The superior safety performance of TMMP in this regard underscores its potential to substantially enhance the safety profile of LHCEs in advanced battery systems.

**Figure 1:**
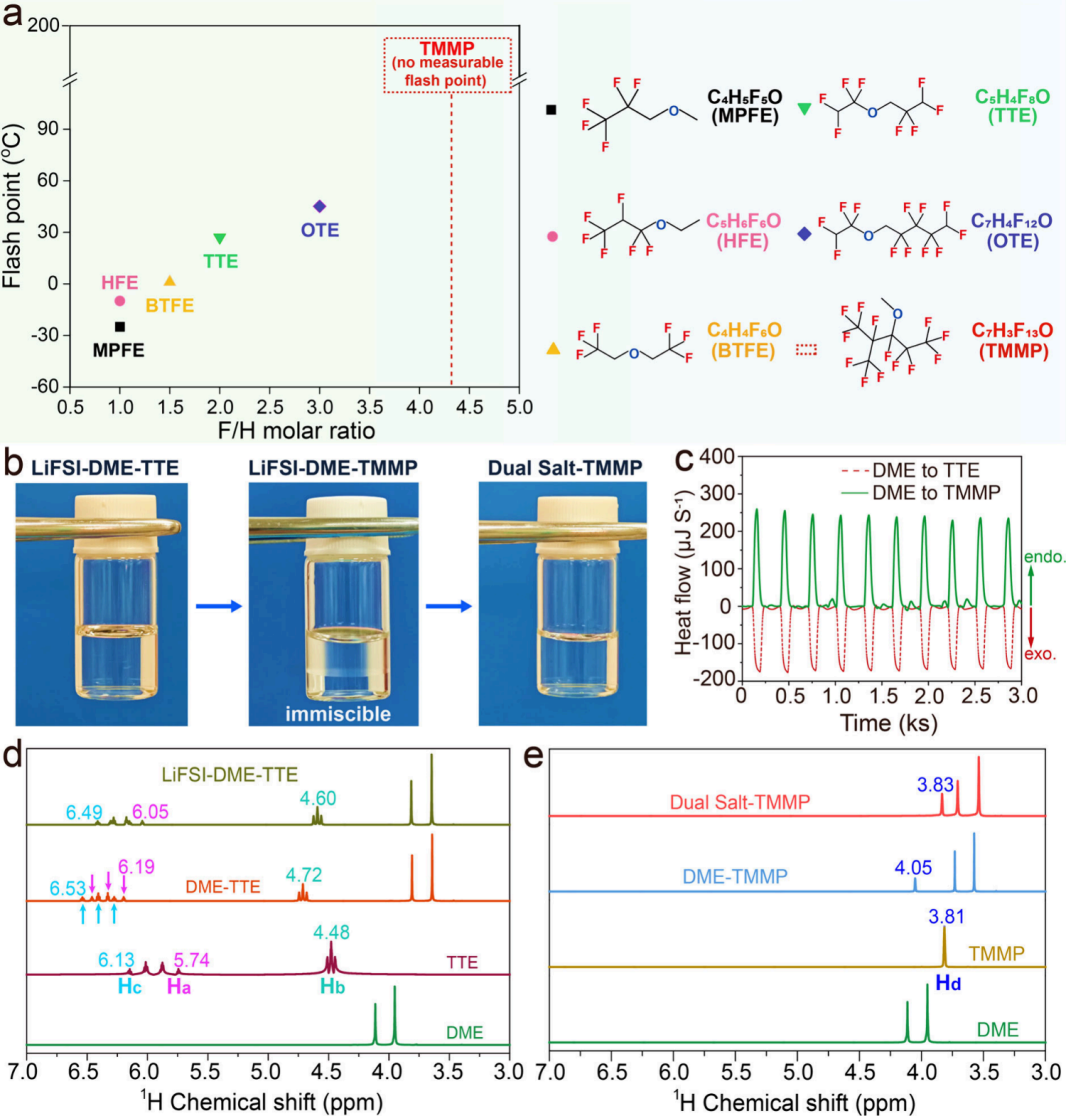
(a) Flashpoints for fluorinated ethers with different F/H molar ratios and their molecular structures. (b) The photos of LiFSI-DME-TTE, LiFSI-DME-TMMP (1:2:2 by molar ratio), and Dual Salt-DME-TMMP electrolytes. (c) Nano ITC data for mixing DME with TTE or TMMP. (d–e) ^1^H NMR spectrum of different diluents, solvent mixture, and electrolytes.

However, phase separation was observed with the direct replacement of TTE diluent with TMMP for the LHCE (denoted as LiFSI-TTE) (Figure 1b). To understand the factors contributing to phase separation, we employed isothermal titration calorimetry (ITC) to measure the differences in thermodynamic interactions during the mixing of DME with TTE and DME with TMMP. As shown in Figure 1c, there was an apparent exothermic process when adding DME into TTE, contrasting with an endothermic process for TMMP. The enthalpy changes during the mixing were calculated from the measured heat for ideal mixing under stirring conditions (Figure S1).^39^ These observations suggest favorable molecular interactions between DME and TTE, corroborated by pronounced chemical shifts in ^1^H NMR and ^17^O NMR spectra (Figure 1d and Figures S2–S3). Upon mixing TTE with DME, the ^1^H signals of TTE shift downfield (Δδ_Ha_ = 0.45 ppm, Δδ_Hb_ = 0.24 ppm, and Δδ_H*c*_ = 0.40 ppm) (Figures 1d and S3) relative to those in pure TTE, whereas the ^1^H signals of DME shift upfield. The ^17^O of DME displays a significant chemical shift of 0.50 ppm (Figure S2), indicating hydrogen bonding in which −CF_2_–H groups of TTE donate protons to the oxygen atoms of DME. These observations are indicative of a strong hydrogen-bonding interaction between TTE and DME.^9^ Moreover, the ^19^F–^1^H heteronuclear Overhauser effect spectroscopy (HOESY) NMR spectrum in Figure S4 also shows the atypical hydrogen-bonding (F···H) interactions between DME and TTE. In contrast, as illustrated in Figure 1e and Figures S5–S6, the smaller chemical shift variation (Δδ_Hd_ = 0.24 ppm) observed upon mixing DME and TMMP suggests weaker hydrogen-bonding interactions compared to the DME-TTE mixture.

Furthermore, LiFSI incorporation into the DME-TTE/DME-TMMP mixed solvents diminishes the ^1^H chemical shift changes of diluents, with strong confinement of solvent molecules within the Li^+^ solvation sheath weakening solvent-diluent interactions (Figure 1d). Despite this, the LiFSI-DME-TTE (1.25:2:2 by mol) system maintains phase stability. In contrast, the already weak interactions between TMMP and DME are further compromised by Li^+^ upon LiFSI addition, resulting in phase separation (Figure 1b). This phenomenon explains why TMMP, despite its successful use as a cosolvent in low concentration electrolytes, faces significant challenges when employed as a diluent in LHCEs. Interestingly, the immiscibility issue was successfully addressed when a fluorous sulfonylimide salt with long fluoro-alkyl moieties, LiBETI, was introduced to replace part of LiFSI, yielding homogeneous Dual Salt-TMMP electrolyte (Figure 1b).

### Effect of Fluorophilic and Hydrogen-Bonding Interactions

We hypothesized that the interactions between anion, solvent and diluent is essential for maintaining the phase stability of the designed electrolyte. To elucidate this relationship, we conducted a comprehensive study combining density functional theory (DFT), Ab initio molecular dynamics (AIMD) simulations, and spectroscopic methods. DFT calculations revealed that fluorophilic interactions are the primary mechanism governing anion-TMMP interactions. Fluorophilic interactions, which are noncovalent attractions between fluorine-rich molecules, have been previously exploited in fields such as materials science and drug design to create unique self-assembling structures and enhance drug–target binding.^40,41^ The number of C–F bonds in anions significantly influences their fluorophilic interactions with TMMP. Among the studied anions (FSI^–^, TFSI^–^, and BETI^–^), DFT calculations indicate a qualitative trend of increasing fluorophilic interactions in the order FSI^–^ < TFSI^–^ < BETI^–^ (Figure 2a). These computed binding energies are small in magnitude and do not account for explicit solvation or entropic effects, and thus should be interpreted as relative trends rather than absolute interaction strengths. Consistently, AIMD simulations also reflect this trend, supporting the relative ordering of fluorophilic interactions (Figure 2b). AIMD simulations further corroborated these findings by analyzing the radial distribution functions (RDFs) of F­(anion) with F­(TMMP). The highest *g­(r)* value observed for BETI^–^ indicates a pronounced structuring of TMMP molecules around BETI^–^ compared to other anions. This strong interaction between BETI^–^ and TMMP in the outer solvation sheath stabilizes the LHCE solvation structure. Importantly, Li^+^ remains tightly coordinated with DME rather than TMMP, as evidenced by strong RDF peaks of Li^+^-O in these electrolytes (Figure S7).

**Figure 2:**
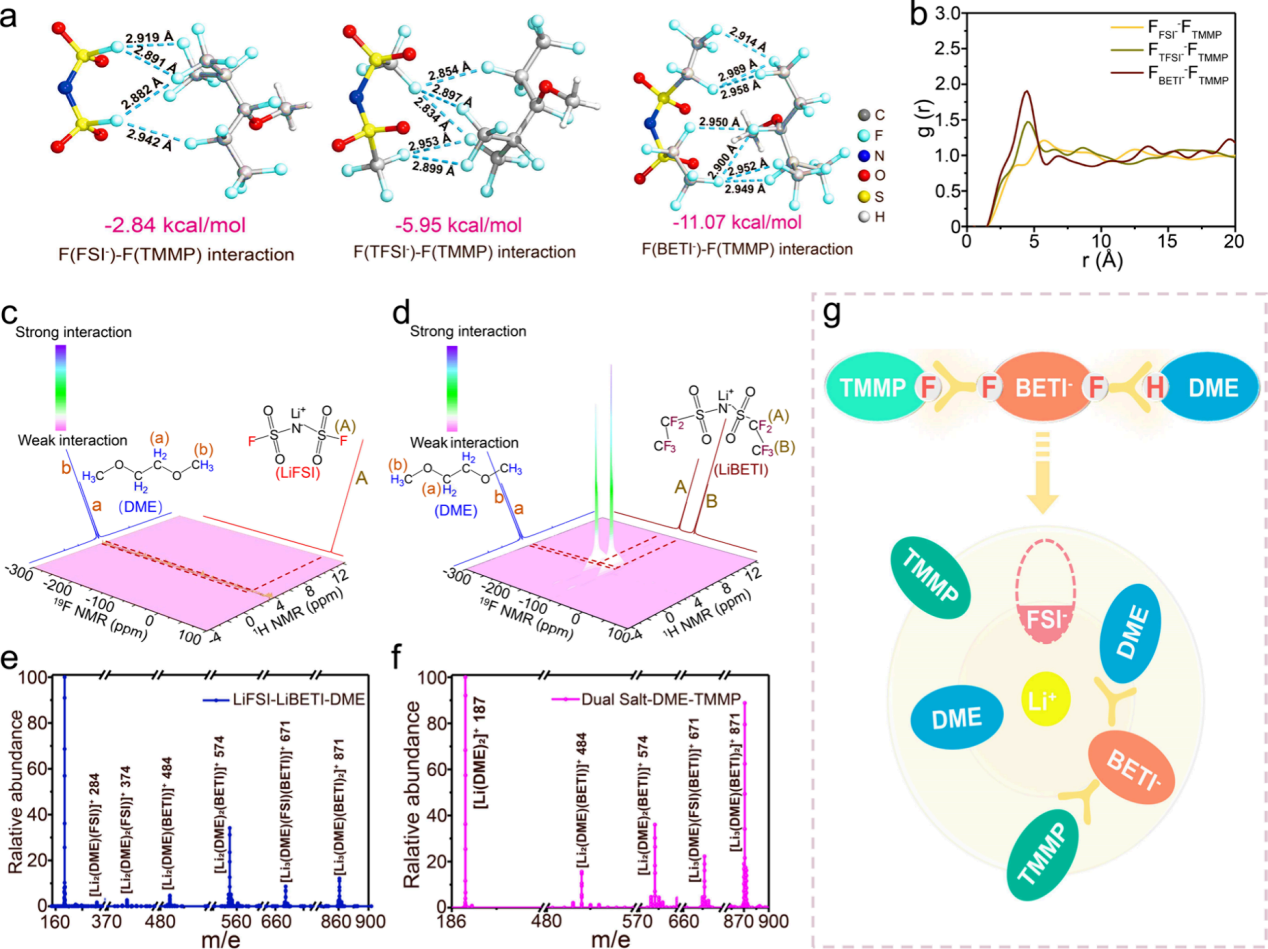
(a) DFT calculations of fluorophilic interactions between different anions and TMMP. (b) Radial distribution functions *g*(*r*) of F_FSI_–F_TMMP_, F_TFSI_–F_TMMP_, and F_BETI_–F_TMMP_. The ^19^F–^1^H HOESY NMR spectrum of (c) LiFSI-DME mixture and (d) LiBETI-DME mixture. ESI-MS characterizations of (e) LiFSI-LiBETI-DME mixture, and (f) Dual Salt-TMMP. (g) Solvation structure of Dual Salt-TMMP electrolyte.

To qualitatively examine the spatial proximity between solvent and anion molecules in the inner solvation sheath, two-dimensional ^19^F–^1^H HOESY NMR spectra were recorded. The analysis revealed a striking contrast between the LiFSI-DME and LiBETI-DME mixtures (Figures 2c-2d). The LiFSI-DME mixture showed no significant HOESY signal, indicating negligible spatial correlation between the FSI^–^ anion and DME solvent molecules (Figure 2c). In contrast, the LiBETI-DME mixture exhibited a distinct HOESY signal, providing clear evidence of a close spatial association between the BETI^–^ anion and DME molecules (Figure 2d). This observation has important implications for our electrolyte design. The strong interaction between BETI^–^ and DME suggests that amphiphilic BETI^–^ modulates the formation of the solvation structure. When TMMP is introduced as a diluent, BETI^–^ can effectively bridge the gap between the solvent (DME) and the highly fluorinated diluent (TMMP), producing a uniformly, miscible electrolyte. The solvation structures in these formulations were further analyzed by electrospray ionization mass spectrometry (ESI-MS) (Figures 2e-2f and Figures S8–S11). Notably, the LiBETI-DME mixture showed significant peaks corresponding to Li^+^-solvent-anion complexes, including [Li_2_(DME)­(BETI)]^+^, [Li_2_(DME)_2_(BETI)]^+^, and [Li_3_(DME)­(BETI)_2_]^+^, suggesting strong hydrogen bond interactions between BETI^–^ and DME (Figures S8). In contrast, the LiFSI-DME mixture exhibited only a weak peak for [Li_2_(DME)­(FSI)]^+^ (Figures S9). The predominant peaks in this system corresponded to Li^+^ ions coordinating independently with either DME or FSI^–^, indicating a lack of apparent interactions between the solvent and anion. Comparative analysis of LiFSI and LiBETI with equal molar ratios in DME revealed a significantly higher relative abundance of BETI^–^-containing solvation species compared to FSI^–^-containing species (Figures 2e and Figure S10). This observation underscores the strong hydrogen bond interactions between BETI^–^ and DME, which promote the formation of stable Li^+^ solvation species containing BETI^–^. Remarkably, as shown in Figure 2f and Figure S11, the Dual Salt-TMMP electrolyte maintained a similar ESI-MS spectrum to the LiBETI-DME mixture, indicating the preservation of these beneficial interactions in the presence of TMMP. These results reveal the critical role of dual interactions - fluorophilic (F···F) and hydrogen bonding (F···H) - among anions, solvents, and fluorinated ethers in resolving the miscibility-flammability dilemma of fluorous ethers in HCEs (Figure 2g). While LiBETI salt has been previously used in low-concentration electrolytes with TMMP cosolvent, this study marks its first application in concentrated electrolytes.^42,43^ Moreover, we elucidate for the first time the mechanism by which LiBETI functions as an amphiphilic mediator, bridging the gap between polar solvents and nonpolar highly fluorinated diluents. This insight provides a novel strategy for designing safe, high-performance electrolytes for advanced battery systems.

To further validate the proposed mechanism and explore its broader applicability, we extended our investigation to include alternative solvents and highly fluorinated diluents. This expanded study encompassed dimethyl carbonate (DMC), methyl nonafluorobutyl ether (MFE, with an F/H molar ratio of 3), and 1H-perfluorohexane (CFH, with an F/H molar ratio of 13), as detailed in Table S2 and Figure S12. The experiments revealed that at lower concentrations of LiFSI, electrolytes containing these fluorous diluents maintained phase stability. Specifically, stable phases were observed in configurations such as LiFSI-DMC-TMMP, LiFSI-DME-TMMP, LiFSI-DME-MFE, and LiFSI-DME-CHF, all at a molar ratio of 0.25:2:2. This stability at lower salt concentrations suggests that the interactions between the solvent and diluent are sufficient to maintain a homogeneous mixture under these conditions. However, with the increase in LiFSI concentration (1.25:2:2 for the above electrolytes), phase separation became evident in all those electrolytes. The onset of phase separation at higher salt concentrations underscores the delicate balance of interactions within these complex electrolyte systems and highlights the challenges in maintaining phase stability as the ionic strength increases. Importantly, the successful resolution of electrolyte phase separation was achieved by the addition of the amphiphilic BETI^–^ anion. These findings indicate the versatility and effectiveness of our dual-salt strategy in addressing the phase separation challenges inherent in highly fluorinated electrolyte systems. We also investigated the maximum soluble amount of LiBETI at different LiFSI concentrations (Figure S13). LiFSI can only dissolve at a molar ratio of 0.25:2:2 in a DME/TMMP mixture solvent, while LiBETI can dissolve up to a ratio of 1.3:2:2 in the DME/TMMP mixture. The results reveal the capacity of BETI^–^ to resolve the immiscibility issue, which acts as a bridge featuring favorable fluorophilic (F···F) interactions with fluorous diluents (TMMP) and atypical hydrogen-bonding (F···H) interactions with the solvent (DME).

### Physical Characterization and Ignition Tests of Various Electrolytes

Room-temperature ionic conductivity (25 °C) of the electrolyte was determined with a BioLogic VMP-3 and calibrated against the resistance of a 1 M KCl standard solution. Table S3 shows that at 25 °C, the ionic conductivity of Dual Salt-TMMP (2.12 mS·cm^–1^) is higher than that of Dual Salt-H (1.95 mS·cm^–1^). Pulsed field gradient NMR (PFG-NMR) measurements at 25 °C provided the self-diffusion coefficients of ^7^Li and ^19^F,^44^ with ^7^Li signals employed to extract the diffusion coefficient (*D*_Li^+^_)) of Dual Salt-H and Dual Salt-TMMP (Table S3). The ion mobility number (*t*_Li_^NMR^) was determined by the formula of $t_{Li}^{NMR}=\frac{D_{Li}}{D_{Li}+D_{anion}}$ . The results in Table S3 indicate that the Dual Salt-TMMP exhibits higher *D*_Li+_ and a *t*_Li_^NMR^ that is nearly the same as that of Dual Salt-H, indicating its enhanced Li^+^ transport. The transport behavior of Li^+^ in the electrolytes was further examined by calculating the mean square displacement (MSD), from which diffusion coefficients were obtained. Typical linear MSD-time curves are presented in Figure S14. Li^+^ exhibits a greater diffusion coefficient in Dual Salt-TMMP compared to Dual Salt-H, reflecting enhanced diffusion kinetics consistent with PFG-NMR results. We also compared the viscosity at 25 °C for both TMMP diluents and the resulting electrolytes with TTE diluent/electrolytes (Table S4). The higher viscosity observed in the TMMP electrolyte originates from the presence of LiBETI rather than from TMMP itself. Despite this increase, its viscosity remains within a range that supports efficient ion transport. Additionally, as for the density of the electrolyte, compared with TTE, TMMP does not apparently increase the density of the electrolyte. The trade-off is justified by the improvements in safety, ensuring that the electrolytes remain effective for high-performance battery applications.

### Electrochemical Performance of Electrolytes

The compatibility of designed electrolyte with Li metal was assessed by measuring the CE of Li plating and stripping via the Aurbach method. Four electrolyte systems were compared: conventional carbonate (1.0 M LiPF_6_ in EC-EMC (3:7 by weight) with 2 wt % VC), LiBETI-DME-TMMP (1:2:2 molar ratio, denoted as “LiBETI-TMMP”), LiBETI-LiFSI-DME (1:0.25:2, denoted as “Dual Salt-H”), and Dual Salt-TMMP, at current densities of 0.5, 1.0, and 2.0 mA cm^–2^. According to Figure 3a, the Dual Salt-TMMP electrolyte achieved a remarkable Li CE of 99.53% at 0.5 mA cm^–2^, surpassing the performance of carbonate (89.26%), LiBETI-TMMP (95.62%), and Dual Salt-H (98.74%) electrolytes. The observed improvement can be attributed to the suppression of lithium metal-DME side reactions by the elevated salt concentration, coupled with the interfacial stabilization provided by LiFSI and LiBETI. In contrast, the LiBETI-TMMP electrolyte exhibits the smallest voltage hysteresis among all systems, likely due to its enhanced wettability and Li^+^ transport kinetics (Figure S15). However, the higher reduction stability of BETI^–^ limits LiF formation, resulting in a less protective SEI and a slightly lower CE. At elevated current densities of 1.0 and 2.0 mA cm^–2^, the Dual Salt-TMMP electrolyte exhibited Li CEs of 99.39% and 99.20% (Figure 3b and Figures S16–S17). In contrast, the CEs of carbonate electrolyte decreased to 85.48% and 82.93%, while the CEs of Dual Salt-H electrolyte dropped to 98.55% and 92.32% under the same conditions. Figure S18 demonstrates that the initial capacity–voltage of Li*||*Cu cells of Dual Salt-TMMP reveals a smaller nucleation overpotential compared to that of Dual Salt-H at various current densities, which is closely related to the nucleation of Li. Reduced nucleation overpotential in Dual Salt-TMMP facilitates Li nucleation and promotes faster Li deposition. The improved Li deposition kinetics of Dual Salt-TMMP may be attributed to its lower viscosity (12.65 mPa·s) compared to Dual Salt-H (32.23 mPa·s), as well as its superior wettability on both the Celgard 2500 separator and Li foil (Figure S15). The cycling stability of Li||Cu cells with different electrolytes was examined via repeated Li plating and stripping on bare Cu foil, as shown in Figures S19–S20.^45,46^ Figure S19 illustrates that the CEs of the carbonate and LiBETI-TMMP electrolytes decrease sharply during the initial 30 cycles, accompanied by a noticeable increase in the voltage gap between lithium plating and stripping (Figures S20a–b). These observations indicate the rapid formation of side products at the electrolyte-Li metal interface. In contrast, the high salt concentration of Dual Salt-H effectively suppresses these side reactions, enabling stable cycling for up to 140 cycles (Figure S20c). Furthermore, the Dual Salt-TMMP electrolyte achieves an average Li-metal Coulombic efficiency exceeding 98.84% and demonstrates superior stability over extended cycling. Notably, as shown in Figure S20d, the polarization during Li plating and stripping remains nearly constant, reflecting the superior stability of the electrolyte. As shown in Figure S21, compared to Dual Salt-H, the reduction peak of Dual Salt-TMMP shifts to a lower potential, indicating improved reduction stability. Moreover, Dual Salt-TMMP demonstrates a lower overpotential for Li deposition and a stronger current response during Li plating/stripping on the Cu substrate, indicating enhanced Li^+^ transport and improved reaction kinetics. Consistently, electrochemical impedance spectroscopy (EIS) measurements (Figures S22–S23) reveal that, compared with the carbonate and Dual Salt-H electrolytes, the Dual Salt-TMMP system exhibits minimal impedance growth and highly stable distribution of relaxation time (DRT) features, further evidencing enhanced interfacial stability and Li plating/stripping behavior. Cycling of Li||Li symmetric cells was used to assess the interfacial stability and compatibility of different electrolytes. As illustrated in Figure S24a, the Dual Salt-TMMP electrolyte exhibits the most stable cycling with minimal polarization over 450 h, while the carbonate and Dual Salt-H electrolytes display gradually increased voltage hysteresis. The magnified views of the first, 10th, and 50th cycles (Figures S24b–d) show that the Dual Salt-TMMP cell maintains the lowest and most stable overpotential during repeated Li plating/stripping, indicating forming the robust and uniform SEI that effectively mitigates dendritic growth and interfacial impedance accumulation. In contrast, the carbonate-based electrolyte suffers from continuous voltage increase, suggesting unstable SEI formation and parasitic reactions, whereas the Dual Salt-H system exhibits intermediate stability. These findings corroborate the Li||Cu results and demonstrate that the TMMP-containing dual-salt system enables the development of a stable, ionically conductive SEI, thereby ensuring superior interfacial reversibility. EIS measurements further confirm this trend (Figure S23 and S25), with the Dual Salt-TMMP electrolyte exhibiting the lowest and most stable interfacial resistance and consistent DRT features over cycling, highlighting its superior ability to maintain a conductive and robust SEI compared with the other electrolytes. As depicted in Figures S26–S27, Dual Salt-TMMP shows stable and reversible plating/stripping behavior across different rates, with an overpotential of approximately 98 mV even at 4 mA cm^–2^. This evidence further validates the cycling stability of the Dual Salt-TMMP electrolyte, providing a more comprehensive assessment of its long-term performance. The smaller overpotentials in these results may be attributed to the generation of Li^+^-DME solvates and a stable LiF-rich SEI in the LHCE, which enhance Li ion transport, reduce desolvation energy, and promote plate-like Li nucleation, leading to smoother deposition.^26,46,47^ Furthermore, the exchange current densities of Li||Li symmetric cells were extracted from the corresponding Tafel plots, and the value for the Dual Salt-TMMP electrolyte (0.25 mA cm^–2^) is higher than for Dual Salt-H (0.14 mA cm^–2^) (Figure S28). This suggests that Dual Salt-TMMP exhibits more rapid charge transfer kinetics. Scanning electron microscopy (SEM) analysis revealed significant morphological differences in lithium deposits across the electrolytes (Figure 3c and Figures S29–S31). The carbonate electrolyte produced loose structures approximately 23 μm thick after 2.0 mAh cm^–2^ Li deposition (Figure S29), likely resulting from continuous parasitic reactions and “dead” Li aggregation. The Dual Salt-H electrolyte yielded large, porous Li metal lumps with a total thickness of ∼21 μm (Figure S30). In contrast, the Dual Salt-TMMP electrolyte facilitated the formation of denser Li deposits (Figure 3c and Figure S31), characterized by compact, nodule-like Li particles with a total thickness of ∼11.16 ± 0.63 μm and no visible dendrites. Collectively, the Dual Salt-TMMP electrolyte exhibits enhanced Li plating/stripping efficiency and improved deposit morphology, highlighting its potential to boost both performance and safety in LMBs. A key safety concern of lithium metal batteries is the propensity of internal battery short-circuiting and thermal runaway, due to the high reactivity of lithium and the flammability of organic electrolytes. Thus, the thermal behavior of the electrolytes was assessed using DSC with Li metal anode. To prevent overpressure from damaging the DSC holder during heating, a small vent was created in the cap. A thin gold-coated stainless-steel foil was placed beneath to limit electrolyte evaporation. This arrangement maintained a sealed environment while allowing excess gas to escape safely. Measurements were carried out using 10 mg of electrolyte and 1 mg of Li, harvested from deposits on Cu foil during Li||Cu cell cycling to closely mimic anode conditions. The samples were heated from 25 to 300 °C at a rate of 5 °C/min. As shown in Figure S32, the DSC curves of Dual Salt-TMMP electrolytes was compared with those of the state-of-the-art LiFSI-TTE electrolyte (LiFSI-DME-TTE, 1:1.2:3 by molar ratio). The LiFSI-TTE electrolyte exhibited exothermic behavior starting around 75 °C, primarily driven by the reactive nature of FSI^–^ anions with Li metal. In contrast, the Dual Salt-TMMP electrolytes exhibited a delayed heat release onset temperature. In addition, above the melting point of lithium metal (∼180 °C), the LiFSI-TTE electrolyte exhibited more pronounced exothermic reaction behavior compared to the Dual Salt-TMMP electrolyte. The fluorous Dual Salt-TMMP system likely inhibits these exothermic reactions, thereby enhancing thermal stability.

**Figure 3:**
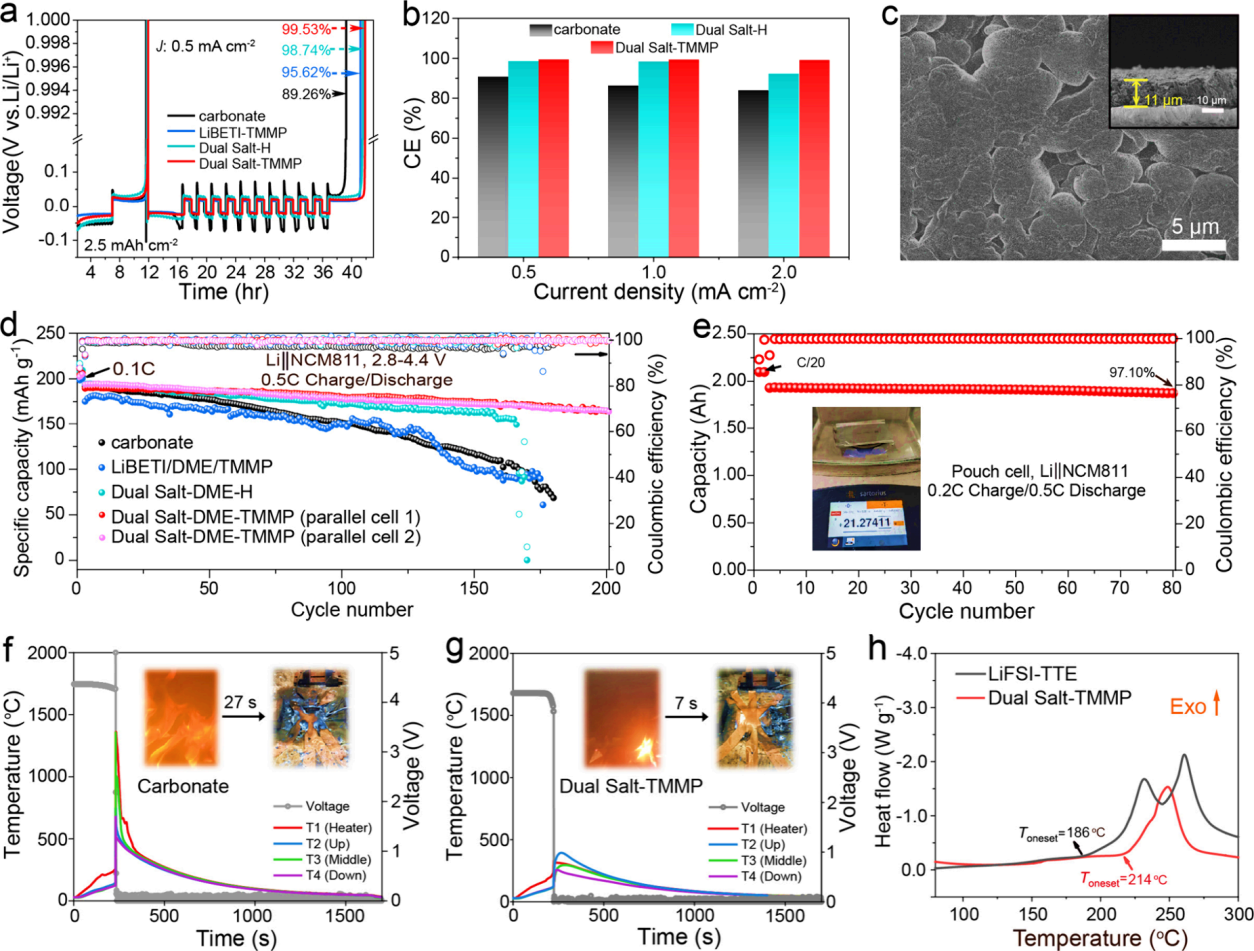
Electrochemical properties of different electrolytes. (a–b) CE tests of Li plating/stripping at selected current densities (0.5–2.0 mA cm^–2^) measured by the Aurbach method. (c) Top and cross-sectional SEM images of Li deposits on Cu in the Dual Salt-TMMP electrolyte (2 mAh cm^–2^, 0.5 mA cm^–2^). (d) The cyclability of Li||NCM811 batteries with a high cutoff voltage of 4.4 V at 30 °C. (e) Cycling performance of the Li||NCM811 pouch cell using Dual Salt-TMMP electrolyte (2.3 g Ah^1–^). Temperature and voltage curves during thermal runaway for Li||NCM811 pouch cells using different electrolytes: (f) carbonate and (g) Dual Salt-TMMP electrolytes. (h) DSC thermograms for delithiated NCM811 with different electrolytes.

To assess the suitability of the designed electrolyte for high-voltage LMBs, the Li||NCM811 batteries were evaluated. All batteries exhibited comparable first-cycle discharge capacities of approximately 200 mAh g^–1^ (based on NCM811) at C/10 during the initial two formation cycles, with an initial CE of 89.31% under a 4.4 V cutoff (Figure 3d). Figure 3d and Figure S33 illustrate that the carbonate electrolyte induces rapid capacity declines and enlarged battery polarizations within 100 cycles. The battery CE gradually drops to below 98% after ∼40 cycles, indicating apparent parasitic reactions on the cathode under high voltage. A similar situation occurs with the LiBETI-TMMP electrolyte, where rapid capacity declines and increased battery polarization are observed within 50 cycles (Figure 3d and Figure S34). It is likely that a LiBETI-only electrolyte appears insufficient to generate a protective electrode interface, leading to continuous side reactions. The Dual Salt-H electrolyte demonstrates enhanced cycling stability, maintaining over 84% of its initial capacity for up to 150 cycles (Figure 3d and Figure S35), suggesting the beneficial role of LiFSI in stabilizing the reactive electrodes. Importantly, the Dual Salt-TMMP electrolyte enables Li||NCM811 batteries to achieve superior cycling performance, with ∼86% capacity retention after 200 cycles and an average CE of 99.72% in replicate experiments (Figures 3d and S36). EIS data presented in Figure S37 suggest that the Dual Salt-TMMP electrolyte effectively suppresses the growth of interfacial resistance over 25 cycles, whereas the carbonate system exhibits a pronounced increase in polarization. Corresponding DRT analysis further confirms that Dual Salt-TMMP minimizes both SEI and charge-transfer related relaxation processes, highlighting improved Li^+^ transport and enhanced interfacial stability at both electrodes. Linear scanning voltammetry (LSV) confirmed the enhanced anodic stability of the Dual Salt-TMMP electrolyte compared to Dual Salt-H electrolyte, with minimal oxidation current at 4.5 V (Figure S38). Moreover, the CV curves for Li||Al cells (scan rate: 0.1 mV s^–1^) using Dual Salt-H and Dual Salt-TMMP electrolytes are shown in Figure S39. Both electrolytes demonstrate no apparent Al corrosion currents up to 4.5 V vs Li^+^/Li, and Al surface passivation were observed in subsequent cycles. Area-normalized constant-potential holds from 4.0 to 4.6 V (vs Li/Li^+^) revealed negligible leakage current for the Dual Salt-TMMP electrolyte, confirming its superior anodic stability compared with Dual Salt-H (Figure S40). ICP-OES analysis after the 4.6 V hold, with samples diluted 83-fold prior to measurement, showed minimal Al dissolution, with concentrations of 0.022 ppm for Dual Salt-TMMP (at the detection-limit level) and 0.052 ppm for Dual Salt-H. These results demonstrate that both electrolytes exhibit extremely low Al corrosion under high-voltage conditions, while Dual Salt-TMMP provides approximately 2.6-fold better suppression of anodic Al dissolution. X-ray diffraction (XRD) analysis of cycled NCM811 cathodes showed no significant structural changes after 200 cycles in both carbonate and Dual Salt-TMMP electrolytes (Figure S41), indicating preserved cathode integrity. The Dual Salt-TMMP electrolyte also enabled superior rate capabilities in Li||NCM811 batteries (Figure S42) and demonstrated good cycling stability in Li||LiCoO_2_ (LCO) batteries at 4.5 V, retaining 95.12% capacity after 100 cycles (Figure S43). Dual Salt-TMMP electrolyte also demonstrates broad temperature adaptability. Under elevated conditions of 45 °C, Li||NCM811 cells with the Dual Salt-TMMP electrolyte preserve 92.87% of their initial capacity over 150 cycles (Figure S44). At low temperatures, decreased ionic conductivity reduces ion mobility, leading to a slight capacity drop to 100–110 mAh g^–1^. However, cycling stability remains strong over 150 cycles (Figure S45).

A practical Li||NCM811 pouch cell, featuring a 50 μm-thick Li foil, high NCM811 loading, and a lean electrolyte amount of 2.3 g Ah^1–^, was employed to further evaluate the cycling stability and safety of electrolyte. As detailed in Table S5, the Li||NCM811 pouch cell containing the Dual Salt-TMMP electrolyte delivered a total capacity of 2.10 Ah and an energy density of 380 Wh kg^–1^ during the first formation cycle at a C/20 discharge rate, calculated based on the total pouch cell mass (Figure S46a). Notably, Figure 3e shows that the pouch cell using the Dual Salt-TMMP electrolyte retains 97.14% of its capacity after 80 cycles and achieves an average CE of 99.69%. Additionally, the voltage curves from various cycles, presented in Figure S46b, demonstrate the stability of the pouch cell, with no apparent voltage–polarization increase during cycling. These results indicate that the Dual Salt-TMMP electrolyte exhibits excellent interfacial compatibility with Li metal and NCM811 cathodes in realistic battery configurations.

The incorporation of TMMP, with its ultrahigh F/H ratio of 4.33 and elevated flash point, significantly enhances the safety properties of the electrolyte. This improvement is clearly demonstrated through comprehensive flammability tests conducted on various electrolyte formulations, as illustrated in Figure S47, and . Conventional carbonate, and the LiBETI-LiFSI-DME dilute electrolyte (1:0.25:10 by molar ratio, denoted as “Dual Salt-D”), exhibit extremely high flammability (Figure S47, and ). This elevated risk of combustion stems primarily from the large content of flammable organic solvents. Even the Dual Salt-H, despite its high salt-to-solvent ratio, remains susceptible to ignition and sustained combustion due to the intrinsic high flammability of DME molecules (Figure S47 and Supplementary Video S3). In stark contrast, the Dual Salt-TMMP electrolyte demonstrates remarkable flame resistance. During prolonged exposure to a torch (exceeding 3 s), this formulation resists ignition, as evidenced in Figure S47 and Supplementary Video S4. This remarkable flame-resistant behavior can be attributed to the large proportion of TMMP in the electrolyte, which exceeds 53% by weight. The presence of TMMP effectively disrupts the chain reactions involved in DME combustion by generating F radicals, which neutralize the H radicals typically responsible for sustaining the combustion process. Moreover, the thermal abuse testing was used to evaluate the safety characteristics of the designed electrolyte.^48^ Prior to testing, the Li||NCM811 pouch cells were charged to 4.4 V at 0.05 C. The cells were then subjected to a constant heating power of 100 W until thermal runaway occurred. Figures 3f-3g illustrates the temperature and voltage curves of Li||NCM811 pouch cells containing different electrolytes (carbonate and Dual Salt-TMMP). Temperature probes were attached to the side of the pouch cell near the heating plate (T1) and three different areas on the other side of the pouch cell away from the heating plate (T2, T3 and T4). During external heating, significant smoke was observed from the pouch cell containing the carbonate electrolyte, revealing serious decomposition of the electrolyte. However, no apparent smoking was noticed for the Dual salt-TMMP electrolyte. Overheating the cells ultimately leads to battery thermal runaway when jet fires were observed from both batteries. However, the flame from the pouch cell with carbonate electrolyte is noticeably brighter and more intense compared to that of Dual Salt-TMMP electrolyte (as shown in ). The temperature probes on the pouch cells also recorded much lower peak temperatures for the pouch cells with the Dual Salt-TMMP electrolyte compared to the carbonate electrolyte (Dual-salt: *T*_2_ = 394 °C, *T*_3_ = 300 °C, *T*_4_ = 255 °C; carbonate: *T*_2_ = 749 °C, *T*_3_ = 1356 °C, *T*_4_ = 676 °C). Additionally, after the thermal runaway took place, the flame on the Dual Salt-TMMP cell body self-extinguished quickly (∼7 s), with only partial damage visible on the cell body. In contrast, the carbonate electrolyte induces a more intense fire, which also persisted significantly longer (∼27 s). Moreover, another critical evidence of high-safety Dual Salt-TMMP electrolyte is their ability to inhibit thermal runaway with the cathode at high states of charge. DSC studies were carried out to evaluate the heat release between charged cathode powder (cutoff at 4.4 V) and different electrolytes, which are tightly sealed in high-pressure crucibles. Apparent differences were first found for their onset exothermic temperatures in different electrolytes (Figure 3h), which are higher in Dual Salt-TMMP (214 °C) than those in the typical LiFSI-TTE electrolyte (186 °C). In addition, the Dual Salt-TMMP system showed significantly reduced heat release than the LiFSI-TTE electrolyte, reflecting its enhanced thermal stability. This improvement can be attributed to the highly fluorinated, flame-retardant TMMP and the formation of a robust cathode–electrolyte interphase (CEI). Consequently, the Dual Salt-TMMP electrolyte has much less exothermic reactions with electrode materials during thermal runaway and demonstrates superior flame-retardant performance, which is beneficial for enhancing the overall safety of LMBs.

### The Electrolyte Chemistry and Interphases

XPS depth profiling analysis was conducted to explore the detailed composition and structure of the SEI layer on Li anodes for cells with the Dual Salt-TMMP, carbonate, and Dual Salt-H electrolytes, providing insights into their interfacial compatibility with Li metal (Figures 4a–c and Figures S48–S51). As shown in Figures 4a–c and S50–S51, the SEI derived from the Dual Salt-TMMP electrolyte contains a markedly higher fraction of inorganic species, particularly LiF, compared with those formed in the carbonate and Dual Salt-H systems, indicating a denser and more stable interphase. In contrast, the SEI formed in the carbonate electrolyte (Figure S49) is dominated by organic components such as ROCO_2_Li and C–O species, with only limited LiF and Li_2_O, suggesting a loose and chemically unstable structure. The enrichment of LiF in the SEI of the Dual Salt-TMMP electrolyte is beneficial, as LiF exhibits excellent electronic insulation and mechanical robustness, effectively limiting parasitic reactions with Li metal.^49^ This observation also suggests that fluorinated anions undergo efficient decomposition at the Li surface, as observed in previous studies of localized high-concentration electrolytes.^50,51^ In addition, the XPS spectra of S 2p and N 1s (Figures S50–S51) confirm the presence of inorganic components such as SO_*x*_, N-SO_*x*_, Li_3_N, and Li_2_S in both the Dual Salt-H and Dual Salt-TMMP systems, further contributing to the stability of the SEI. Overall, these results confirm that the Dual Salt-TMMP electrolyte enables the formation of a robust, inorganic-rich SEI that ensures superior interfacial stability against Li metal. DFT calculations were performed to qualitatively examine the electronic characteristics of the electrolyte components. As shown in Figure S52, TMMP exhibits a relatively high reduction potential compared with FSI^–^ and BETI^–^, suggesting its intrinsically higher susceptibility to reduction. However, due to the absence of TMMP in the inner solvation structure, its contribution to SEI formation is minimal. In contrast, when FSI^–^ and BETI^–^ are coordinated with Li^+^, their reduction potentials decrease markedly. These results qualitatively support that the observed inorganic species mainly originate from anion-derived decomposition. The inorganic-rich SEI layer, enriched in LiF and other ion-conductive species, plays a crucial role in stabilizing the Li metal anode and improving its electrochemical performance in the Dual Salt-TMMP electrolyte.

**Figure 4:**
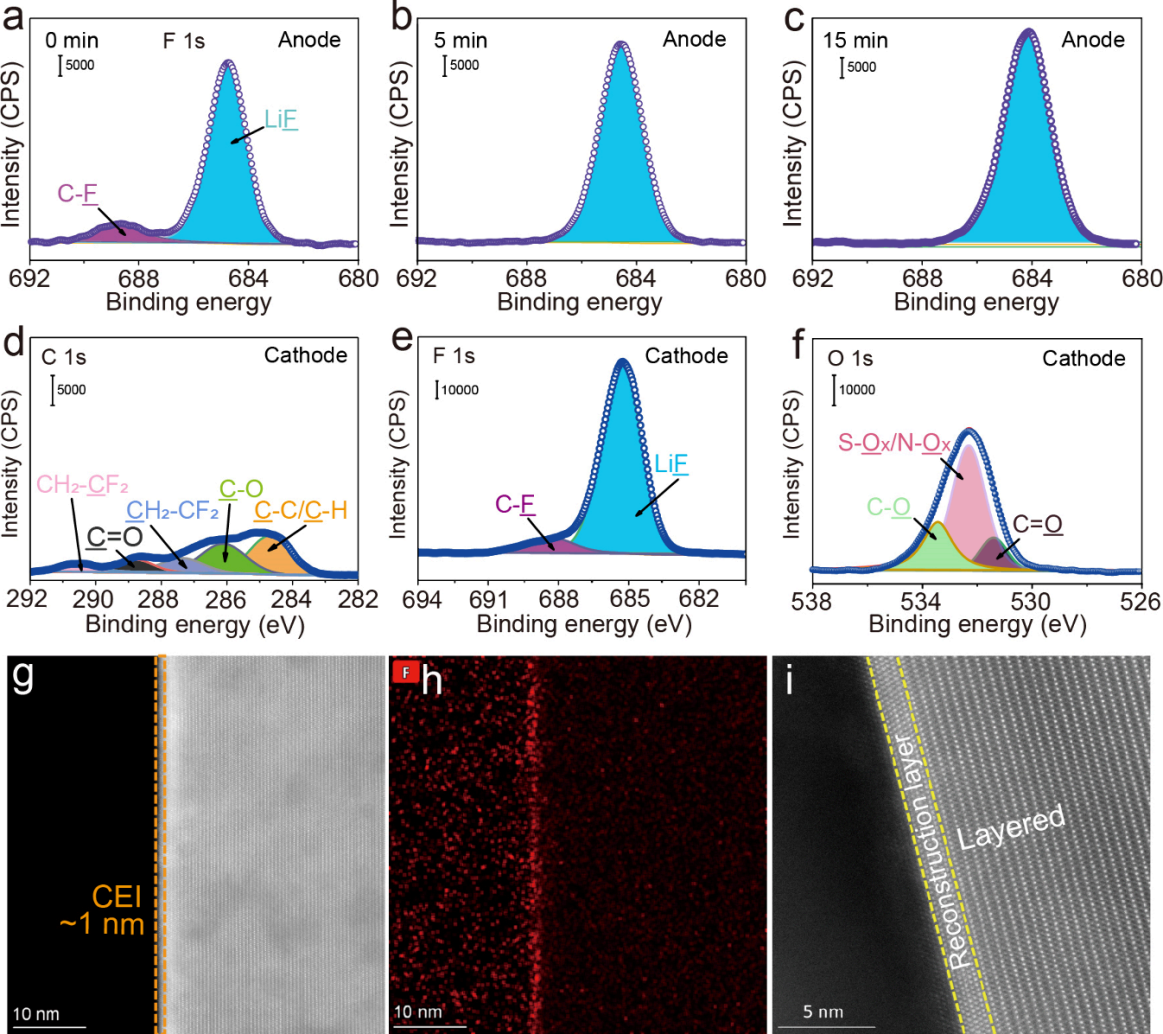
Surface analyses performed on cycled Li anodes and cathode. (a–c) The F 1s XPS depth profiles of the Li anodes in Dual Salt-TMMP electrolyte. (d–f) The XPS spectra of C 1s, F 1s and O 1s for NCM811 after 200 cycles in the Dual Salt-TMMP electrolyte. (g–i) HR-STEM imaging and EDS mapping results of the CEI layers and surface structures on NCM811 cycled in Dual Salt-TMMP electrolyte.

On the cathode side, the formation mechanism of the CEI primarily involves the decomposition of the electrolyte and limited transition metal dissolution from the cathodes. XPS analysis reveals that the CEI formed in the Dual Salt-TMMP electrolyte is predominantly composed of inorganic species, especially LiF (Figures 4d–f). LiF is predominantly produced through lithium salt decomposition, consistent with the presence of N–O_*x*_ species in the CEI (Figure S56). Additionally, TMMP may also contribute to LiF formation through oxidative decomposition under high-voltage conditions. In addition, TMMP can also participate in LiF generation through oxidative decomposition at high voltages, as its TMMP-anion complex exhibits a lower-oxidation potential than the isolated TMMP molecule (Figure S52). In contrast, the CEI formed in the carbonate and Dual Salt-H electrolytes (Figures S54–S55) contains a higher fraction of organic species such as ROCO_2_Li and C–O components, along with weaker LiF and N-SO_*x*_ signals, indicating a less stable interphase. These findings demonstrate that the Dual Salt-TMMP electrolyte establishes a Li-rich and durable CEI, which effectively stabilizes the cathode–electrolyte interface under high-voltage conditions. Notably, the CEI in the Dual Salt-TMMP electrolyte exhibits a markedly higher atomic ratio of lithium (Figure S53), indicating efficient electrolyte decomposition and the consequent formation of a protective layer. The absence of pronounced metal–oxygen (M-O) signals on the cycled NCM811 surface (Figure 4f) suggests effective suppression of transition metal dissolution. Inductively coupled plasma mass spectrometry (ICP-MS) measurements of transition metal ion concentrations on the Li metal anode after cycling (Figure S57) further confirm the role of CEI in mitigating Ni, Co, and Mn dissolution. After 50 cycles, the levels of these metals on the Li anode are significantly lower in the Dual Salt-TMMP system compared to the Dual Salt-H electrolyte.

Scanning transmission electron microscopy (STEM), combined with high-angle annular dark-field (HAADF) imaging and energy-dispersive X-ray spectroscopy (EDS) elemental mapping (Figures 4g–i and Figure S58), was employed to analyze the structural and compositional features of the CEI. To avoid artifacts arising from electrolyte precipitates on secondary particles, analysis was focused on the grain boundaries at the primary particle surfaces. An ultrathin (∼1 nm), dense, and uniform CEI formed in the Dual Salt-TMMP electrolyte provides robust cathode surface protection (Figure 4g). Correspondingly, a mitigated layered-to-disordered rock salt phase transition (∼1 nm) is observed on the NCM811 surface (Figure 4i), suggesting improved structural robustness. In summary, the inorganic-dense and uniformly distributed CEI generated by Dual Salt–TMMP effectively stabilizes the cathode, reduces metal dissolution, and balances electronic insulation with ion conductivity. This unique CEI composition and structure simultaneously reduce electrolyte corrosion of NCM811 and promote Li^+^ transport at the interface, enabling stable cycling under high-voltage conditions (Figure S59).

## Conclusions

In summary, this work introduces a strategy to enhance the safety and electrochemical performance of high-energy-density lithium metal batteries by designing miscible fluorous electrolytes based on amphiphilic anion chemistry. By employing anions with fluoro-alkyl moieties, specifically BETI^–^, we successfully bridged the gap between Li^+^-solvating solvents and highly fluorinated diluents, resolving the critical issue of immiscibility in fluorous electrolytes. The Dual Salt-TMMP electrolyte exhibits remarkable lithium metal reversibility, achieving a high CE of 99.53%, and enables stable cycling of Li||NCM811 cells with 87% capacity retention after 200 cycles at 4.4 V, facilitated by LiF-rich electrode–electrolyte interphases. It also shows enhanced thermal stability and flame retardancy, addressing key safety concerns. This amphiphilic anion approach provides new opportunities for tailoring electrolyte compositions to meet the demands of next-generation energy storage systems. Looking ahead, rational anion design can further improve miscibility and electrochemical performance. Beyond fully fluorinated anions like BETI^–^, amphiphilic or partially fluorinated motifs can balance polarity and fluorophilicity, regulate Li^+^ coordination, and enhance compatibility with solvents such as TMMP. Although highly fluorinated components are beneficial for electrochemical stability and battery safety, they are often associated with high cost and significant environmental concerns. Therefore, future efforts should focus on developing green recycling strategies, designing degradable fluorinated species, or exploring fluorine-free molecular alternatives to achieve sustainable electrolyte systems for safe and high-energy batteries.

## Supplementary Material

















## Notes

Additional NMR spectra, MD simulation snapshots, ESI-MS, SEM images, TEM images, XRD and Raman results, additional electrochemical results (PDF)
Video S1: Ignition test of a carbonate-based electrolyte under an open flame (MP4)
Video S2: Ignition test of a LiBETI–LiFSI–DME electrolyte under an open flame (MP4)
Video S3: Ignition test of a Dual Salt-H electrolyte under an open flame (MP4)
Video S4: Ignition test of a Dual Salt-TMMP electrolyte under an open flame (MP4)
Video S5: Thermal abuse test of Li||NCM811 pouch cells with a carbonate-based electrolyte (MP4)
Video S6: Thermal abuse test of Li||NCM811 pouch cells with a Dual Salt-TMMP electrolyte (MP4)
Transparent Peer Review report available (PDF)
