# Supplementary material for: Molecularly Engineered Amphiphilic Anions Enable Flame-Retarding Fluorous Electrolytes for Lithium Metal Batteries
Source: ACS Cent Sci. 2025 Dec 25;12(1):63–74. doi: 10.1021/acscentsci.5c01711 (PMC12856666; doi:10.1021/acscentsci.5c01711)
Supplement: Supplementary file 1 [file oc5c01711_si_001.pdf]

Supporting Information

Molecularly Engineered Amphiphilic Anions Enable Flame-Retarding Fluorous Electrolytes for Lithium Metal Batteries

Li Chen[‖,1,2], Jiajia Fan[‖,1], Xuan Luo[1], Hehe Zhang[3], Digen Ruan[1], Yuxuan Li[4], Shunqiang Chen[1], Lijiang Tan[1], Qingshun Nian[1], Bingqing Xiong[1], Zihong Wang[1], Jun Ma[1], Shuping Wang[5], Yifeng Cheng[5], Qingsong Wang[4], Qiang Zhao[6], Zhuo Kang[7,*], Lianfeng Zou[3,*] and Xiaodi Ren[1,*]

[1]Hefei National Research Center for Physical Sciences at the Microscale, School of Chemistry and Materials Science, University of Science and Technology of China, Anhui 230026, China.
E-mail: xdren@ustc.edu.cn (Xiaodi Ren)

[2]Institutes of Physical Science and Information Technology, Anhui University, Hefei, 230601, China.
[3]Clean Nano Energy Center, State Key Laboratory of Metastable Material Science and Technology, Yanshan University, Qinhuangdao, 066004 China.
E-mail: zoulf@ysu.edu.cn (Lianfeng Zou)

[4]State Key Laboratory of Fire Science, University of Science and Technology of China, Hefei, 230601, China.
[5]State Grid Anhui Electric Power Research Institute, State Grid Laboratory of Fire Protection for Transmission and Distribution Facilities, Anhui Province Key Laboratory of Electric Fire and Safety Protection, Hefei 230601, China.
[6]School of Chemical Engineering, Sichuan University, Chengdu 610065, China
[7]Academy for Advanced Interdisciplinary Science and Technology, Beijing Key Laboratory for Advanced Energy Materials and Technologies, State Key Laboratory for Advanced Metals and Materials and Beijing Advanced Innovation Center for Materials Genome Engineering, School of Materials Science and Engineering, Key Laboratory of Advanced Materials and Devices for Post-Moore Chips Ministry of Education, University of Science and Technology Beijing, Beijing 100083, China

E-mail: zhuokang@ustb.edu.cn (Zhuo Kang)

[‖]These authors contributed equally to this work

Experimental Section

Materials: Li foils (Φ = 15.6 mm, thickness = 450 μm) were purchased from China Energy Lithium Co. Ltd. The ethylene carbonate (EC), diethyl Carbonate (DEC), methyl ethyl carbonate (EMC), and vinylene carbonate (VC) were purchased from Sigma-Aldrich. The TMMP was purchased from Aladdin Co. Ltd. All solvents (chromatography grade, anhydrous, ≥99.9%) were thoroughly dried over 4 Å molecular sieves for at least 72 h before use to remove trace moisture. Battery-grade $LiPF_6$ and LiFSI were purchased from DodoChem Co. Ltd. The LiBETI was purchased from the Tokyo Chemical Industry. Both LiFSI and LiBETI salts were dried in a vacuum oven inside an Ar-filled glovebox at 100 °C for 72 h prior to use. The 2 Ah-class NCM811||Li pouch cell, a dry cell supplied by EVE Battery Ltd., was used in the experiment.

Electrolyte preparation: The electrolyte was prepared by the formula of 1.0 M $LiPF_6$ in EC/EMC (3:7 by weight) + 2 wt% VC. The electrolytes from the control experimental were prepared by adding the LiBETI and LiFSI into the DME with a molar ratio of LiBETI-LiFSI-DME at 1: 0.25: 2 (denoted as “Dual Salt-H”) and 1: 0.25 :10 (denoted as “Dual Salt-D”), respectively. Then the electrolyte of Dual Salt-TMMP was prepared with a molar ratio of LiBETI-LiFSI-DME-TMMP at 1: 0.25: 2: 2. All the electrolytes were stored in an argon-filled glovebox (Vigor, oxygen < 0.1ppm, water < 0.1ppm).

Characterizations: The ionic conductivity of the electrolyte at room temperature was measured using a BioLogic VMP-3 and calculated based on the resistance of 1 M KCl at 25 °C. Ion mobility and diffusion coefficients were measured using Pulsed Field Gradient NMR (PFG-NMR). ^{1}H-NMR and ^{19}F-NMR spectra were achieved at 400 MHz using a Bruker AC300 instrument. The nuclear magnetic tube with a capillary containing the deuterated solvent was used to avoid its influence on the solvation structure of the electrolyte. HOESY experiments were performed on a JNM-ECZ600R/S1 spectrometer at 14.09637 T (600 MHz for ^{1}H) and 298

K, using a ^{1}H-observed/ ^{19}F-irradiated HOESY pulse sequence. The mixing time was fixed at 1 s, which is a commonly adopted parameter in many small-molecule HOESY studies. The acquisition matrix comprised 1024 points for 1H and 128 points for ^{19}F, with 4 scans on the ^{1}H dimension and no scans on the ^{19}F dimension. Spectral widths and digital resolutions were 11.28159 kHz/ 11.01718 Hz for ^{1}H and 227.27273 kHz/ 1.77557 kHz for ^{19}F, with offsets of 5 ppm (^{1}H) and -100 ppm (^{19}F). The cross-sections of Li metal and the surface morphology of the recycled Li metal were investigated on a ZEISS Gemini SEM 450. Before characterizations, all samples were rinsed with pure DME to remove residual salts, then dried under vacuum. The cycled Li metal and cathodes were analyzed using a Physical Electronics Quantera scanning X-ray microprobe with a focused monochromatic Al *Kα* X-ray source. Prior to XPS measurement, the electrodes were gently rinsed several times with anhydrous DME inside an Ar-filled glovebox to remove residual electrolyte. The samples were then transferred to the XPS instrument using a self-designed hermetically sealed container filled with argon gas, preventing exposure to ambient oxygen and moisture. X-ray diffractometer (XRD) analysis of cathode materials was performed on a Bruker D8 Venture. Raman spectroscopy (LABRAM, HR) with 785 nm laser excitation was used to evaluate the solvation structure of different electrolytes. For the flammability test, glass microfiber filters (Whatman) soaked with electrolytes were directly exposed to a torch flame. For DSC tests, to prevent the sample holder from rupturing and to avoid potential damage to the instrument caused by excessive pressure during heating, a hole was created in the cap. Additionally, to reduce electrolyte loss from thermal evaporation, a thin layer of stainless-steel foil coated with gold was placed beneath the cap. This foil seals the electrolyte effectively but is designed to break under high gas pressure, allowing for controlled pressure release. The DSC data was obtained by adding 10 mg of electrolyte and 1 mg of NCM811 electrode material into tightly sealed high-pressure crucibles before heating at a rate of 5 °C/ min from room temperature to 350 °C. The heat production and heat absorption of solvent-dilute mixing were monitored by Nano isothermal titration calorimetry (Nano ITC)

(TA Instruments). DME-to-TTE or DME-to-TMMP titration was conducted at 25 °C with per injection volume of 2 μL and the titration interval of 300 s. TTE or TMMP to different salt-solvents (LiFSI-DME, LiTFSI-DME, and LiBETI-DME by molar ratio of 1:2) titrations were conducted at 25 °C with per injection volume of 2 μL and the titration interval of 300 s. The molar density of DME, TTE, and TMMP is 0.00962, 0.006606, and 0.00478 mol·mL^{-1}, respectively. TEM sample preparation and characterizations: the as-prepared cathode samples were randomly selected and coated by Pt with a thickness of ~ 1 μm to protect the surface from ion beam damage. Then, the regions of interest were milled step by step using Ga-ions till reaching electron transparency, followed by a final polishing at the voltage of 5 kV and 2 kV and beam current of 48 pA and 27 pA to remove the beam-damaged layers. The HAADF-STEM imaging and EDS mapping were performed on the Themis Z, equipped with a probe corrector, and operated at a voltage of 300 kV. The HAADF-STEM imaging data were collected at the convergence angle of 17.9 mrad and the collection angle of 41-200 mrad. An optimized beam current of 30-40 pA was used to guarantee the best image quality while minimizing the beam damage.

The flash point of TMMP was determined using a Pensky-Martens closed-cup method according to ASTM D6450, with 1 mL of sample placed in a micro-scale closed cup to minimize evaporation. Measurements were carried out at ambient pressure (~101 kPa) in air at 25 ± 2 °C, and ignition was initiated via an electric arc following the instrument protocol. Each measurement was repeated three times. The torch-flame test was conducted on 1.0 mL of electrolyte, which was first soaked into 19 mm diameter glass fiber discs and then placed in open glass dishes (30 mm diameter) at 25 ± 2 °C under ambient pressure. Each test was performed in triplicate to ensure reproducibility. The thermal-abuse test was performed on a fully assembled pouch cell (2.3 Ah g^{-1}) subjected to external heating using a ceramic-plate heater at a constant rate of 5 °C·min^{-1} under ambient conditions (room air, ~25 °C, relative humidity ~40-50%). K-type thermocouples (TCs) were used to monitor temperature: one TC

(T1) was attached to the side of the pouch cell directly adjacent to the heating plate, while three additional TCs (T2, T3, T4) were placed on the opposite side at distinct locations (center, upper edge, lower edge) to capture spatial temperature gradients. The sensor placement was chosen to monitor both the direct heating interface and the opposing surface, providing a comprehensive picture of thermal progression. Thermocouples were affixed with high-temperature adhesive and secured to prevent displacement during heating.

Electrochemical measurements: LSV, Tafel plots, and CV tests were carried out on BioLogic VMP-3. The electrochemical tests of Li||Cu, Li||Li, Li||NCM811, and Li||LCO were conducted using LANHE battery testers at 30 °C in environmental chambers, with Li foil as the anode. During the assembly of Li||Li cells, a spring clip was used, along with a 0.5 mm spacer, two 450 µm Li foils, and a Celgard 2500 separator with a thickness of 25 µm. For the assembly of Li||Cu cells, a spring clip was used, along with a 1 mm spacer, one 450 µm Li foil, a Celgard 2500 separator, and a 19 µm Cu foil. The oil press of all the batteries was set to 850 psi, with no additional pressure applied. The evaluation of battery performance for Li||Cu batteries is through the previously reported.[1] For Li||Cu batteries, the effective area of the Cu foil disk (thickness = 19 µm) was 2.11 cm^2 with a diameter of 19 mm for Li deposition. The 2032-type coin cell was assembled using a Li foil as the anode, Celgard 2500 as the separator, and NCM811 as the cathode in the glove box. The NCM811 cathode slurry was prepared by mixing NCM811 powder, Super C65, and polyvinylidene fluoride (PVDF) binder in a weight ratio of 80:10:10, with the PVDF first dissolved in N-methyl-2-pyrrolidone (NMP) to form a transparent paste. The resulting slurry was then coated onto Al foil using a doctor blade (Φ = 12 mm, cathode loading = 10 mg ± 0.4 mg/cm^2). Finally, the coated electrodes were dried at 90 °C overnight and punched. 75 µL of electrolyte was added to each cell mentioned above.

Theoretical Calculations: All density functional theory (DFT) computations were performed using the Gaussian 16 package. All solvents and anions were first optimized at the B3LYP/6-311G + (d, p) level with the D3 dispersion correction. Frequency analyses were carried out to confirm that all optimized structures correspond to true energy minima without imaginary vibrational modes. The energy and orbital levels of various molecules were evaluated at the same B3LYP/6-311G + (d, p) level.[2-5] The binding energy between TMMP and various anions was calculated using the B3LYP functional and the 6-311G ++ (d, p) basis set. The stable structures of three counterparts, namely, TMMP + FSI^-, TMMP + $TFSI^-$, and TMMP + $BETI^-$ were rigidly scanned concerning the F-F distance from 1.0 Angstrom to 5.0 Angstrom with the step of 0.1 Angstrom. For reduction potential calculation, the SMD solvation model (e = 7.2) was used to account for the solvation environment. The reduction potential with respect to Li^+/Li^0 was calculated using the equation:

$$E\left(vs.\frac{Li}{Li^+}\right) = -\frac{\Delta G}{F} - 1.4$$

where ΔG is the Gibbs free energy change for the one-electron reduction reaction and F is the Faraday constant.

The molecular dynamics (MD) simulations were also used to characterize the coordination in the studied ether-based electrolytes.[6, 7] The classical MD simulations were performed using the Forcite module in Materials Studio with the COMPASS III force field. A charge-scaling factor of 0.7 was applied to both the cation (Li^+) and anions. The amorphous cell was constructed as a cubic cell with a side length of approximately 30 Å, into which the electrolyte components were packed according to the desired stoichiometry. After geometry optimization and annealing, the systems were first equilibrated for 1.0 ns in the isothermal-isobaric (NPT) ensemble at 303 K, followed by a 1.0 ns production run in the canonical (NVT) ensemble. The mean-squared displacement (MSD) and radial distribution functions (RDFs) were obtained from the converged portion of the production trajectories. Ab initio molecular dynamics (AIMD)

simulations were performed using the Vienna Ab initio Simulation Package (VASP) with projector-augmented wave (PAW) potentials and the Perdew–Burke–Ernzerhof (PBE) generalized gradient approximation (GGA). The simulations were conducted for 50 ps at 300 K in the NVT ensemble using a Nosé thermostat (damping parameter 2.0) and a 0.5 fs time step.

Table S1. The detailed physical properties for various diluents.

diluents	molecular weight (g/mol)	boiling point (°C)	melting point (°C)	flash point (°C)	density	viscosity (mPa·s)	refractive index	molecular orbital energy (LUMO/HOMO) (eV)
methyl 2,2,3,3,3-pentafluoropropyl ether (MPFE)	164.08	48	/	-25	1.29	470	/	-8.05/-0.17
1,1, 2,3,3,3-hexafluoropropyl ethyl ether (HFE)	196.09	65	/	-10	1.32	/	1.35	-9.25/-0.15
bis(2,2,2-trifluoroethyl) ether (BTFE)	182.07	64	/	1	1.42	/	1.28	-8.77/-0.42
1,1,2,2-tetrafluoro-3-(1,1,2,2-tetrafluoroethoxy)propane (TTE)	232.07	92	-94	27	1.54	/	1.29	-9.82/-0.15
1H,1H,5H-perfluoropentyl-1,1,2,2-tetrafluoroethylether (OTE)	332.09	133	-93	45	1.66	5.3	1.30	-9.90/-0.62
2-trifluoromethyl-3-methoxyperfluoropentane (TMMP)	350.07	97.8	-38	No flash point	1.67	3.0	1.27	-8.93/-0.87

Table S2. The configuration methods and states of various electrolytes.

Various electrolyte composition	phase separation (Yes or No)
LiFSI-DMC-TMMP, 0.25:2:2 by molar ratio	No
LiFSI-DMC-TMMP, 1.25:2:2 by molar ratio	Yes
LiBETI-LiFSI-DME-TMMP, 1:0.25:2:2 by molar ratio	No
LiFSI-DME-TMMP, 0.25:2:2 by molar ratio	No
LiFSI-DME-TMMP, 1.25:2:2 by molar ratio	Yes
Dual Salt-TMMP, 1:0.25:2:2 by molar ratio	No
LiFSI-DME-MFE, 0.25:2:2 by molar ratio	No
LiFSI-DME-MFE, 1.25:2:2 by molar ratio	Yes
LiBETI-LiFSI-DME-MFE, 1:0.25:2:2 by molar ratio	No
LiFSI-DME-CHF, 0.25:2:2 by molar ratio	No
LiFSI-DME-CHF, 1.25:2:2 by molar ratio	Yes
LiBETI-LiFSI-DME-CHF, 1:0.25:2:2 by molar ratio	No

Table S3. The physical properties of the studied electrolytes (25 °C)

Electrolytes	Ionic conductivity (σ) (mS cm^{-1})	Ion mobility number (t_{Li^+}) (PFG-NMR)	Diffusion coefficient (D_{Li^+}) (m^2/s)	Ion mobility number (t_{Li^+}) (Bruce-Vincent)	$\sigma \cdot t_{Li^+}$ (mean ± propagated SD, mS·cm^{-1})
Dual Salt-H	1.38 ± 0.03	0.53	1.36×10^{-11}	0.52 ± 0.02	0.72 ± 0.03
Dual Salt-TMMP	7.18 ± 0.08	0.56	6.98×10^{-11}	0.60 ± 0.05	4.31 ± 0.36

Note： The ionic conductivity (σ) of each electrolyte was measured three times independently at a fixed temperature of 25 °C using a calibrated conductivity meter. The reported values are presented as mean ± standard deviation (mean ± SD) to reflect experimental reproducibility. The propagated uncertainty for the derived parameter $\boldsymbol{\sigma} \cdot \boldsymbol{t}_{\mathbf{Li^+}}$ (Li^+) has also been calculated and incorporated into the Table S3 using standard error-propagation:

$$\boldsymbol{X} = \boldsymbol{\sigma} \cdot \boldsymbol{t}_{\mathbf{Li^+}}, \Delta\boldsymbol{X} = \boldsymbol{X}\sqrt{(\frac{\Delta\boldsymbol{\sigma}}{\boldsymbol{\sigma}})^2 + (\frac{\Delta\boldsymbol{t}_{\boldsymbol{Li^+}}}{\boldsymbol{t}_{\boldsymbol{Li^+}}})^2}$$

The resulting propagated uncertainties of $\boldsymbol{\sigma} \cdot \boldsymbol{t}_{\mathbf{Li^+}}$ (Li^+) are relatively small (e.g., 0.03 for Dual Salt-H), confirming the accuracy and stability of the derived values in reflecting electrolyte ionic transport properties. These results are consistent with the cross-validation between Bruce-Vincent and PFG-NMR measurements, demonstrating the reliability and reproducibility of the Li-ion transport parameters. This rigorous statistical treatment further reinforces the validity of our conclusions regarding Li^+ mobility and overall electrolyte performance.

Table S4. Viscosity data for the studied solvents and electrolytes at 25°C (All values represent the average of three independent measurements)

Solvents/Electrolytes	Viscosity (mPa·s)	Density (g/cm^3)
TTE	1.43 ± 0.02	1.54 ± 0.01
TMMP	1.38 ± 0.01	1.67 ± 0.02
LiFSI-DME-TTE (1.25:2:2 by molar ratio)	7.18 ± 0.04	1.32 ± 0.02
LiFSI-LiBETI-DME-TTE (0.25:1:2:2 by molar ratio)	11.85 ± 0.03	1.30 ± 0.01
Dual Salt-TMMP	12.65 ± 0.02	1.31 ± 0.01

Table S5. Cell parameters of the Li||NCM811 pouch cell

Cell component	Details
Cathode	NCM811 with Al collector<br>12 pieces of double-side<br>2.3 mAh cm^{-2} for each-side<br>12 μm for Al foil thickness
Separator	PE (25 μm)
Anode	free-standing 50 μm Li for double side
N/P ratio	2.2
E/C ratio	2.3 g Ah^{-1}

Table S6. Maximum solubility of LiBETI with varying LiFSI concentrations

No.	Electrolyte configuration (LiFSI:LiBETI:DME:TMMP by molar ratio)
Ely1	0.25:0:2:2
Ely2	0.25:1.10:2:2
Ely3	0.20:1.15:2:2
Ely4	0.15:1.20:2:2
Ely5	0.10:1.25:2:2
Ely6	0.05:1.3:2:2
Ely7	0:1.3:2:2

Table S7. Onset and peak temperatures of pouch cells under external heating measured at different positions.

Pouch cells	Temperature (°C)	T_1 (Heater)	T_2 (Up)	T_3 (Middle)	T_4 (Down)
carbonate	Onset	28.3	28.2	26.2	28.2
	Peak	1360	747.3	1357	678.2
Dual Salt-TMMP	Onset	29.1	28.9	26	29
	Peak	334.6	393.9	295.8	256.8

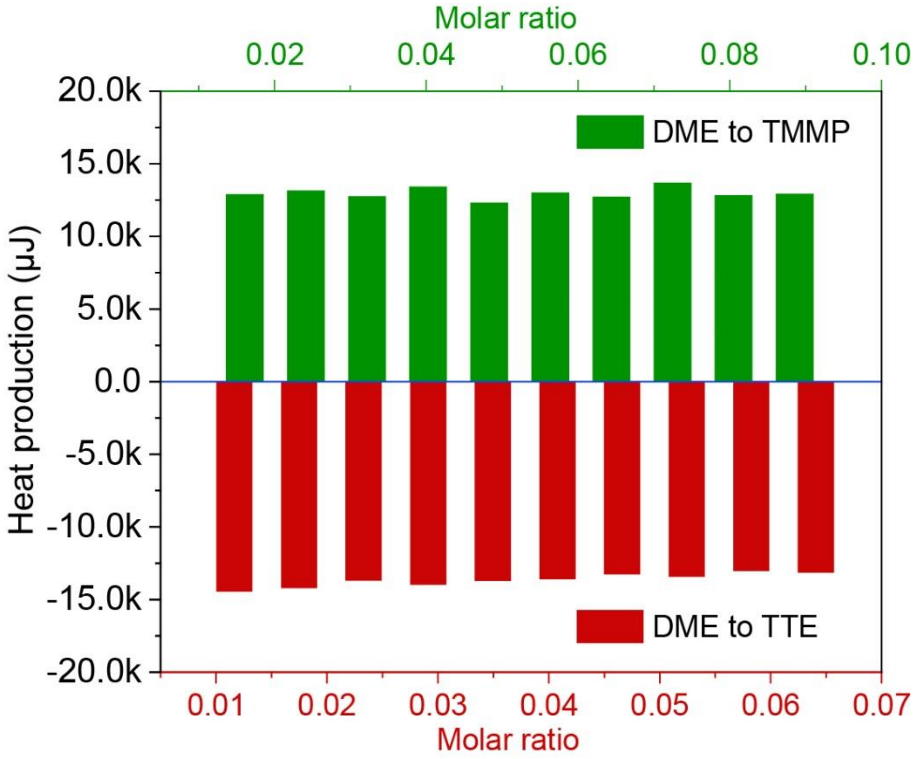

Figure S1. The corresponding heat production and absorption of Nano ITC data for mixing DME with TTE and DME with TMMP.

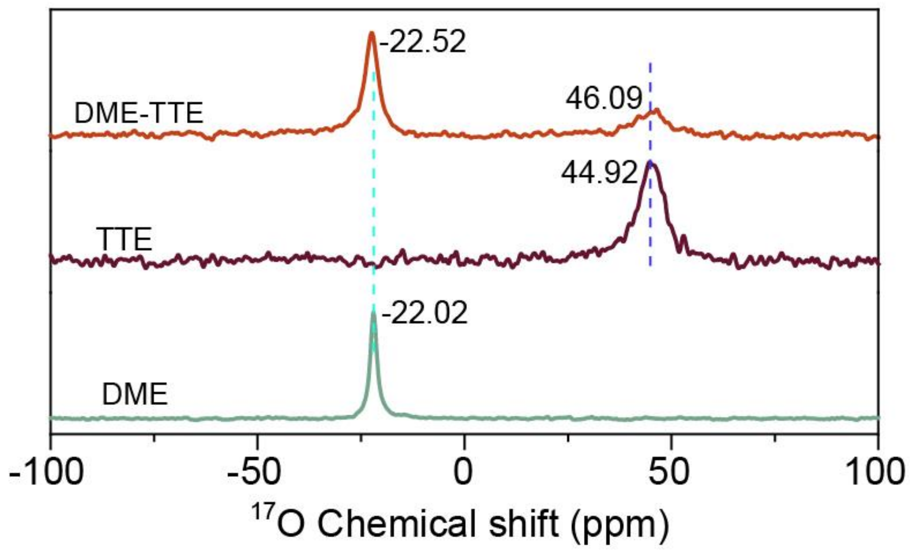

Figure S2. Solid-state ^{17}O NMR spectra of different solvents and mixtures.

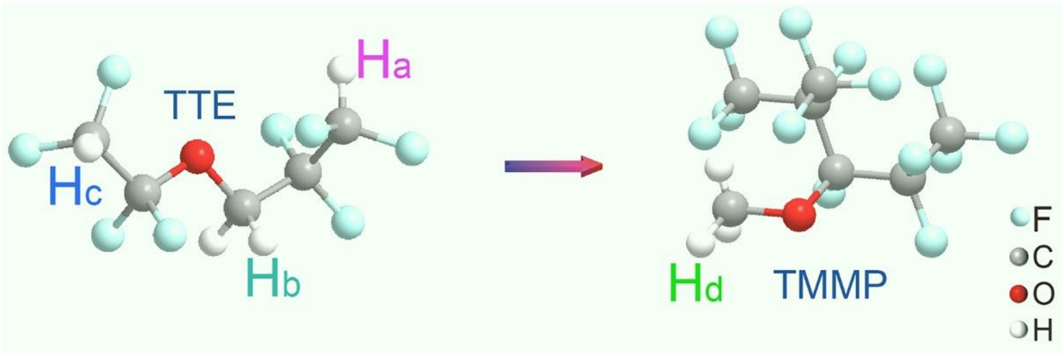

Figure S3. Molecular structures of TTE and TMMP.

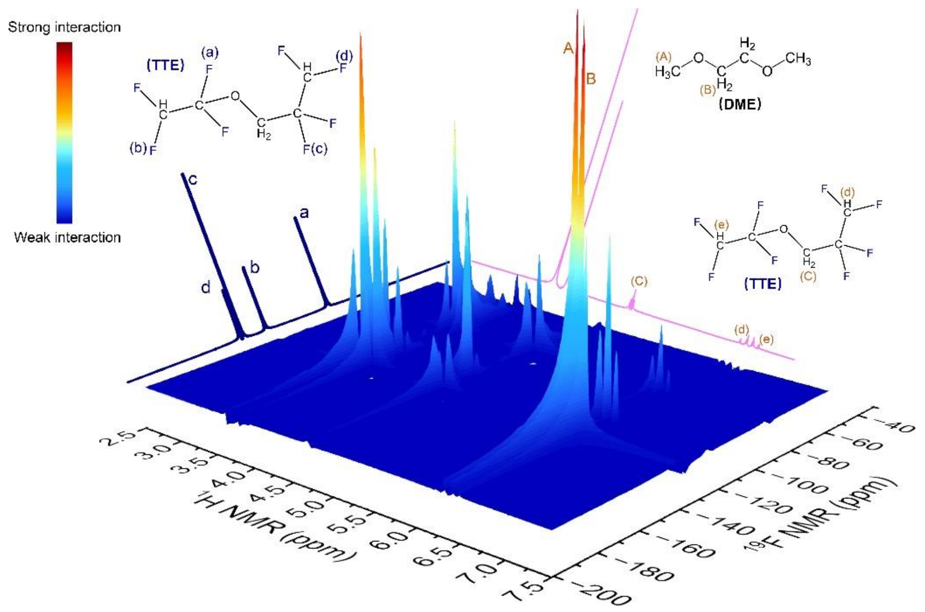

Figure S4. The ^{19}F-^{1}H HOESY NMR spectrum of DME-TTE mixture.

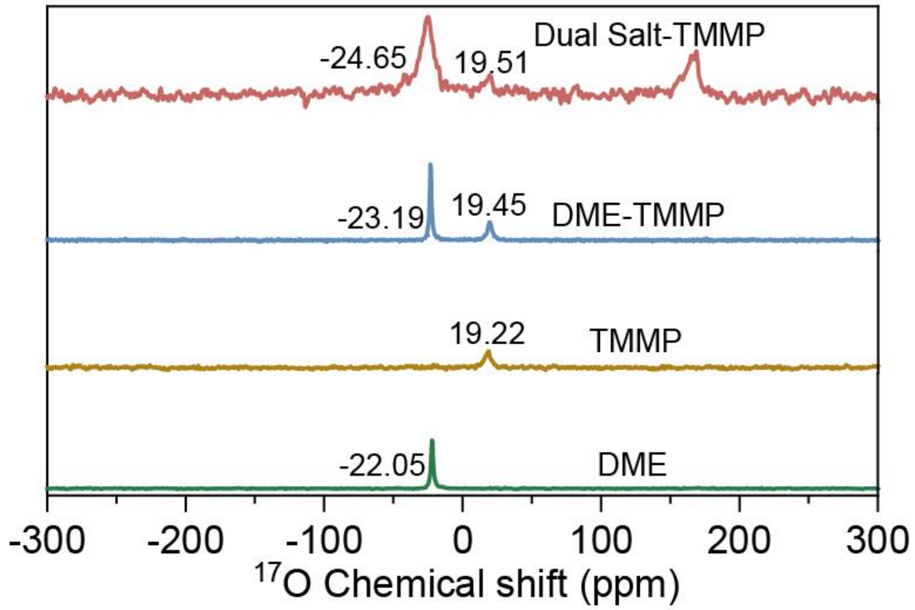

Figure S5. Solid-state ^{17}O NMR spectra of different solvents and mixtures.

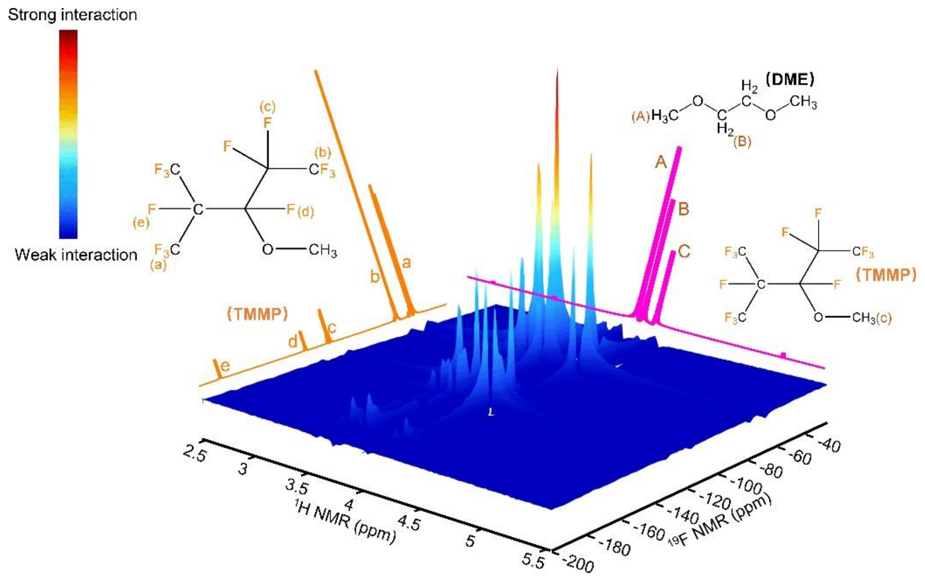

Figure S6. The ^{19}F-^{1}H HOESY NMR spectrum of DME-TMMP mixture.

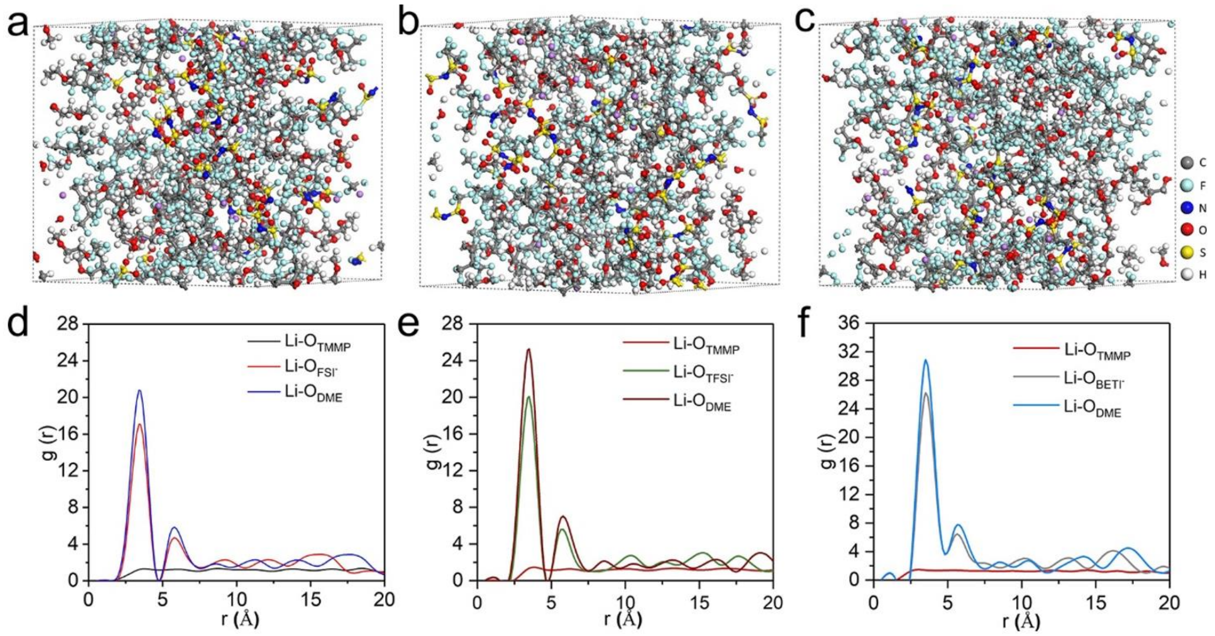

Figure S7. AIMD simulation snapshots of (a) LiFSI-DME-TMMP (1:2:2 by molar ratio), (b) LiTFSI-DME-TMMP (1:2:2 by molar ratio), and (c) LiBETI-DME-TMMP (1:2:2 by molar ratio). Radial distribution functions g(r) of (d) LiFSI-DME-TMMP (Li-O_{TMMP}, Li-O_{FSI}^{-}, Li-O_{DME}), (e) LiTFSI-DME-TMMP (Li-O_{TMMP}, Li-O_{TFSI}^{-}, Li-O_{DME}), and (f) LiBETI-DME-TMMP (Li-O_{TMMP}, Li-O_{BETI}^{-}, Li-O_{DME}).

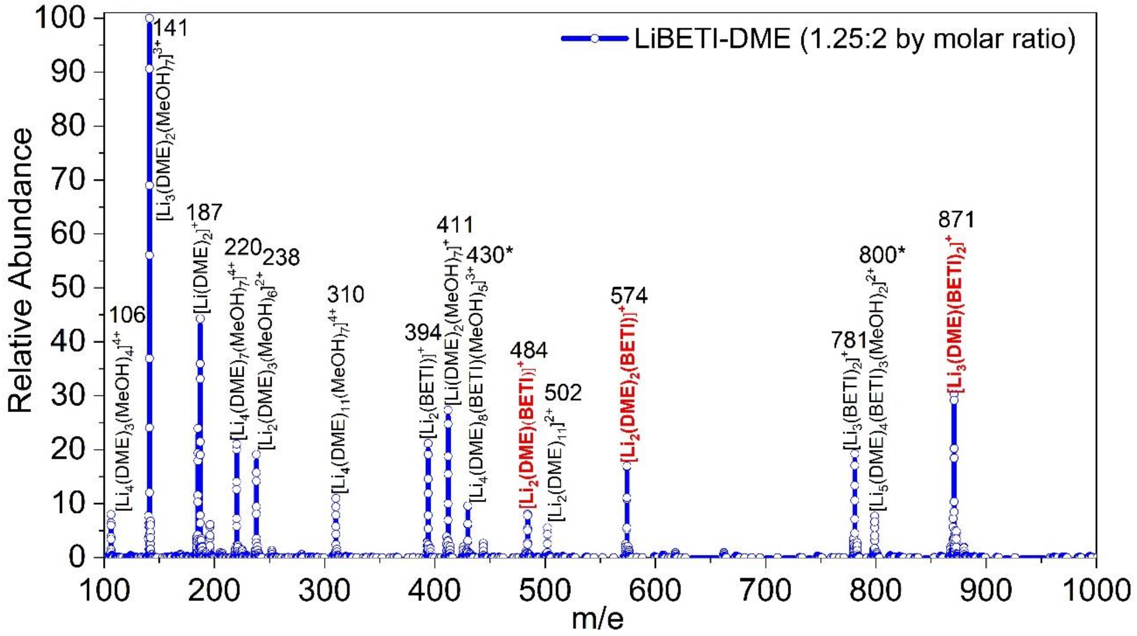

Figure S8. ESI-MS characterizations of LiBETI-DME mixture.

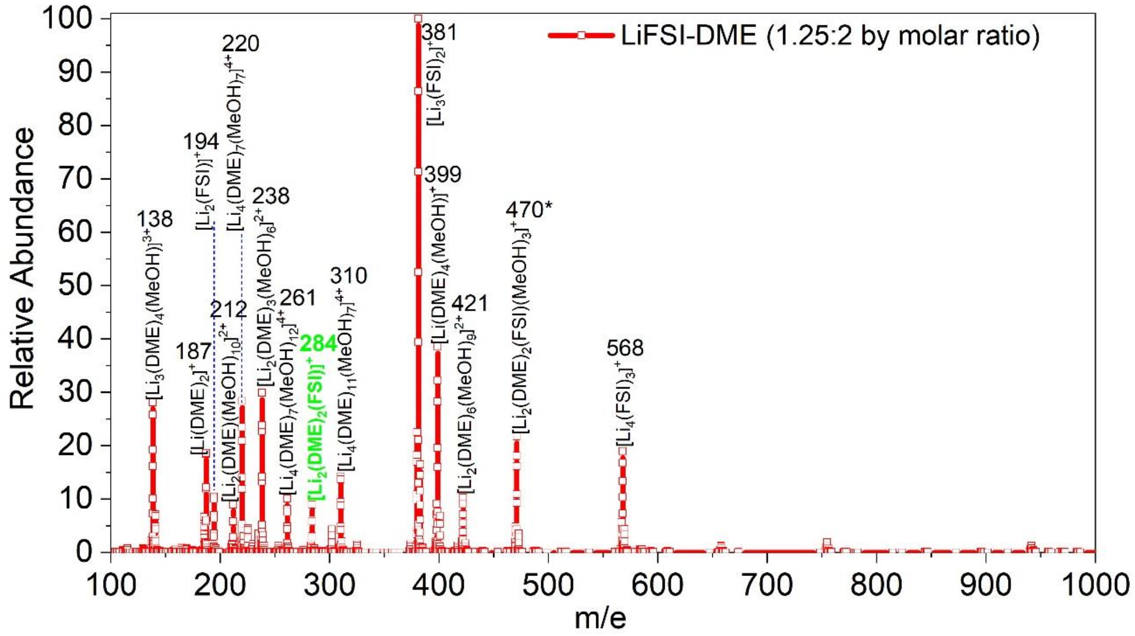

Figure S9. ESI-MS characterizations of the LiFSI-DME mixture.

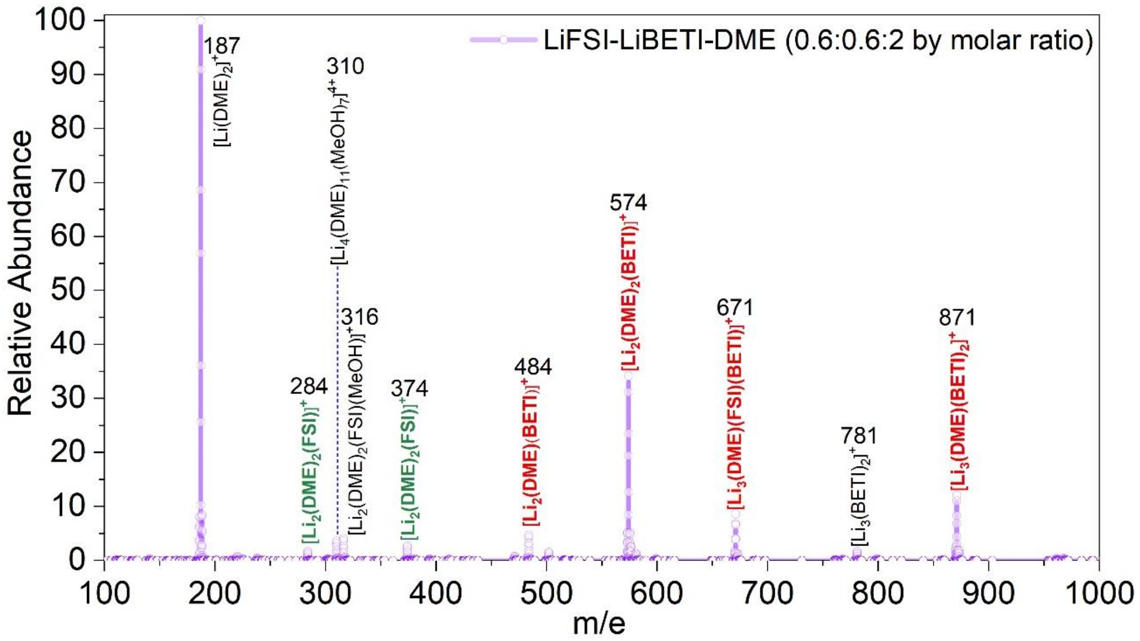

Figure S10. ESI-MS characterizations of LiFSI-LiBETI-DME mixture.

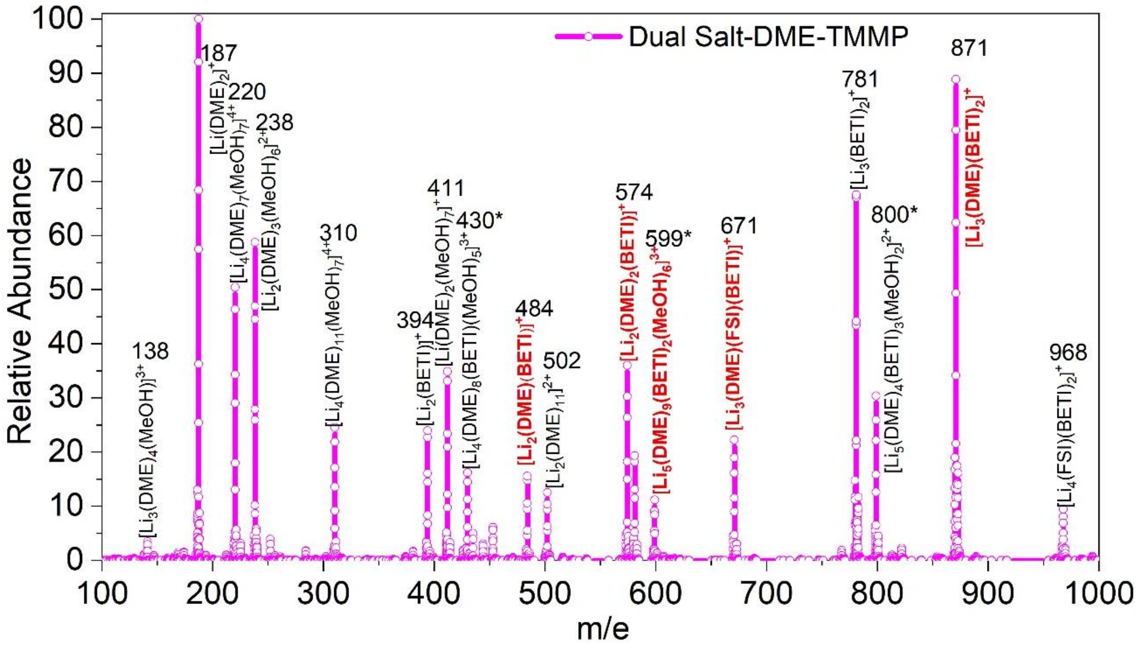

Figure S11. ESI-MS characterizations of Dual Salt-TMMP electrolyte.

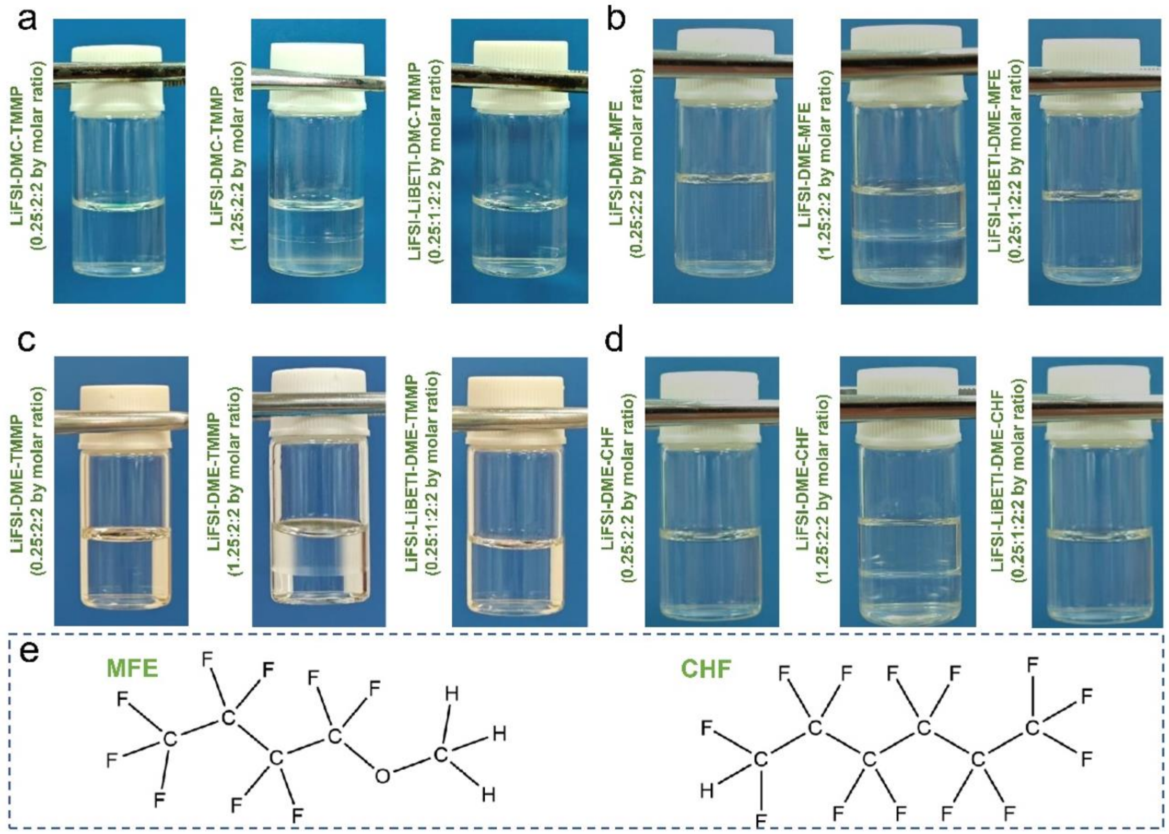

Figure S12. The digital photos of (a) LiFSI-DMC-TMMP (0.25:2:2 by molar ratio), LiFSI-DMC-TMMP (1.25:2:2 by molar ratio), and LiFSI-LiBETI-DMC-TMMP (0.25:1:2:2 by molar ratio). (b) LiFSI-DME-MFE (0.25:2:2 by molar ratio), LiFSI-DME-MFE (1.25:2:2 by molar ratio), and LiFSI-LiBETI-DME-MFE (0.25:1:2:2 by molar ratio). (c) LiFSI-DME-TMMP (0.25:2:2 by molar ratio), LiFSI-DME-TMMP (1.25:2:2 by molar ratio), and LiFSI-LiBETI-DME-TMMP (0.25:1:2:2 by molar ratio). (d) LiFSI-DME-CHF (0.25:2:2 by molar ratio), LiFSI-DME-CHF (1.25:2:2 by molar ratio), and LiFSI-LiBETI-DME-CHF (0.25:1:2:2 by molar ratio). e) Molecular structure of MFE and CHF.

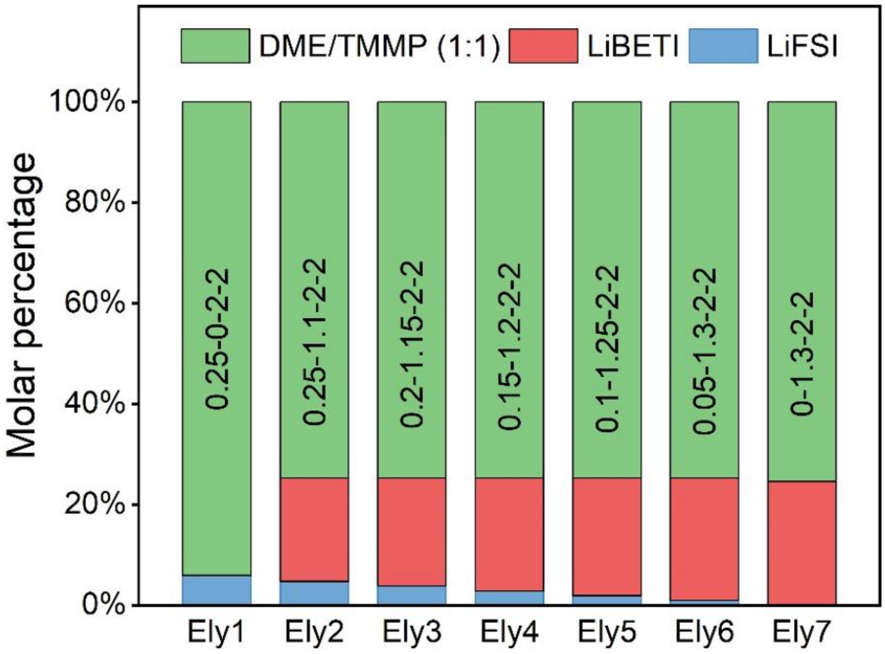

Figure S13. Maximum solubility of LiBETI with different LiFSI concentrations in DME/TMMP (1:1) mixture.

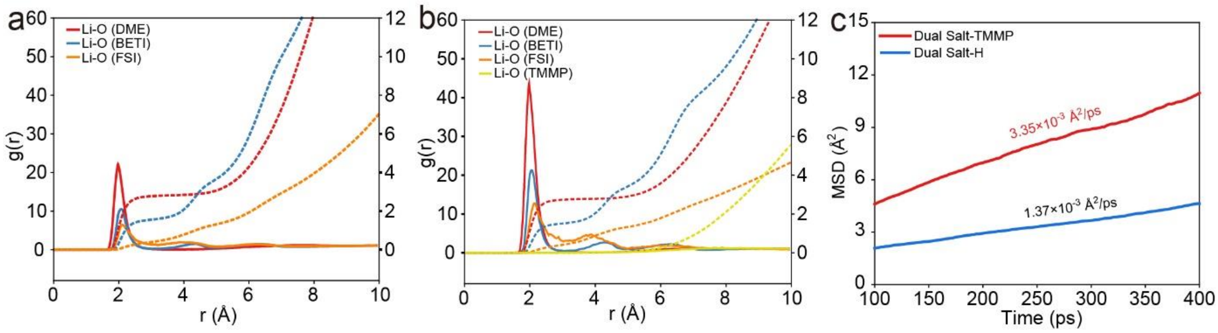

Figure S14. (a) Calculated radial distribution functions ($g(r)$, solid lines) and coordination numbers (CN, dashed line) of O, F, and F around Li^+ as a function of distance (r) in (a) Dual Salt-DME-H, and (b) Dual Salt-DME-TMMP. (c) MSD of Li^+ ions in Dual Salt-H and Dual Salt-TMMP electrolytes.

MD simulations were employed to examine the solvation structures of various electrolytes. The radial distribution functions (RDFs) from these simulations show a sharp peak at approximately 1.98 Å for the Li-O (DME) bond in both systems (**Figures S14a-S14b**), indicating that DME molecules form the first coordination shell around Li^+ ions. In Figure S14a, the high salt-to-solvent ratio in Dual Salt-H results in a large number of DME molecules coordinating with Li^+ ions, leaving only a small amount of free DME. Additional, Dual Salt-TMMP ensures that nearly all solvent molecules coordinate with Li^+ ions and salt anions, while diluent molecules are mostly excluded from the inner solvation shell due to their poor ion-solvating capability (**Figure S14b**). The near-zero coordination number for the Li-O (TMMP) pair indicates that TMMP has minimal impact on the solvation structure in Dual Salt-TMMP. Consequently, the solvation structure of Dual Salt-TMMP closely resembles that of Dual Salt-H, preserving its advantageous reactivity characteristics.

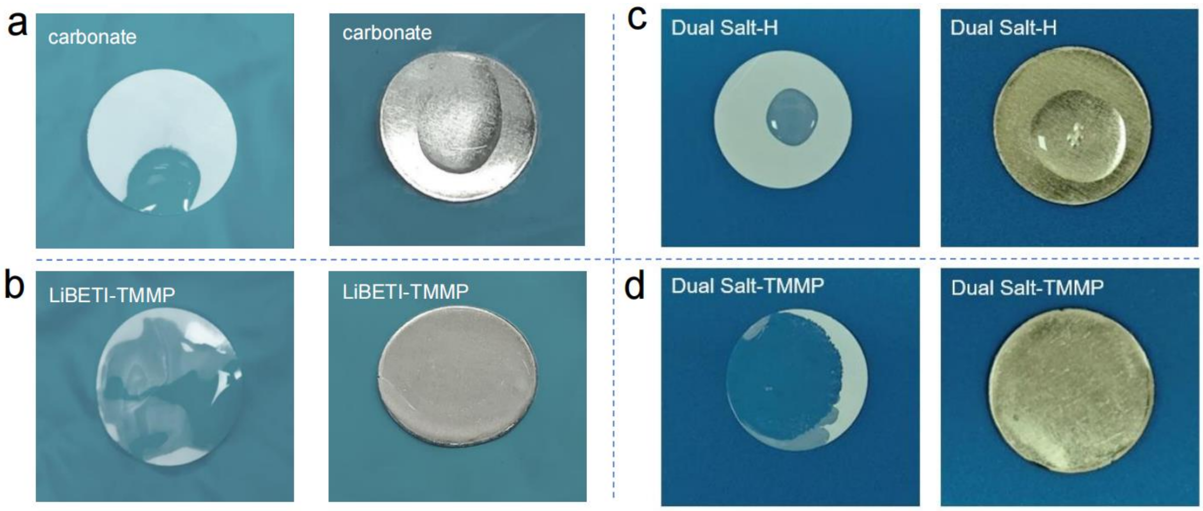

Figure 15. Comparison of electrolyte wettability on the separator and Li anode: (a) for carbonate, (b) for LiBETI-TMMP, and (c) for Dual Salt-H, and (d) for Dual Salt-TMMP.

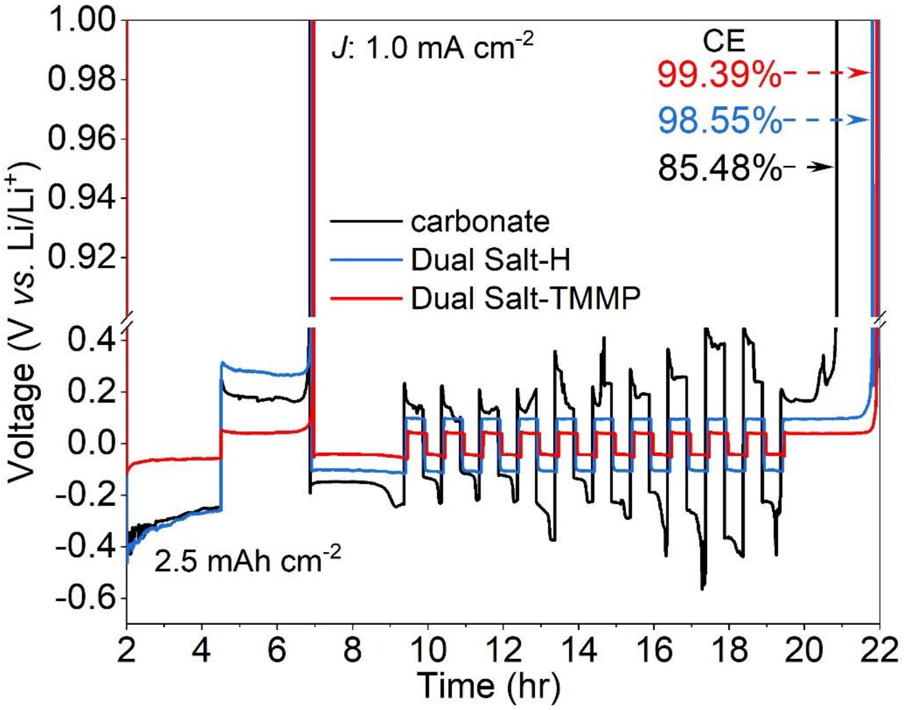

Figure S16. Measurements of Li CE in different electrolytes at current densities (denoted as *J*) of 1 mA cm^{-2} using Li||Cu batteries.

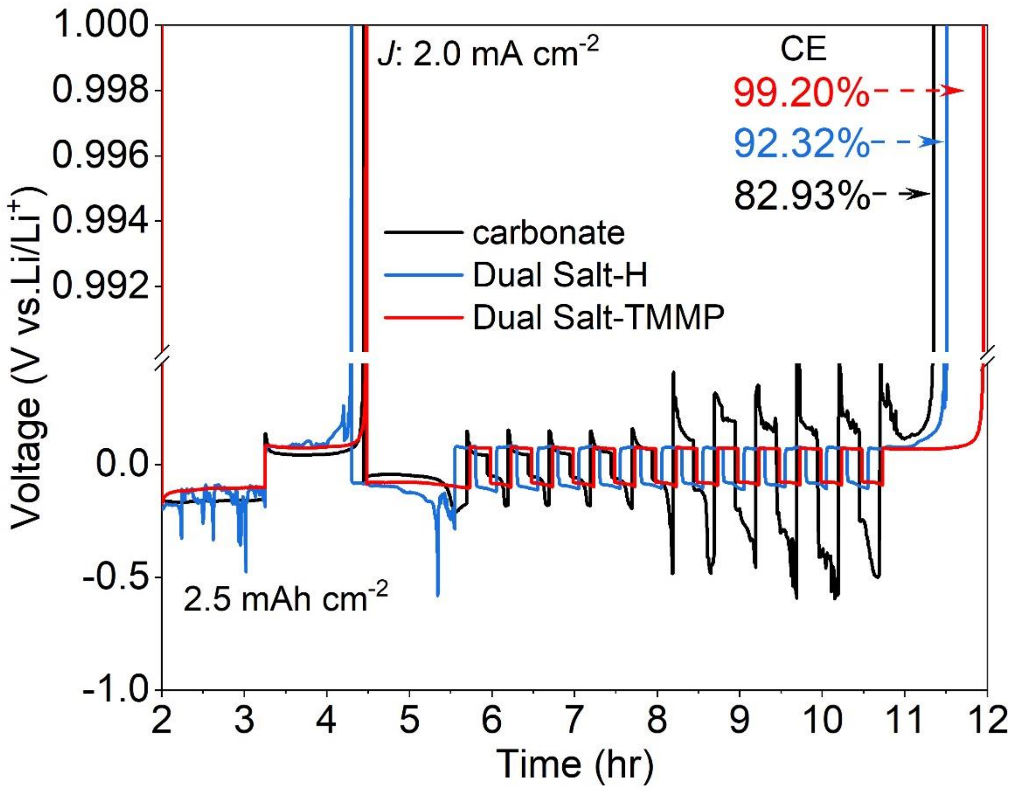

Figure S17. Measurements of CE for Li metal anodes in different electrolytes at current densities of 2 mA cm^{-2} using Li||Cu batteries.

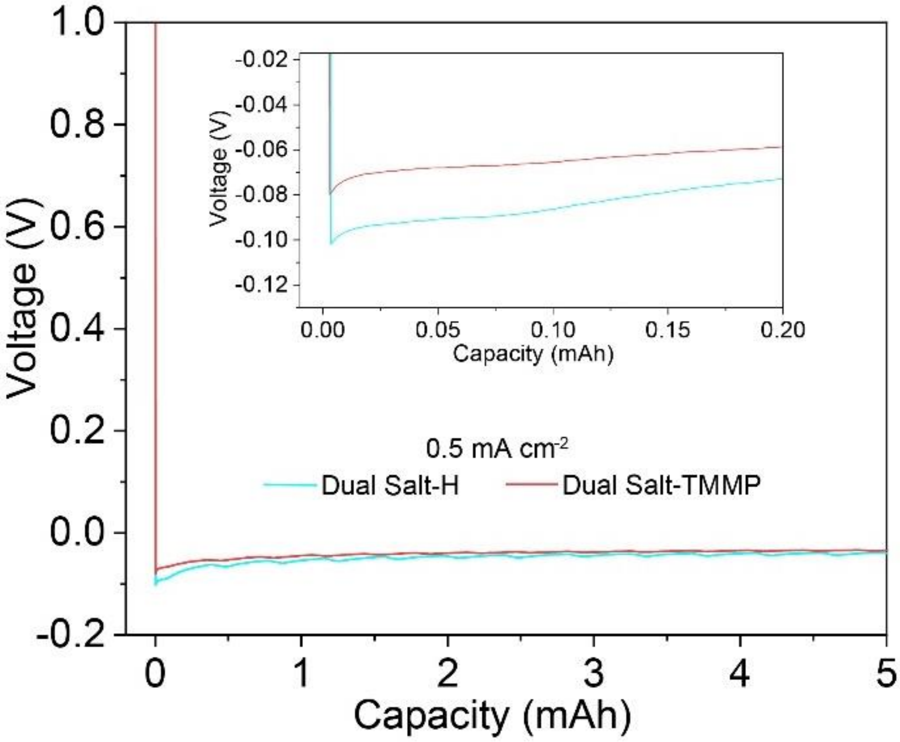

Figure S18. The initial capacity-voltage of Li||Cu cells at 0.5 mA cm^{-2} with a fixed capacity of 2.5 mAh cm^{-2}.

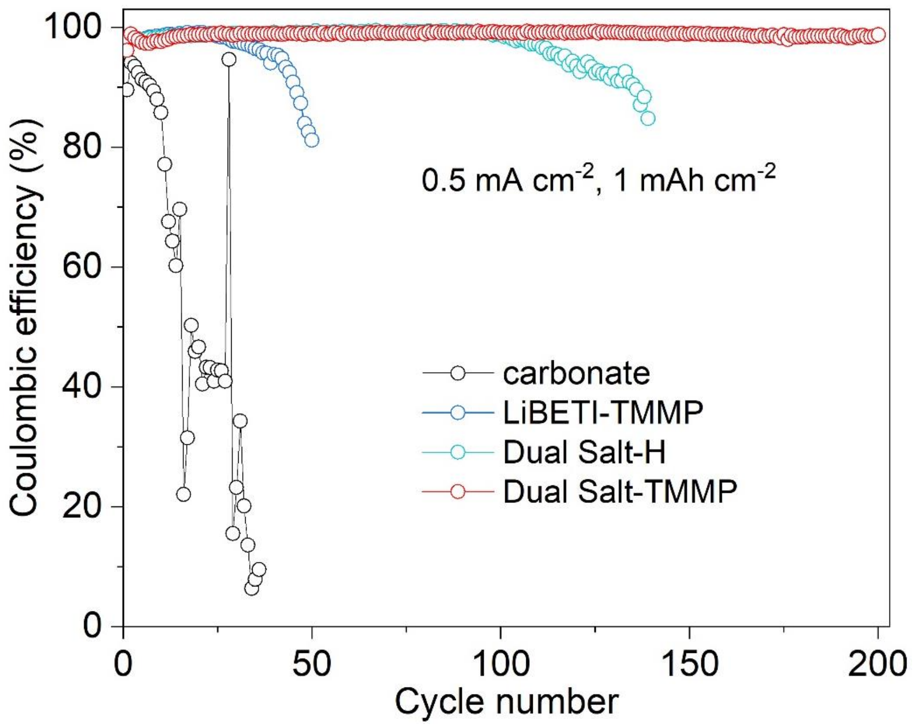

Figure S19. Li-metal CEs with cycling in different electrolytes measured in Li||Cu cells by repeated plating and stripping processes.

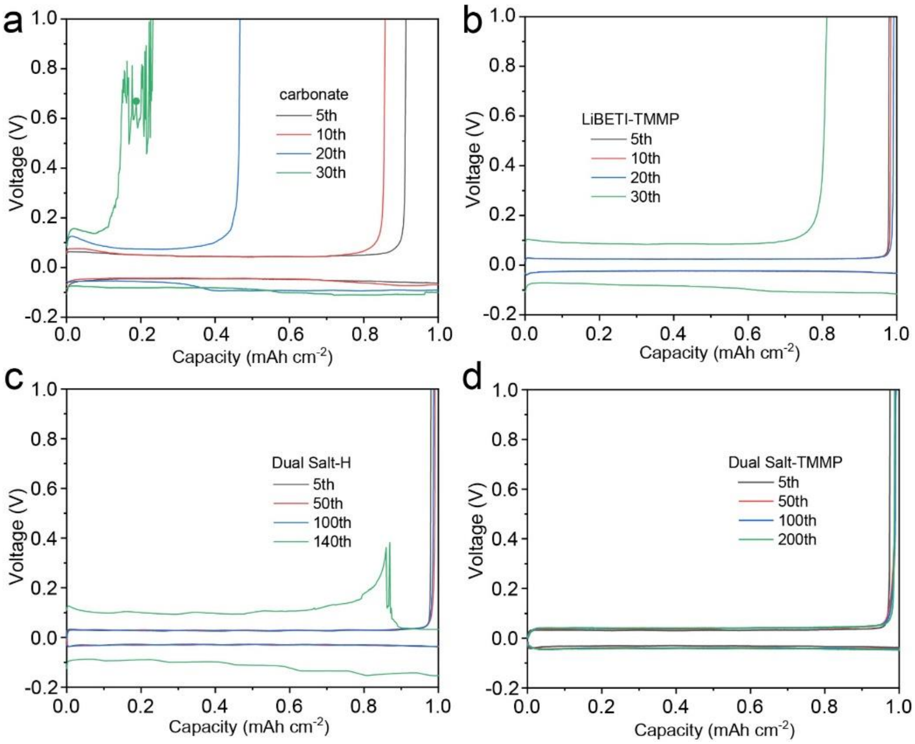

Figure S20. Voltage profiles of Li plating and stripping processes at selected cycles in different electrolytes: (a) carbonate, (C) LiBETI-TMMP, (c) Dual Salt-H, and (d) Dual Salt-TMMP.

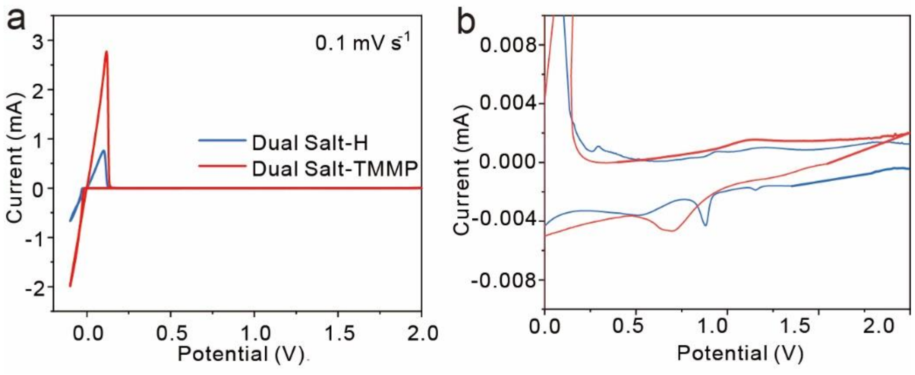

Figure S21. CV curves of Li||Cu cells using different electrolytes. The voltage range is -0.1 V to 2.0 V, and the scanning rate is 0.1 mV s^{-1}.

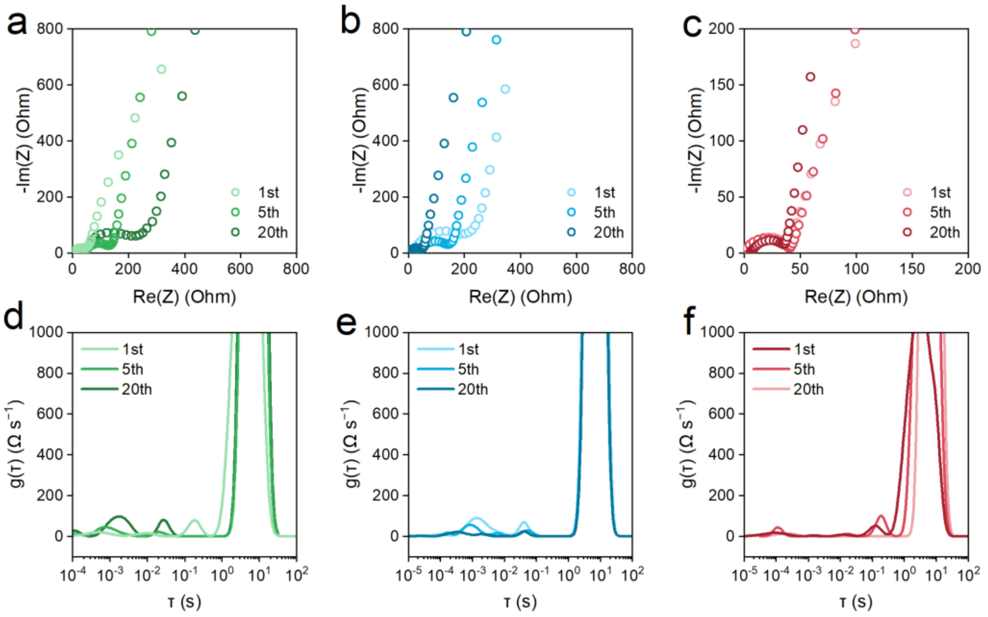

Figure S22. EIS and corresponding DRT analysis of Li||Cu cells using different electrolytes: (a, d) carbonate, (b, e) Dual Salt-H, and (c, f) Dual Salt-TMMP.

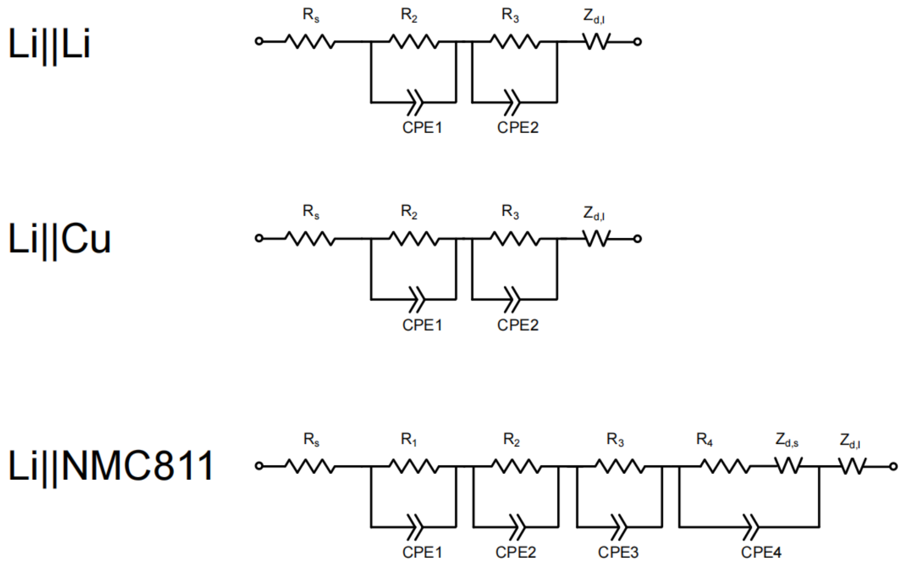

Figure S23. The equivalent circuit employed for fitting the EIS of Li||Cu, Li||Li, and Li||NCM811 cells.

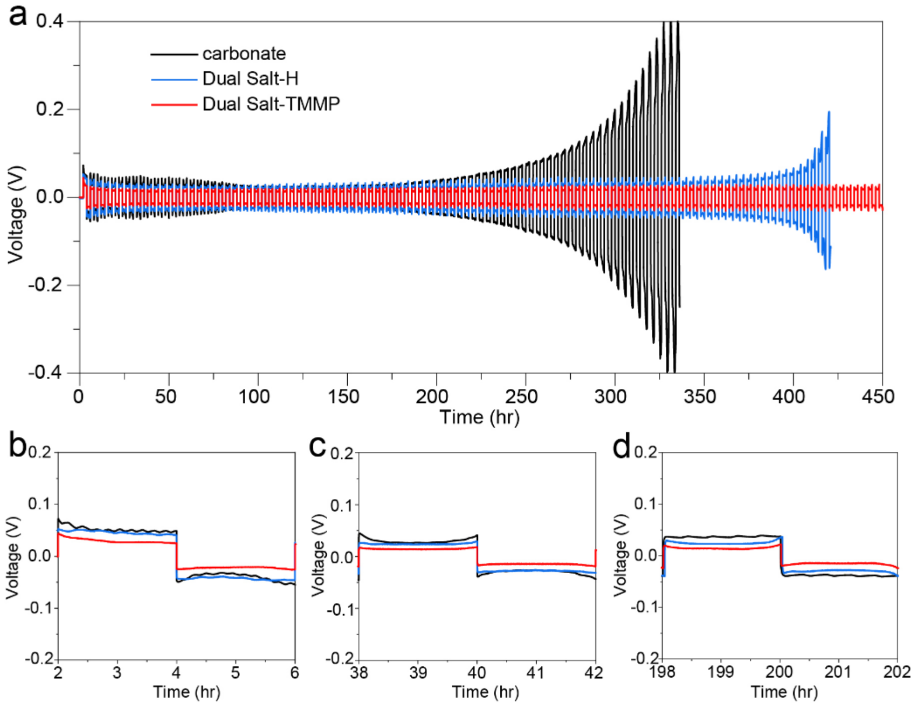

Figure S24. Voltage profiles of Li||Li symmetric cells using various electrolytes: (a) full cycling process; (b-d) magnified views of the 1st, 10th, and 50th cycles, respectively.

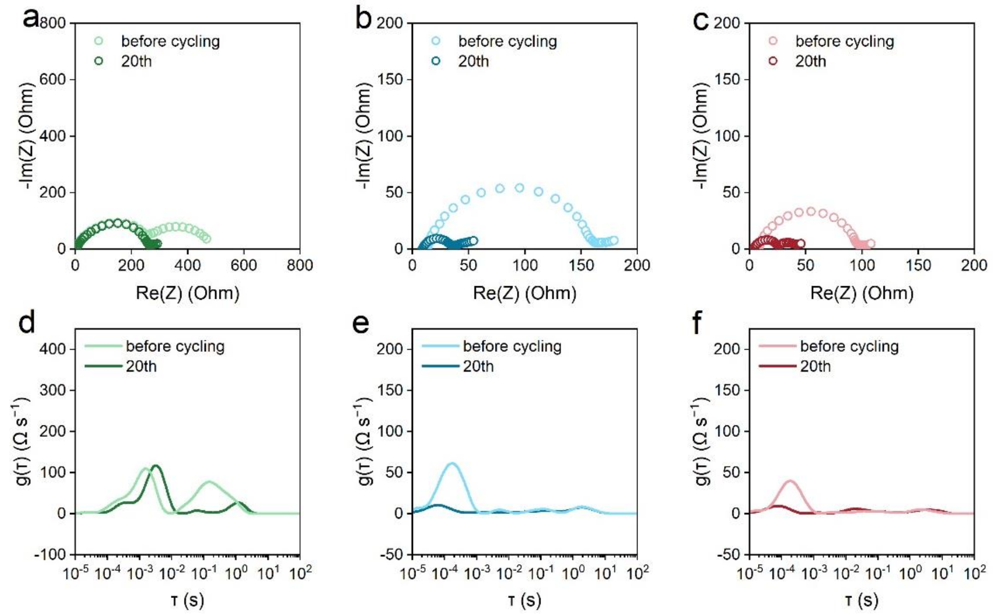

Figure S25. EIS and corresponding DRT analysis of Li||Li cells using different electrolytes: (a, d) carbonate, (b, e) Dual Salt-H, and (c, f) Dual Salt-TMMP.

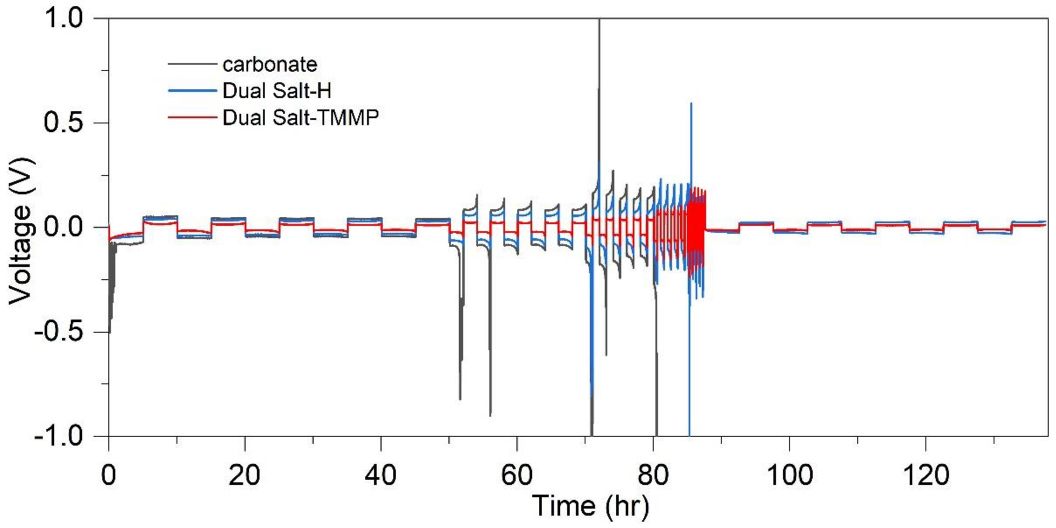

Figure S26. Rate performance of Li||Li symmetric batteries at a current of 0.2, 0.5, 1, 2, , 4, and 0.2 mA cm^{-2} and a capacity of 1 mAh cm^{-2}.

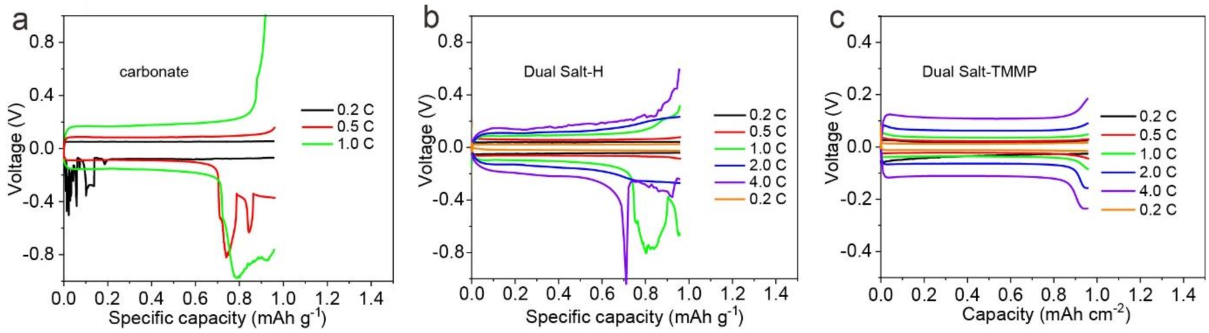

Figure S27. Capacity-voltage curves corresponding to the rate performance of Li||Li symmetric batteries with different electrolytes: (a) carbonate; (b) Dual Salt-H; (c) Dual Salt-TMMP.

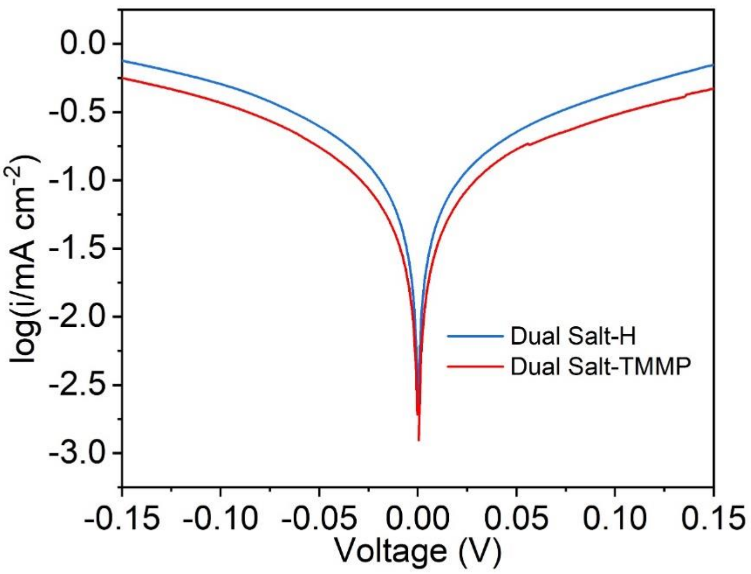

Figure S28. Exchange current test of Li||Li cells with Dual Salt-H and Dual Salt-TMMP electrolytes.

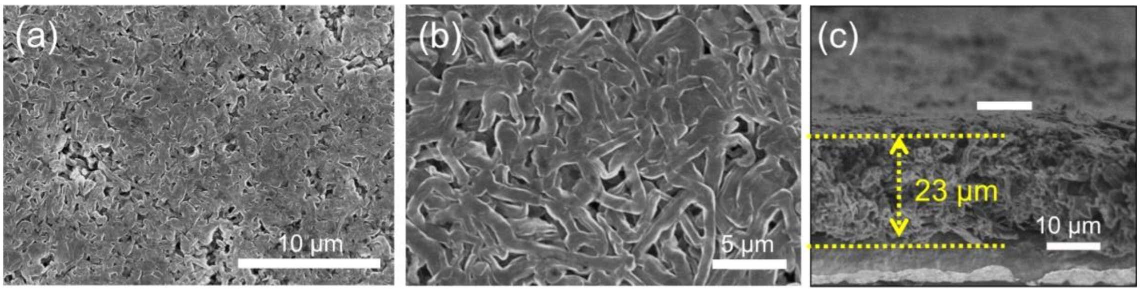

Figure S29. Morphology of Li metal plating on Cu foil. Top and cross-sectional SEM images of Li deposits obtained in the carbonate by plating 2 mAh cm^{-2} of Li on Cu substrate at current densities of 0.5 mA cm^{-2}.

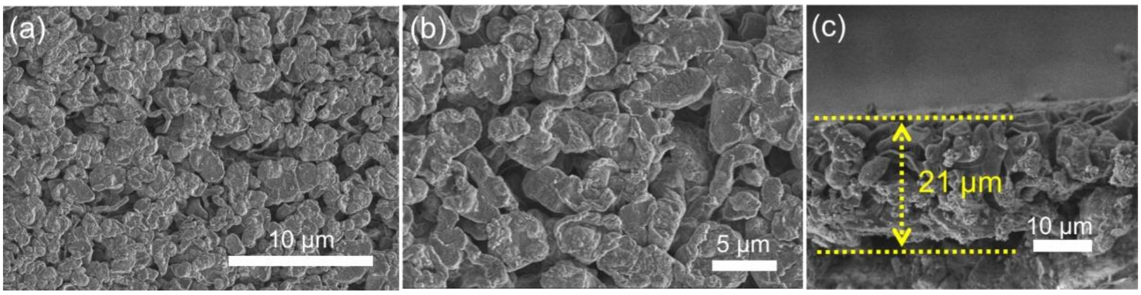

Figure S30. Morphology of Li metal plating on Cu foil. Top and cross-sectional SEM images of Li deposits obtained in the Dual Salt-H by plating 2 mAh cm^{-2} of Li on Cu substrate at current densities of 0.5 mA cm^{-2}.

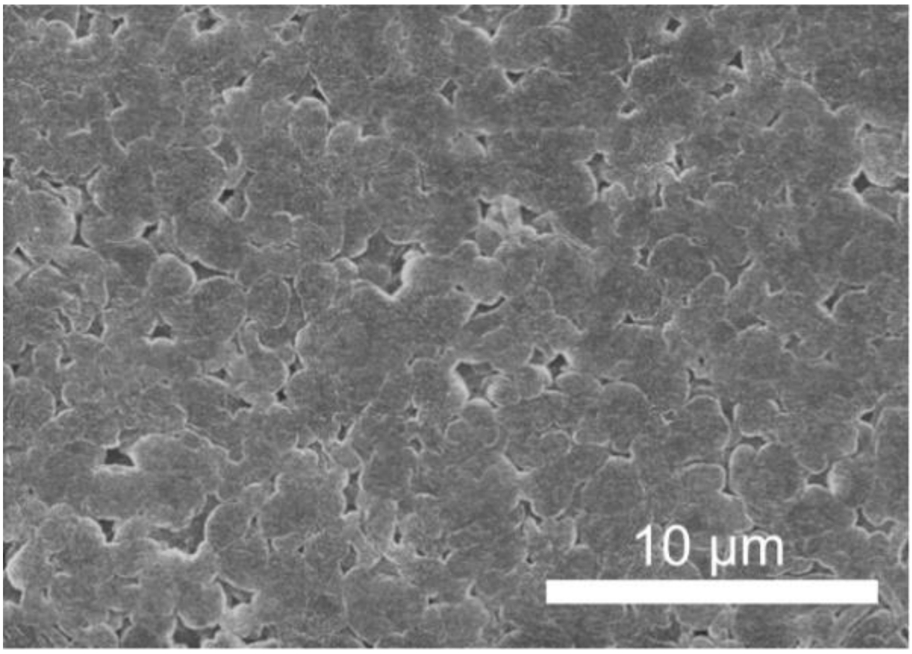

Figure S31. Morphology of Li metal plating on Cu foil. Top and cross-sectional SEM images of Li deposits obtained in the Dual Salt-TMMP by plating 2 mAh cm^{-2} of Li on Cu substrate at current densities of 0.5 mA cm^{-2}.

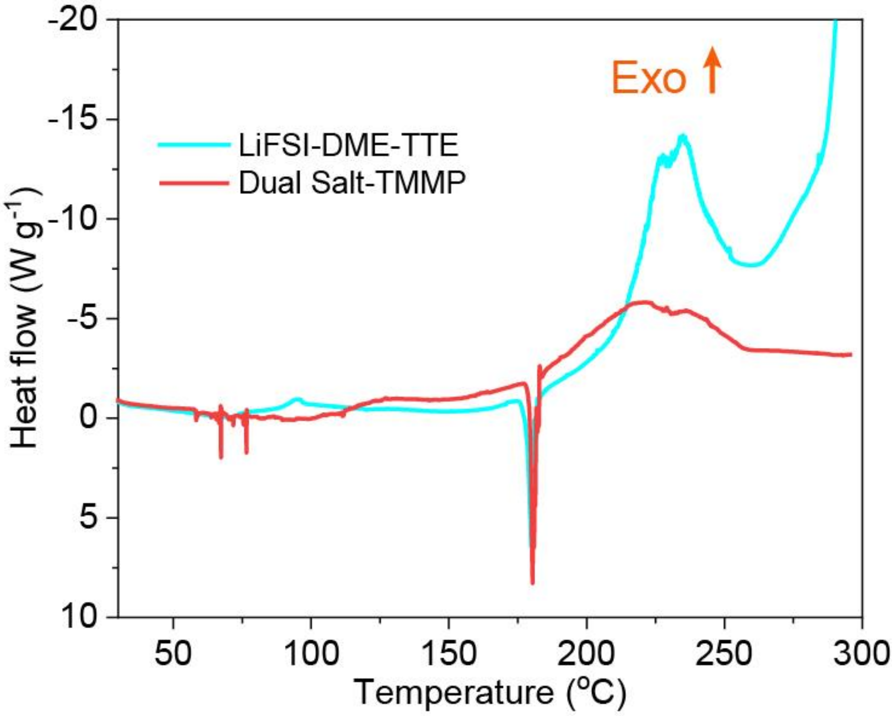

Figure S32. DSC traces for LiFSI-TTE, and Dual Salt-TMMP electrolytes with deposited Li metal.

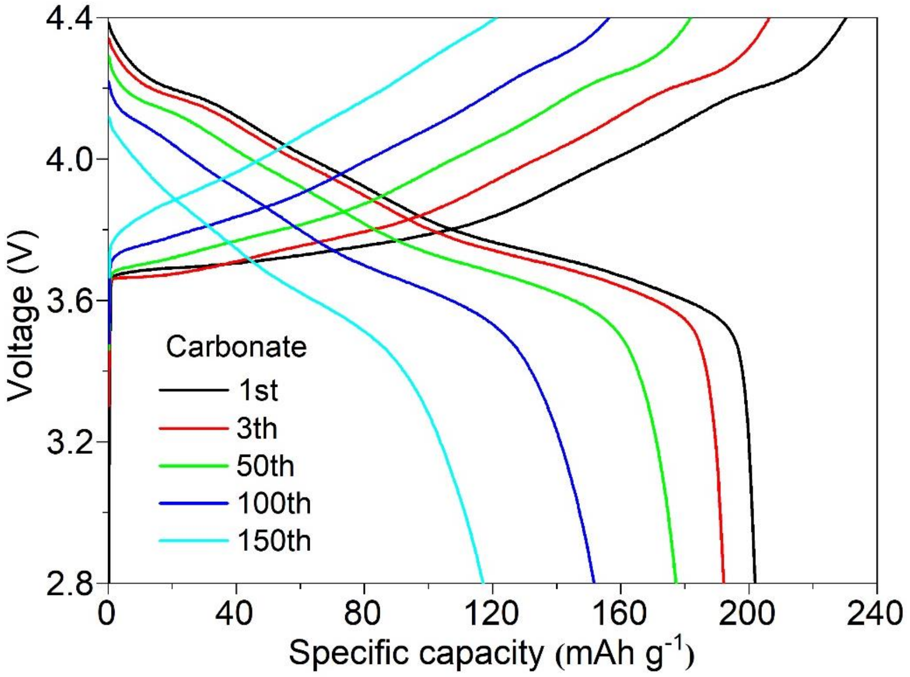

Figure S33. Voltage profiles of Li||NCM811 in carbonate electrolyte.

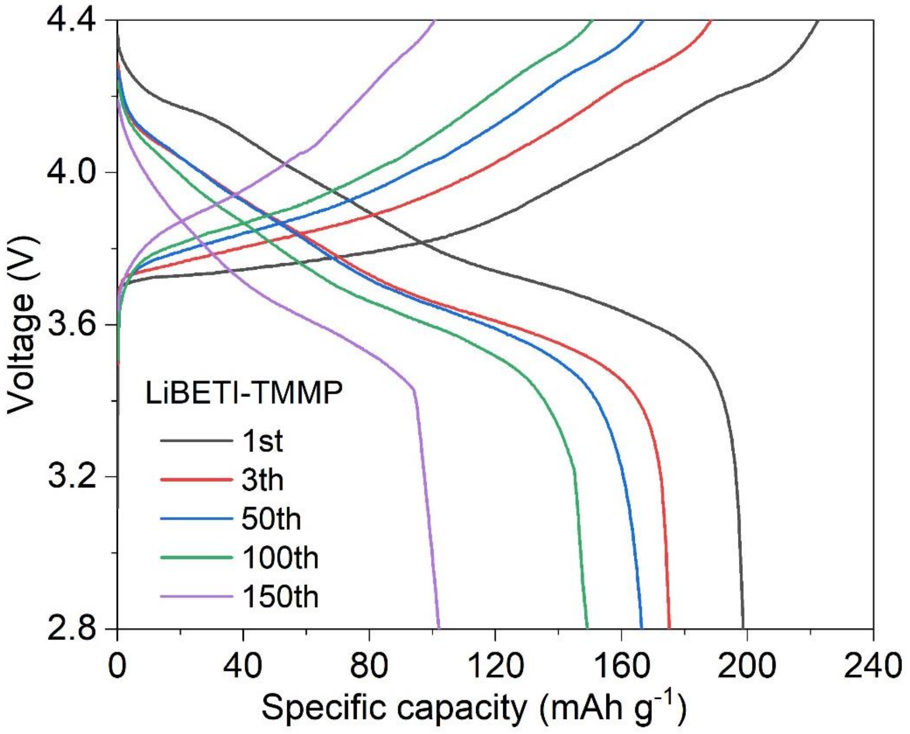

Figure S34. Voltage profiles of Li||NCM811 in LiBETI-TMMP electrolyte.

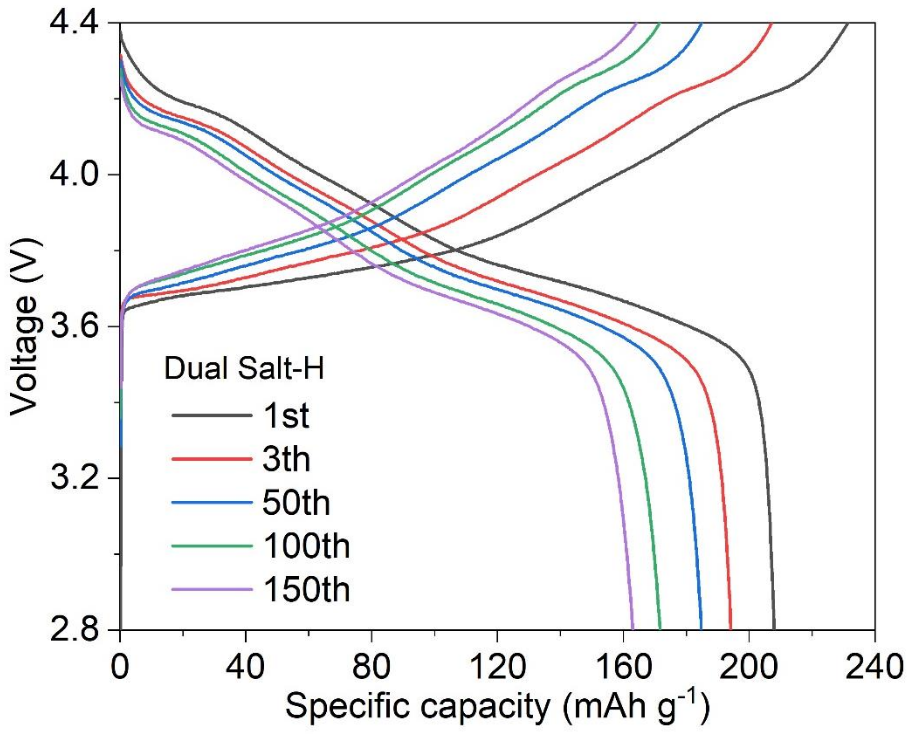

Figure S35. Voltage profiles of Li||NCM811 in Dual Salt-H.

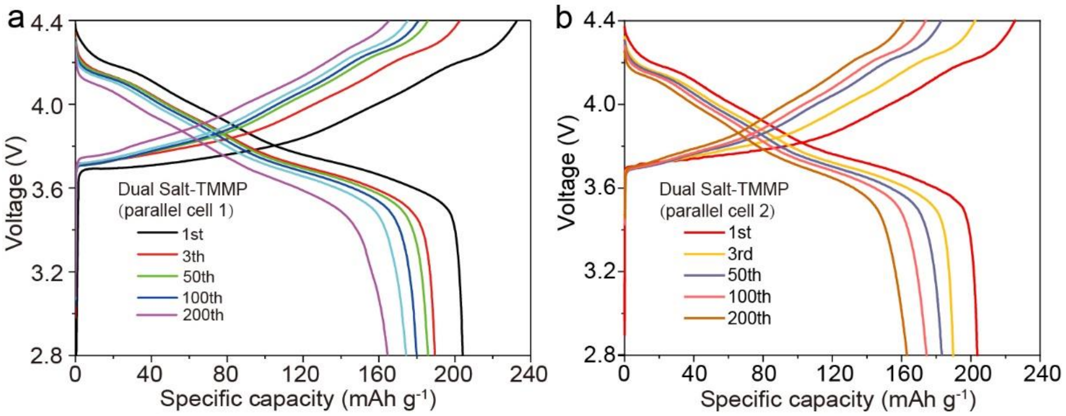

Figure S36. Voltage profiles of two parallel Li||NCM811 cells in the Dual Salt-TMMP electrolyte: (a) parallel cell 1, (b) parallel cell 2.

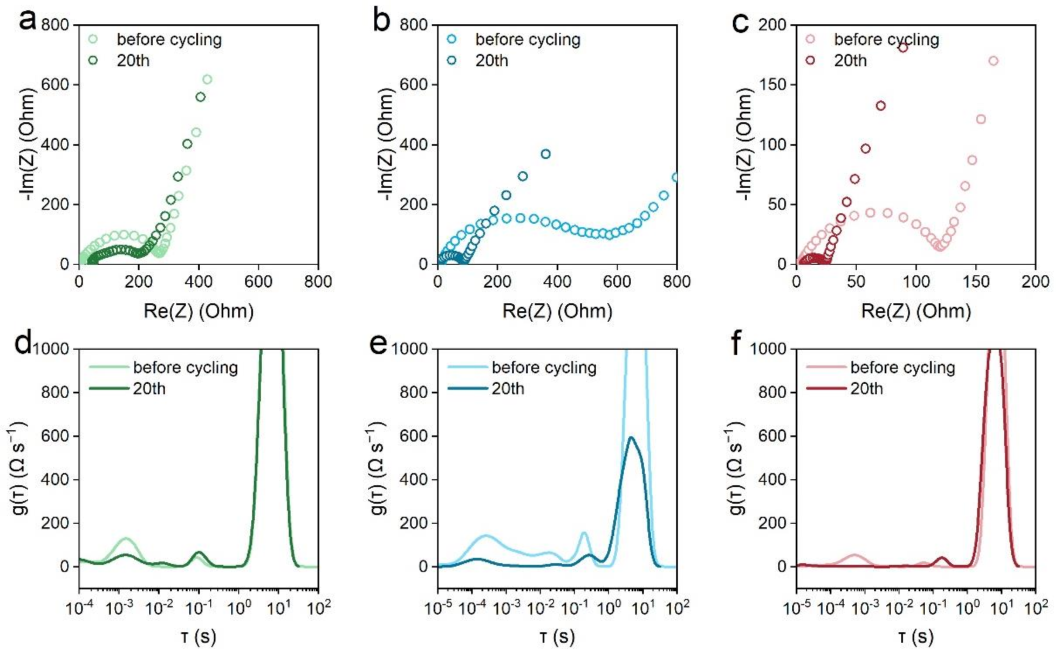

Figure S37. EIS and corresponding DRT analysis of Li||NCM811 cells using different electrolytes: (a, d) carbonate, (b, e) Dual Salt-H, and (c, f) Dual Salt-TMMP.

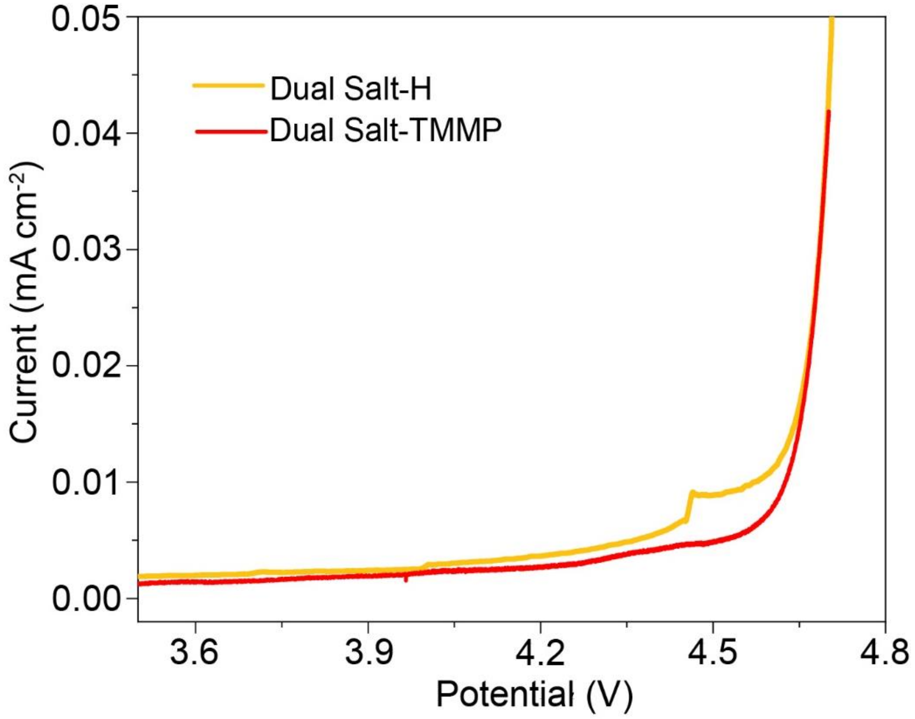

Figure S38. LSV curves of Dual Salt-H and Dual Salt-TMMP electrolytes on Super P-PVDF electrodes. (Super P areal loading: 0.6 mg cm^{-2}, scan rate: 0.1 mV s^{-1}).

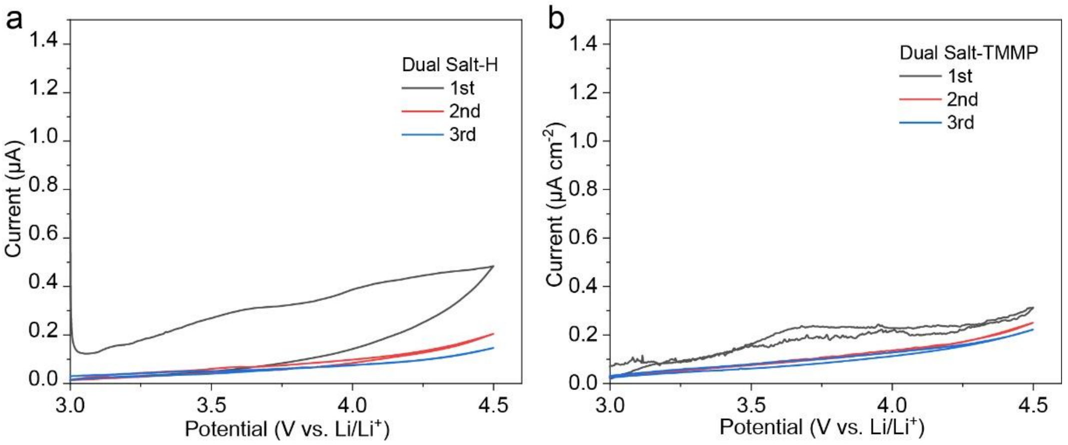

Figure S39. CV curves of Li||Al cells using different electrolytes: (a) Dual Salt-H and (b) Dual Salt-TMMP. The voltage range is 3.0 V to 4.5 V, and the scanning rate is 0.1 mV s^{-1}.

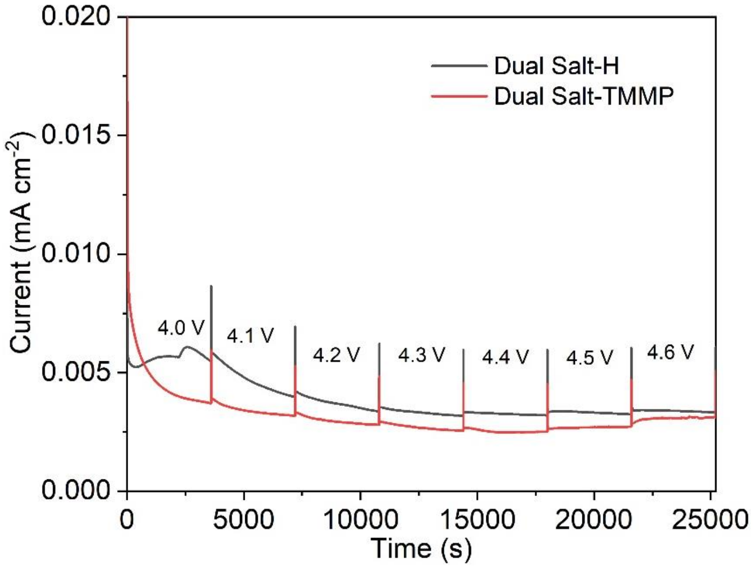

Figure S40. Constant-potential measurements of Li/Al cells in Dual Salt-H and Dual Salt-TMMP electrolytes. The potential was increased stepwise from 4.0 V to 4.6 V (vs. Li/Li^+), each step held for 3600 s, to assess the anodic stability of the electrolytes.

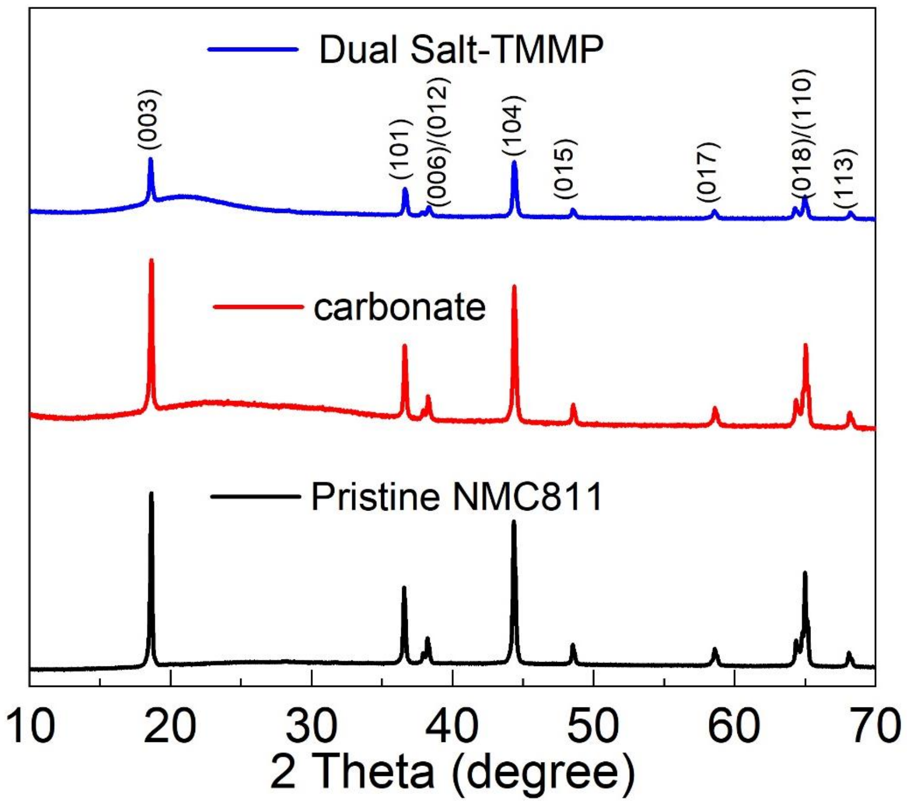

Figure S41. XRD patterns of the pristine NCM811 cathode and the cycled NCM811 cathodes in carbonate and Dual Salt-TMMP electrolytes after 200 cycles.

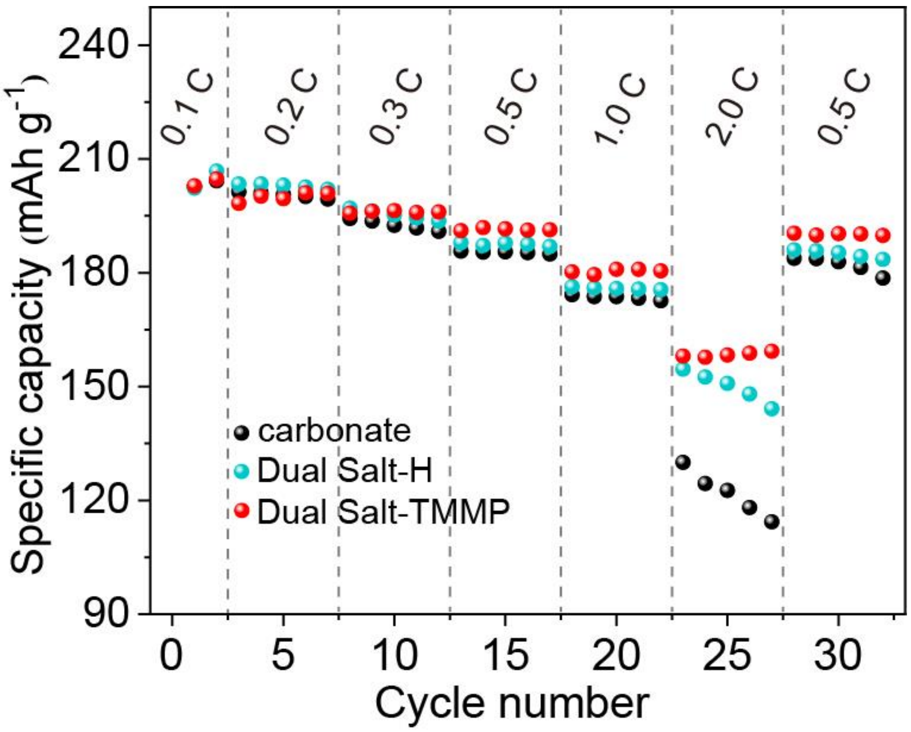

Figure S42. Specific capacity of the Li||NCM811 batteries under various discharge rates at 30°C.

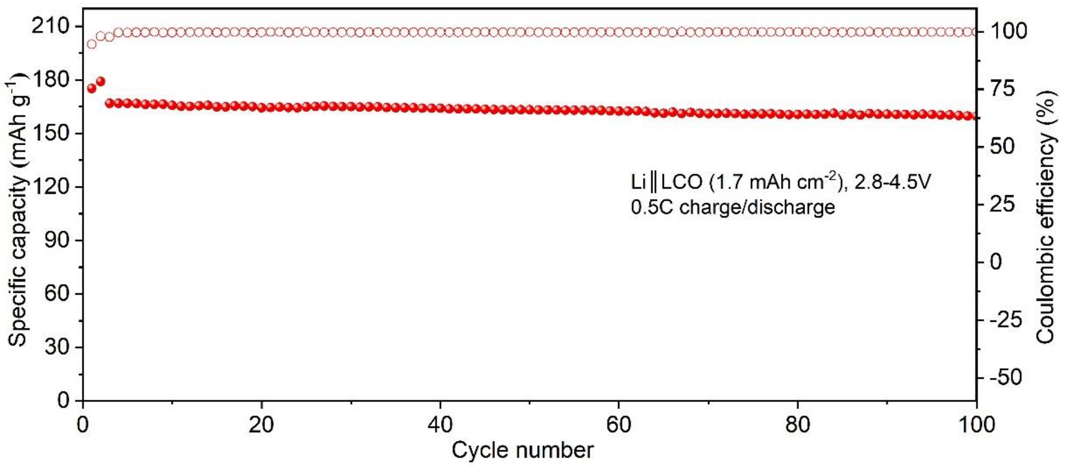

Figure S43. Cycling performance of Li||$LiCoO_2$ (LCO) battery with charged/discharged rate at 0.5C between 2.8 and 4.5 V after two formation cycles in Dual Salt-TMMP at C/10. 1C equals 160 mA g^{-1} of LCO.

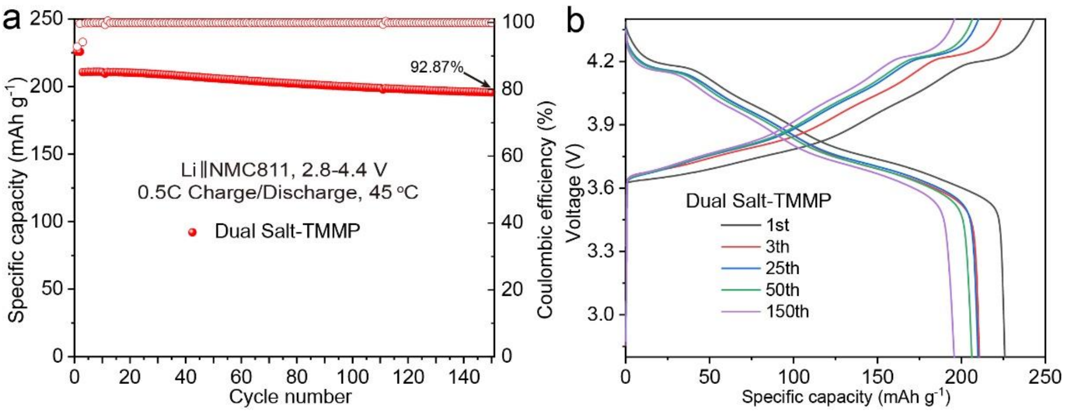

Figure S44. Cycling performance of Li||NCM811 battery at a charge/discharge rate of 0.5C, between 2.8 and 4.4 V, after two formation cycles at C/10 in Dual Salt-TMMP at 45 °C.

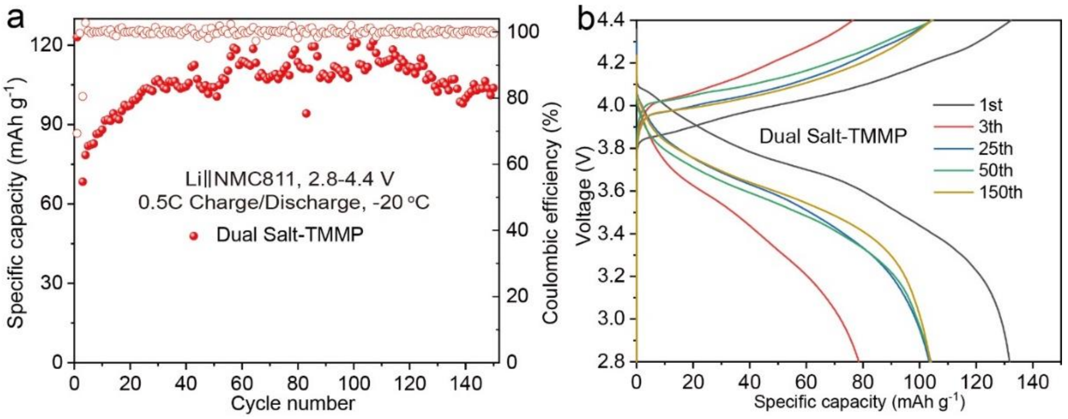

Figure S45. (a) Cycling performance of the 4.4 V Li||NCM811 cell using the Dual Salt-TMMP electrolyte at -20 °C with a charge/discharge rate of 0.5 C. (b) Charge and discharge profiles of the Dual Salt-TMMP electrolyte during cycling.

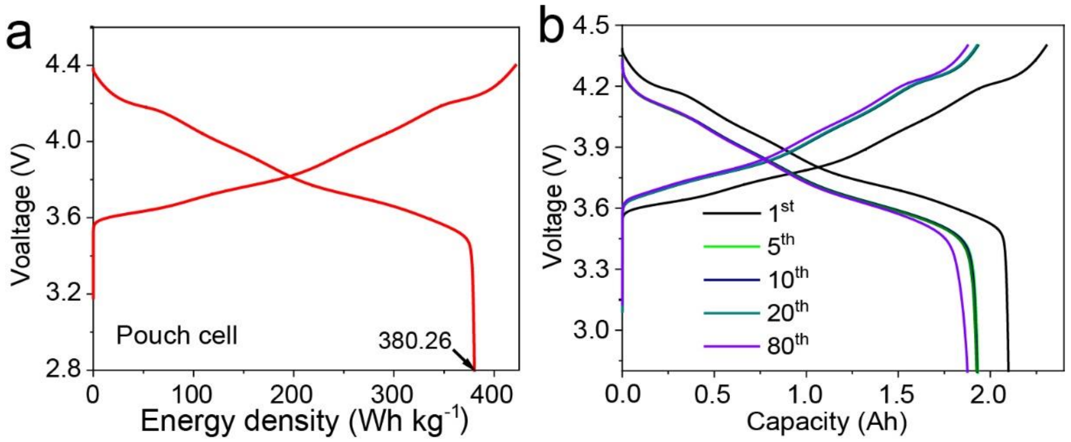

Figure S46. (a) Initial charge-discharge curve at C/20 of the Li‖NCM811 pouch cell. (b) Voltage profiles of the Li‖NCM811 pouch cell during cycling.

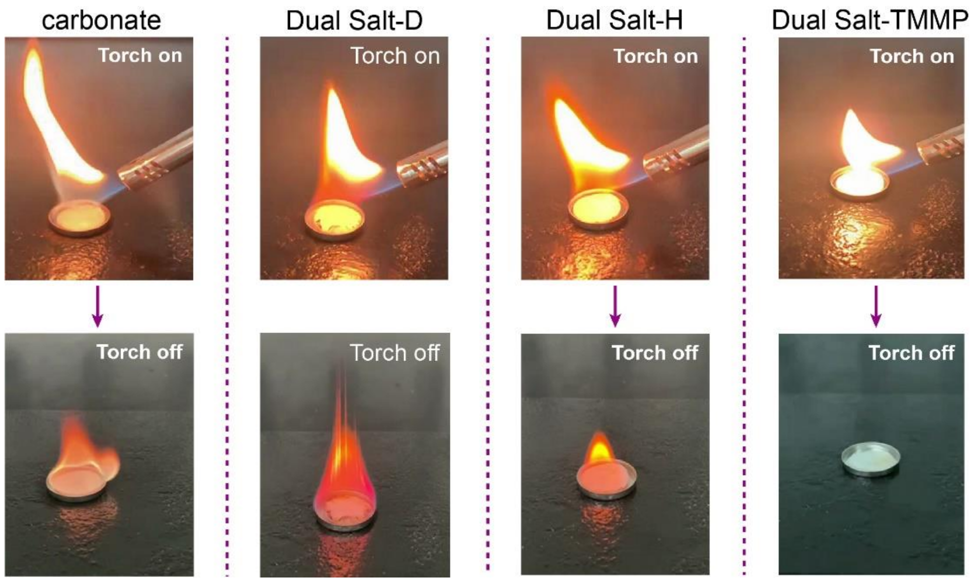

Figure S47. The flammability tests of different electrolytes: carbonate, Dual Salt-D, Dual Salt-H, and Dual Salt-TMMP.

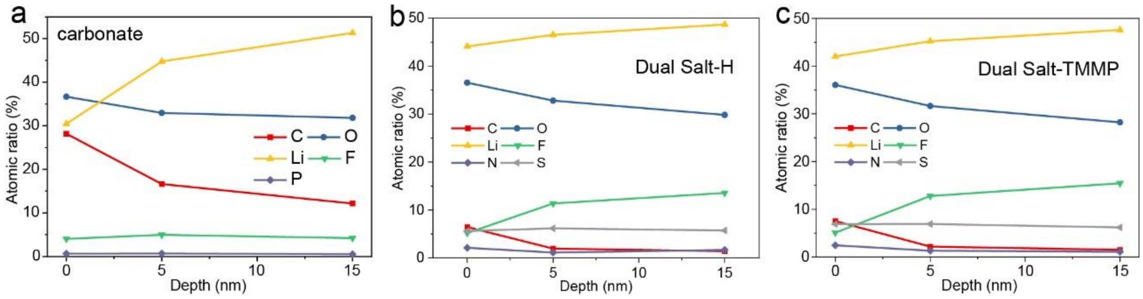

Figure S48. Atomic ratios during sputtering for Li anodes cycled in (a) carbonate, (b) Dual Salt-H, and (c) Dual Salt-TMMP electrolyte.

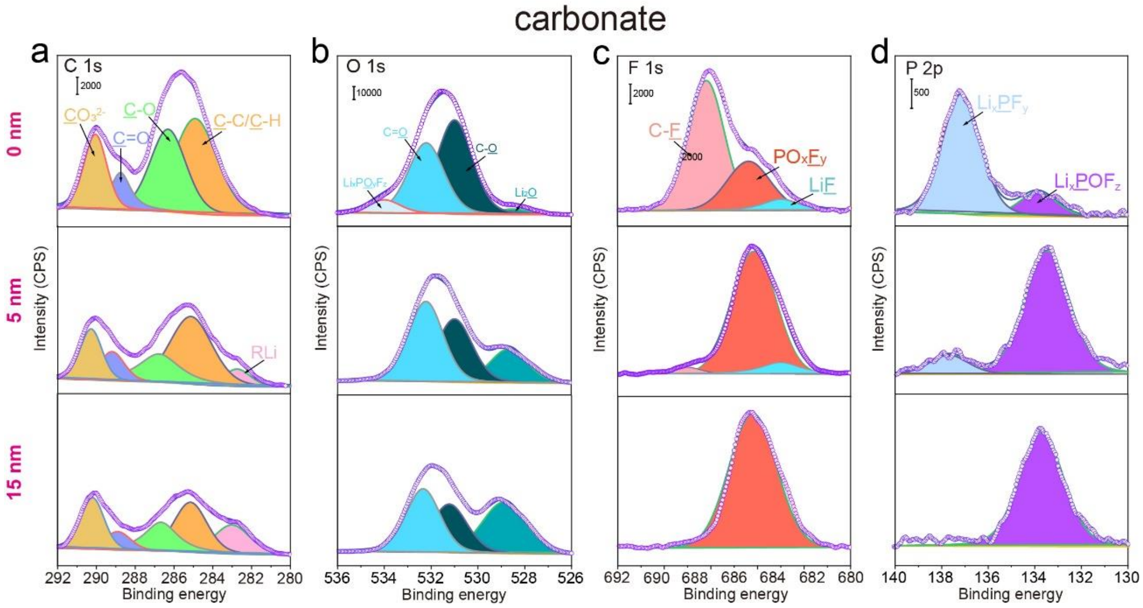

Figure S49. The (a) C 1s, (b) O 1s, (c) F 1s, and (d) P 2p XPS depth profiles of the Li metal cycled in carbonate electrolyte.

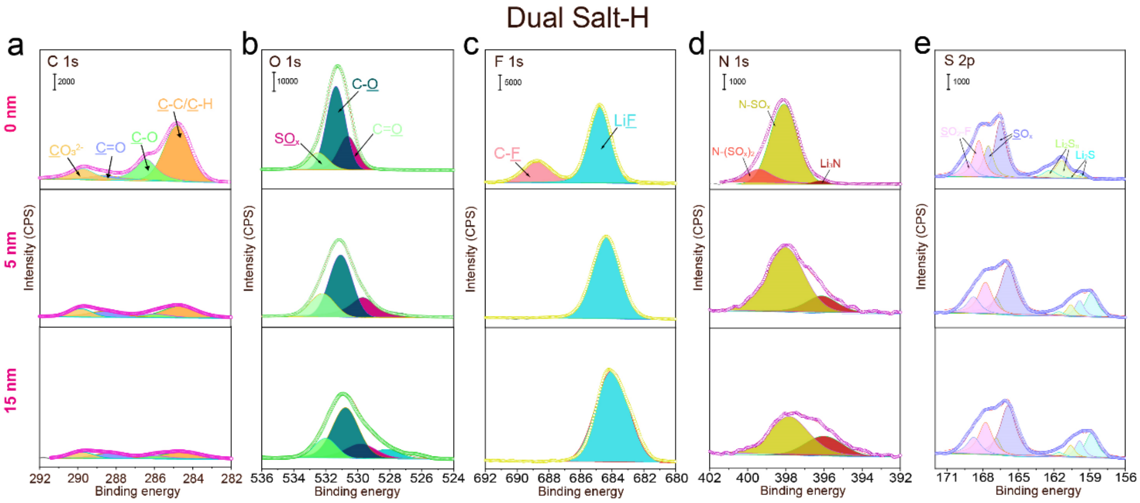

Figure S50. The (a) C 1s, (b) O 1s, (c) F 1s, (d) N 1s, and (e) S 2p XPS depth profiles of the Li metal cycled in Dual Salt-H electrolyte.

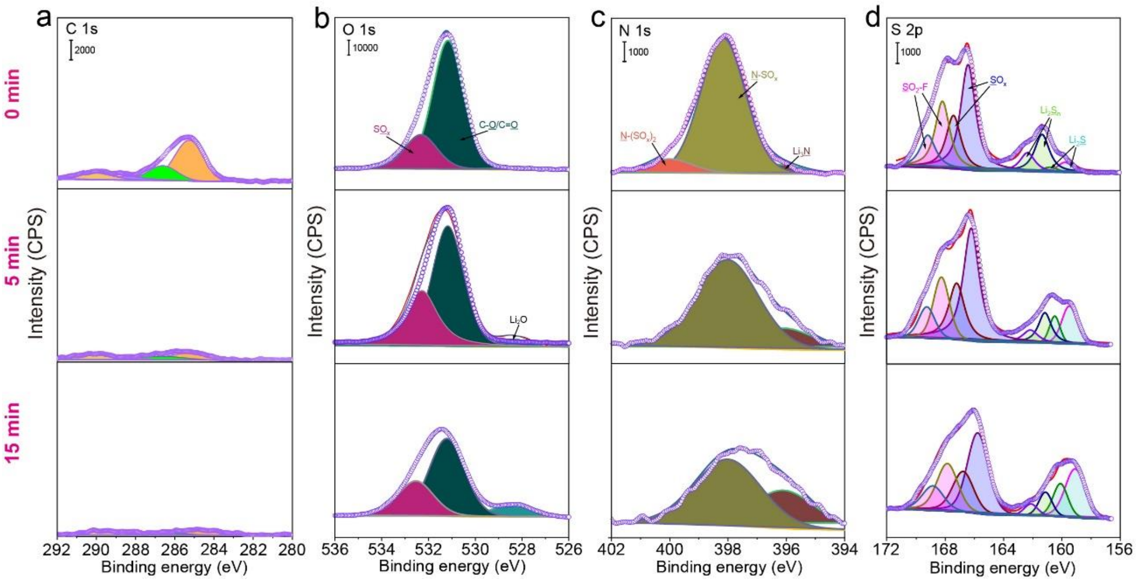

Figure S51. The (a) C 1s, (b) O 1s, (c) N 1s, and (d) S 2p XPS depth profiles of the Li metal cycled in Dual Salt-TMMP electrolyte.

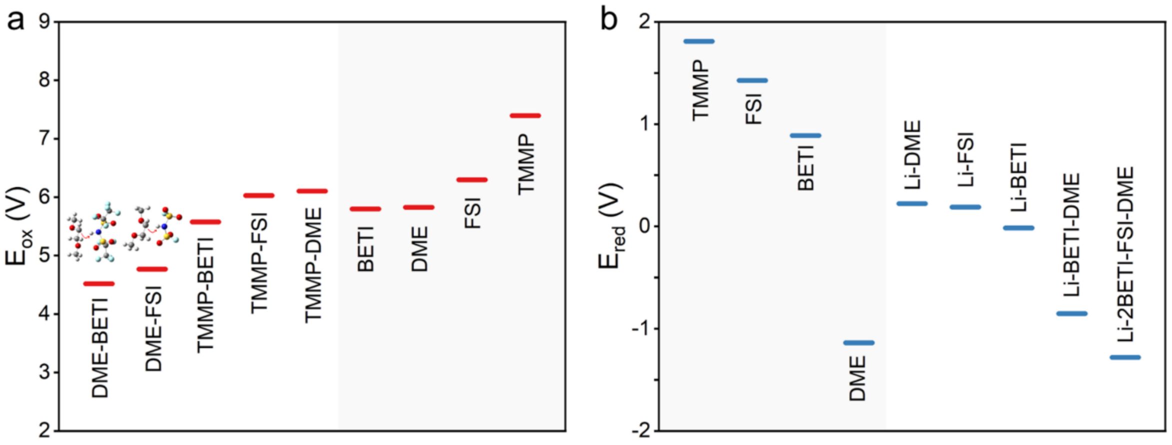

Figure S52. (a) Comparison of oxidation potentials of various molecules and complexes. (b) Comparison of reduction potentials of various molecules and complexes.

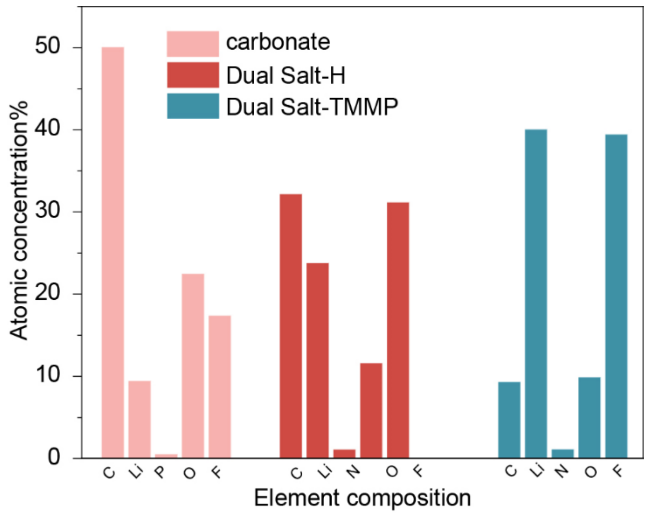

Figure S53. Atomic ratios during sputtering for NCM811 cycled in various electrolytes.

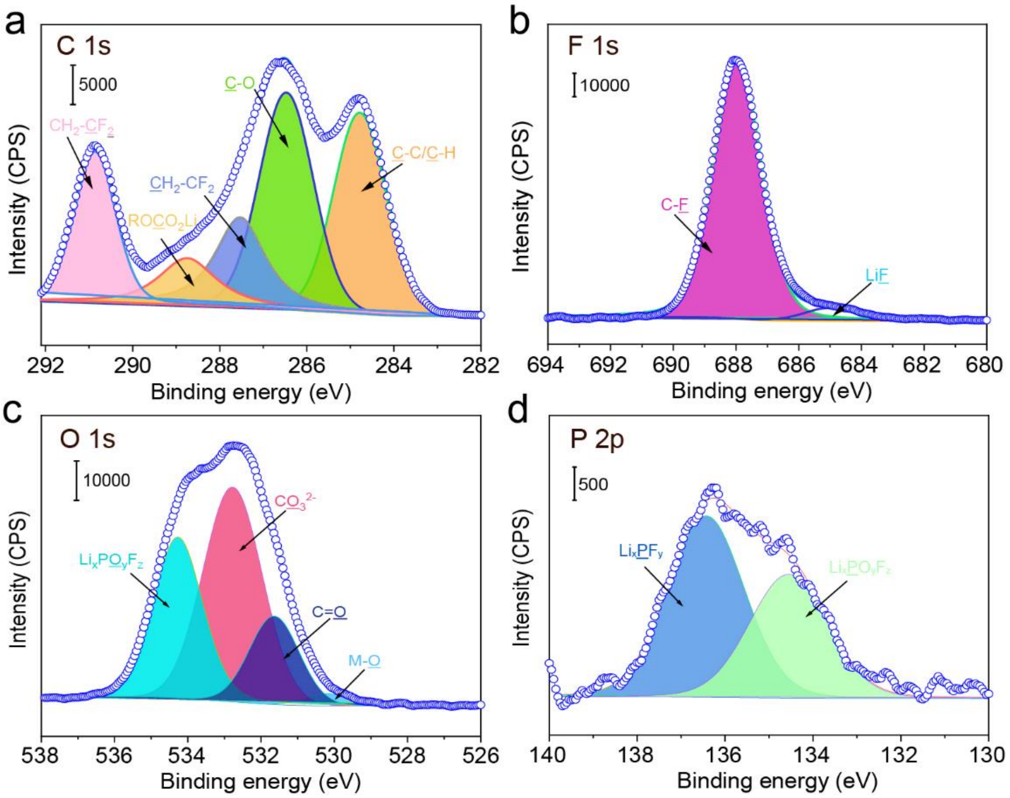

Figure S54. The XPS spectra of C 1s, F 1s, O 1s, and P 2p for NCM811 after 200 cycles in the carbonate electrolyte.

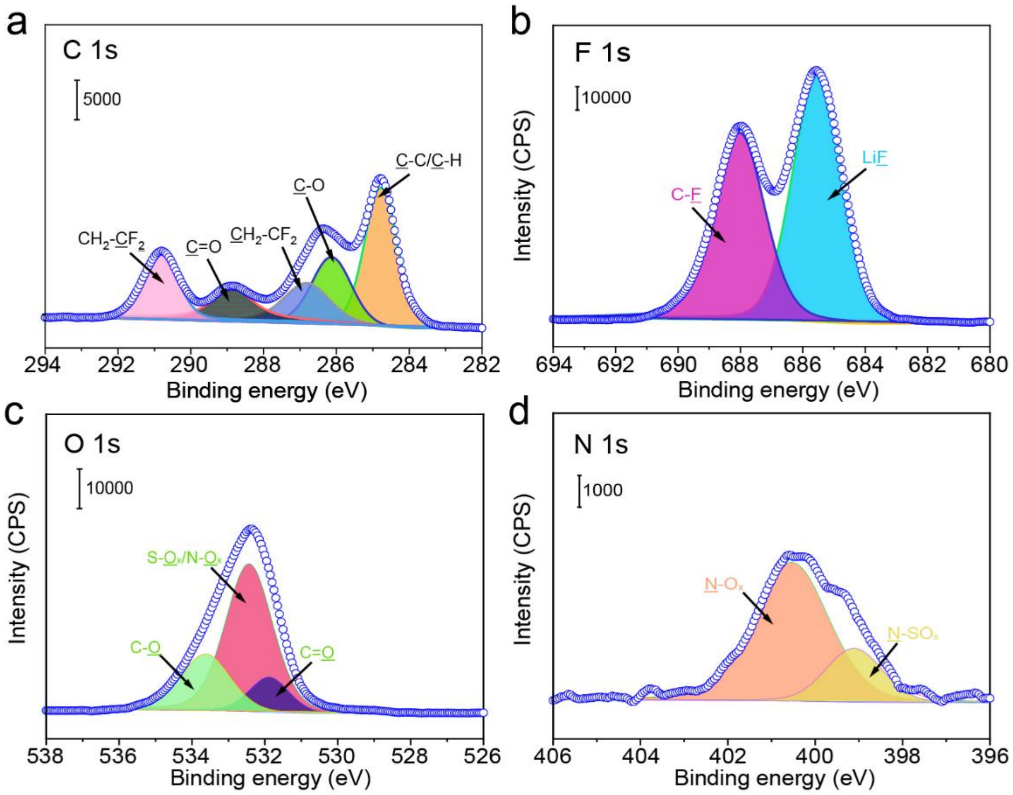

Figure S55. The XPS spectra of C 1s, F 1s, O 1s, and N 1s for NCM811 after 200 cycles in the Dual Salt-H electrolyte.

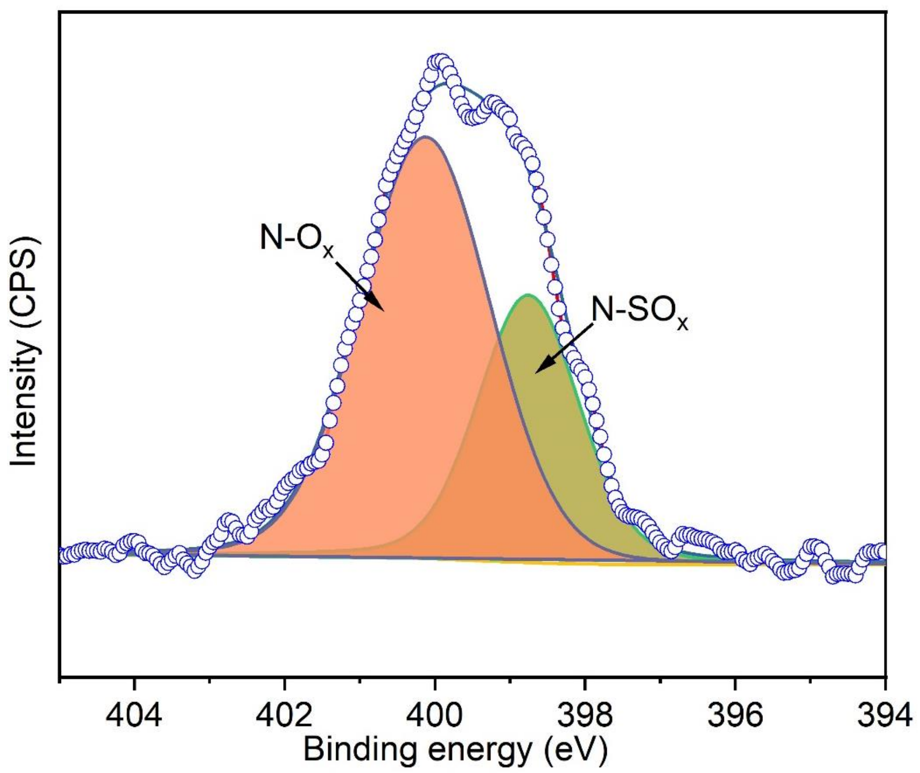

Figure S56. The N 1s XPS spectra of surface films formed on the NCM811 cathode from Li||NCM811 battery after cycling in Dual Salt-TMMP electrolyte.

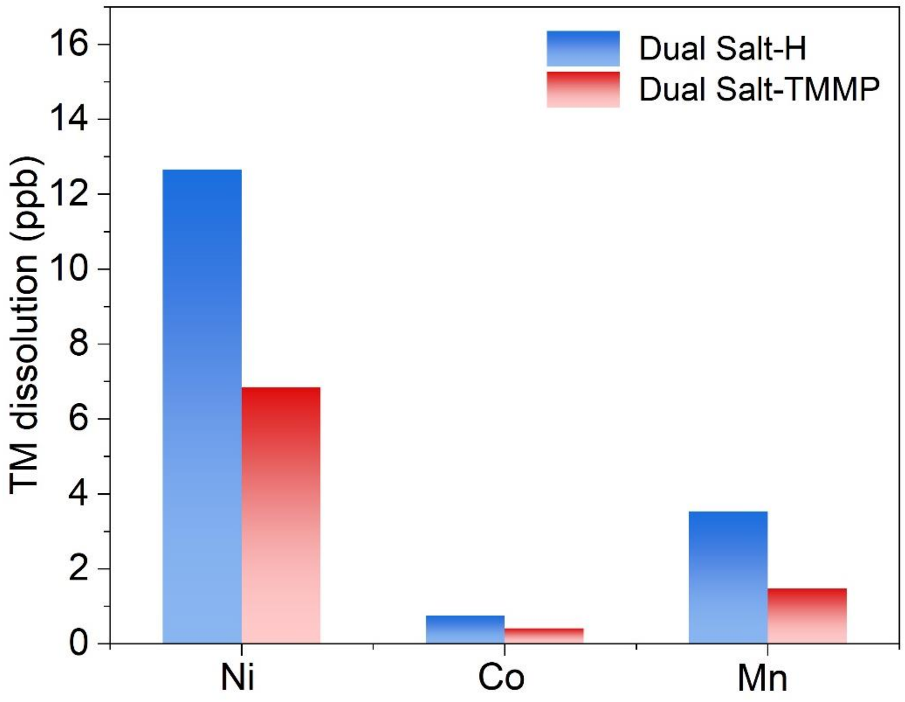

Figure S57. Transition metal ion dissolutions measured by ICP-MS after 50 cycles in Dual Salt-H and Dual Salt-TMMP electrolytes.

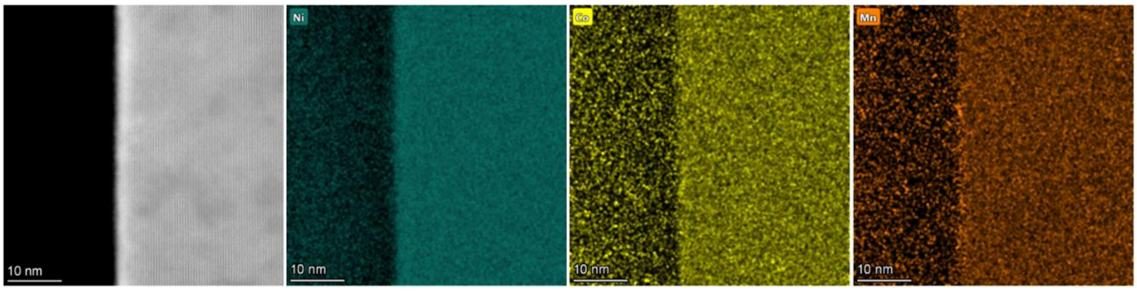

Figure S58. The elemental mapping images of Ni, Co and Mn of the cycled NCM811 cathodes in Dual Salt-TMMP electrolyte.

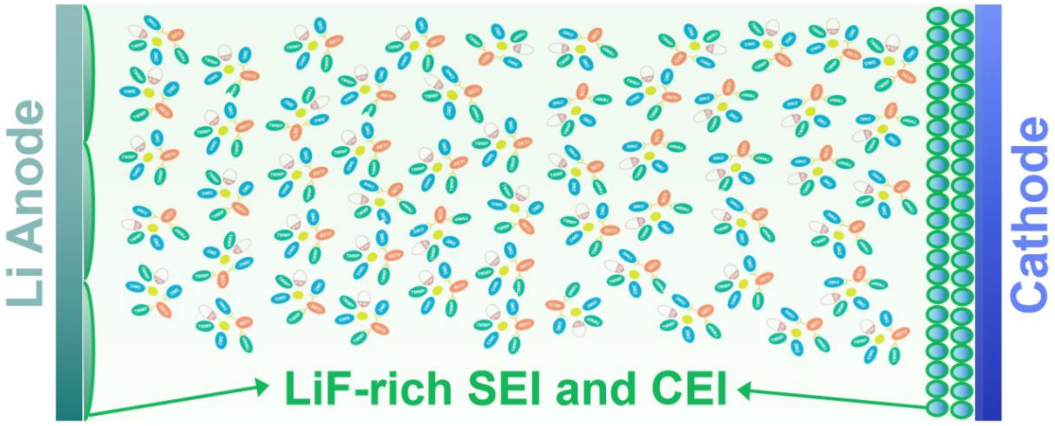

Figure S59. Illustration of SEI and CEI chemistries in Dual Salt- MMP electrolyte.

References

(1) Adams, B. D.; Zheng, J.; Ren, X.; Xu, W.; Zhang, J.-G. Accurate determination of coulombic efficiency for lithium metal anodes and lithium metal batteries. *Adv. Energy Mater.* **2018**, *8* (7), 1702097.

(2) Lee, C.; Yang, W.; Parr, R. G. Development of The colle-salvetti correlation-energy formula Into a Functional of the electron density. *Phys. Rev. B* **1988**, *37* (2), 785.

(3) Becke, A. D. A new mixing of hartree-fock and local density-functional theories. *J. Chem. phys.* **1993**, *98* (2), 1372-1377.

(4) Krishnan, R.; Binkley, J. S.; Seeger, R.; Pople, J. A. Self-consistent molecular orbital methods. XX. a basis set for correlated wave functions. *J. Chem. Phys.* **1980**, *72* (1), 650-654.

(5) McLean, A.; Chandler, G. Contracted gaussian basis sets for molecular calculations. i. second row atoms, Z= 11–18. *J. Chem. Phys.* **1980**, *72* (10), 5639-5648.

(6) Barnes, T. A.; Kaminski, J. W.; Borodin, O.; Miller III, T. F. Ab initio characterization of the electrochemical stability and solvation properties of condensed-phase ethylene carbonate and dimethyl carbonate mixtures. *J. Phys. Chem. C* **2015**, *119* (8), 3865-3880.

(7) Sun, H. COMPASS: an ab initio force-Field optimized for condensed-phase applications overview with details on alkane and benzene compounds. *J. Phys. Chem. B* **1998**, *102* (38), 7338-7364.
