# Supplementary material for: Molecularly Engineered Amphiphilic Anions Enable Flame-Retarding Fluorous Electrolytes for Lithium Metal Batteries
Source: ACS Cent Sci. 2025 Dec 25;12(1):63–74. doi: 10.1021/acscentsci.5c01711 (PMC12856666; doi:10.1021/acscentsci.5c01711)
Supplement: Supplementary file 8 [file oc5c01711_si_008.pdf]

oc-2025-01711t.R1

Name: Peer Review Information for "Molecularly Engineered Amphiphilic Anions Enable Flame-Retarding Fluorous Electrolytes for Lithium Metal Batteries"

First Round of Reviewer Comments

Reviewer: 1

Comments to the Author

1. The labeling of exotherm/endotherm in Fig. 1c appears inconsistent with the text. Please clarify the labeling and revise the text accordingly so readers are not misled.

2. In fig. 3a, the LiBETI–TMMP electrolyte shows the smallest voltage hysteresis. Could the authors compare this with the others and provide an explanation?

3. Beyond the Li||Cu cell's nucleation data, the Li||Li symmetric cell can better inform SEI formation/growth during re-plating. Please analyze representative cycles (e.g., 1/10/50) and comment on what this implies for SEI formation/growth.

4. Electrochemical impedance is underreported. Please include Nyquist plots for Li||Cu, Li||Li, and Li||cathode cells. Adding DRT analysis together with an accompanying equivalent circuit would substantially strengthen the electrochemical interpretation and the manuscript's overall rigor.

5. Depth-profiled XPS is shown only for Dual Salt–TMMP. Please add comparable SEI/CEI depth profiles for a control electrolyte, for example, LiBETI–TMMP and/or Dual Salt–H, to substantiate the claimed compositional advantages.

6. The context for the description of DME and TTE in 1H-NMR seems to need some correction. (Upon the introduction of TTE into DME, the 1H chemical shift from TTE … while chemical shifts of H(DME) decrease.)

7. For readability, please add the DSC plots with the exotherm/endotherm direction ("exo up/down") in the axis.

8. I think the flammability test and the thermal-abuse experiment are closely related. Consider grouping and presenting them in the same section/figure block (with updated figure numbering) so that safety metrics can be compared side-by-side under a consistent methodology.

Reviewer: 2

Comments to the Author

Chen et al. reported the rational design of safe and high-efficiency ether-based electrolytes for high-voltage lithium metal batteries. By introducing a flame-retardant diluent with an ultrahigh F/H atomic ratio of 4.33 and higher, and employing molecularly engineered anions bearing fluoro-alkyl moieties, they effectively addressed the immiscibility issue of highly fluorinated diluents through synergistic fluorophilic (F⋯F) and hydrogen-bonding (F⋯H) interactions. The resulting fluorous electrolytes enabled dendrite-free Li plating/stripping with a high Coulombic efficiency of ~99.5% and long-term cycling stability of Ni-rich NMC811 cathodes. Overall, this study is interesting and the results are promising. Its publication will be of great value to the battery research community. I recommend publication after minor revision, and my specific comments are as follows.

(1) Why is a clear position shown in Figure 1a for TMMP if its flash point cannot be quantified? Why is the flash point of TMMP unmeasurable?

(2) This study shows that the amphiphilic $BETI^-$ anion has strong interactions with the TMMP diluent. This finding seems to challenge the traditional view of diluents as purely inert, non-coordinating components in LHCEs. Does the introduction of this “non-inert” diluent, which interacts strongly with the anion, ultimately perturb the inner solvation structure of the Li^+ ion?

(3) The manuscript presents NMR data as key evidence for specific molecular interactions. However, the molecules involved in this article contain multiple hydrogen and fluorine atoms in distinct chemical environments. To better enhance the clarity and persuasiveness of the data, the authors should provide detailed signal assignments in the corresponding NMR spectra.

(4) Units and symbols are unclear or missing; for example, “ionic conductivity is 2.12 at 25 °C” lacks units (should be mS·cm-1 or S·m-1), and viscosity reported in “cp” should also include the SI unit (mPa·s).

(5) The discussion of the dual-salt strategy lacks consideration of cost, scalability, and environmental impact. Highly fluorinated compounds (e.g., BETI, TMMP, CFH) are costly and raise environmental and toxicity concerns. The authors should briefly address these aspects or note the associated limitations.

(6) There are grammatical errors and misspellings in the abstract and main text, such as "impatability", and the authors are advised to carefully proofread and thoroughly revise the grammar throughout the manuscript.

(7) Why were different CD rates chosen in Figure 3e?

(8) It is recommended to provide the quantitative analysis results of XPS. In addition, NMC811 is commonly written as NCM811.

Reviewer: 3

Comments to the Author

The manuscript designs "amphiphilic" anions to compatibilize highly fluorinated diluents with ether solvents and salts, aiming to deliver non-flammable, Li-metal-compatible electrolytes. The core formulation is a dual-salt ether electrolyte in which LiBETI is introduced alongside LiFSI to stabilize a DME plus TMMP diluent mixture that would otherwise phase-separate. The study combines ITC, multinuclear NMR including HOESY, ESI-MS speciation, short classical MD, small-cluster DFT, flammability tests, Li‖Cu CE by the Aurbach protocol, Li‖Li cycling, Li‖NMC811 coin and pouch cells, and a thermal abuse test with external heating. Performance trends are favorable versus the chosen internal controls and the fire behavior is clearly improved.

Major comments

1. Mechanistic evidence versus strength of methods

The central mechanism is that BETI− simultaneously engages in F⋯F interactions with the fluorous diluent and F⋯H interactions with the ether, bridging immiscible components. The experimental indicators are ITC sign changes, small chemical-shift movements, HOESY cross-peaks, and ESI-MS adducts, while calculations use 0 K gas-phase DFT of dimers and very short MD with a generic non-polarizable force field. These signals are suggestive, not decisive.

a) HOESY cross-peaks demonstrate proximity, not bond character or directionality. Please report mixing times, distance calibration, and control spectra that exclude nuclear Overhauser artifacts from spin diffusion.

b) ESI-MS is prone to gas-phase rearrangement of weak associates. A solution-phase quantification of association constants by VT-NMR, Raman band-shape analysis with constrained deconvolution, or diffusion-ordered NMR would be more convincing.

c) The MD windows are far too short to characterize mixing thermodynamics and outer-shell organization. Provide longer trajectories with a validated force field, density checks, and Kirkwood–Buff integrals or PMFs that directly show the proposed F-rich co-ordination to $BETI^-$.

d) The DFT dimer binding energies are small in magnitude and lack a solvation thermodynamic cycle. Present these as qualitative trends only and avoid quantitative causality.

2. Transport numbers and conductivity need statistics and cross-checks

Conductivity differences between the new and control electrolytes are modest, and the reported Li transference relies on self-diffusion ratios or Bruce–Vincent polarization which are method-dependent. Please report n for each metric, mean ± SD, fitting residuals for VTF and EIS, and add a complementary transference method such as restricted diffusion or electrophoretic NMR. Where $\sigma Li+$ is discussed, propagate uncertainties rather than quoting single values.

3. Electrochemical stringency and practicality

Li‖Cu CE values near 99.5 percent at 0.5 to 2 mA cm^{-2} are promising but depend sensitively on protocol details. State plating/stripping capacity per cycle, rest times, electrolyte volume per area, stack pressure, and the number of repeats. For Li‖Li and Li‖NMC811, provide areal loadings, N/P, and E/C ratios for every data set, include error bars across independent cells, and show post-mortem morphology to support claims of "dendrite-free" deposition. The pouch cell result is interesting; please include replicates and confidence intervals.

4. High-voltage stability and Al corrosion

Voltammetry and cathode cycling suggest tolerance up to about 4.4–4.5 V. To make a broad high-voltage claim, include constant-potential leakage-current holds on inert electrodes with area normalization and statistics, and quantify Al dissolution by ICP after potential holds. Cyclic scans alone are not sufficient for a general stability statement.

5. Flammability and thermal safety methodology

The closed-cup flash-point statement for TMMP and torch-flame tests show a clear safety advantage, but readers will expect standardized protocols and statistics. Please specify the standard used, sample mass and geometry, environment, and replicate counts for ignition and thermal-abuse tests. For the externally heated pouch cells, justify sensor placement and quantify onset and peak temperatures with uncertainties.

6. DFT HOMO/LUMO language

Where molecular orbital energies are listed and then qualitatively connected to reduction or oxidation tendencies, the analysis is not physically rigorous. Kohn–Sham HOMO/LUMO values are not redox potentials. For anions in particular, the "LUMO" has no direct meaning for the real condensed-phase electron uptake process unless an extra electron is explicitly included and stabilized by a proper solvation model. If the goal is to discuss reductive decomposability, compute solution-phase reduction free energies for explicit first-shell clusters or refrain from making such connections. This is important because widespread casual use of HOMO/LUMO for electrochemical stability has led to misleading interpretations in the electrolyte literature.

7. Internal consistency and unit hygiene

Viscosity, density, conductivity, and CE figures appear in multiple places with slightly different values or formats. Please consolidate into one summary table with units, significant figures, and n for each formulation. Ensure that all comparisons are at the same temperature.

Minor comments

1. Provide the equivalent circuits, fitting bounds, and residuals for every EIS plot used to extract Rbulk and interfacial resistances.

2. Report water content of each electrolyte mixture and the handling steps, since trace water strongly affects LiFSI chemistry.

3. Clarify whether any sample rinsing precedes XPS and how potential reconstruction artifacts were mitigated.

4. Quote separator type and thickness, stack pressure, and electrolyte volume for every electrochemical test figure.

Reviewer: 4

Comments to the Author

In current submission, Chen and co-workers focus on the miscibility issues of the fluorinated electrolytes, reporting the unique impact brought by a fluorinated anions containing bulky fluorinated groups. The experimental design is technically sound and the results are of critical importance for battery community. The conclusions are very convincing and the manuscript is well constructed. I would recommend the publication of this work after addressing minor issues as below.

(1) The anion BETI has been well studied by Passerini et al. (cf. Journal of The Electrochemical Society 149, A891-A897, doi:10.1149/1.1483098 (2002); Journal of The Electrochemical Society 149, A1282-A1285, doi:10.1149/1.1502688 (2002).), the authors are recommended to provide sufficient background for the use of BETI anion in this work.

(2) The NMR spectra of the LiBETI salt will be necessary to confirm its chemical structure and possible contamination of impurities.

(3) The authors may expand the discussion on future design of new anions and how this could be aligned with solvent optimization.

Author's Response to Peer Review Comments:

Dear Editor,

Thanks for give us the opportunity to revise our manuscript. We sincerely appreciate the great efforts from you and the reviewers for improving our manuscript. We have provided a detailed point-by-point response to the reviewer comments in the attachment. Please let us know if any more changes are needed.

Best regards,

Xiaodi Ren

Responses to reviewers' suggestions and comments for manuscript No. oc-2025-01711t

We would like to thank the reviewers for their valuable comments. We have incorporated most of the reviewers' comments and suggestions into the revised manuscript. We also provided detailed answers and explanations to the reviewers' other comments. The changes to the manuscript are marked yellow in this response and in the revised manuscript.

Reviewer #1:

1. The labeling of exotherm/endotherm in Fig. 1c appears inconsistent with the text. Please clarify the labeling and revise the text accordingly so readers are not misled.

Response: We thank the reviewer for pointing this out. We have carefully checked Fig. 1c and realized that the labeling of exotherm and endotherm was indeed incorrect. The figure has now been corrected, and the text has been revised accordingly to accurately reflect the thermal events.

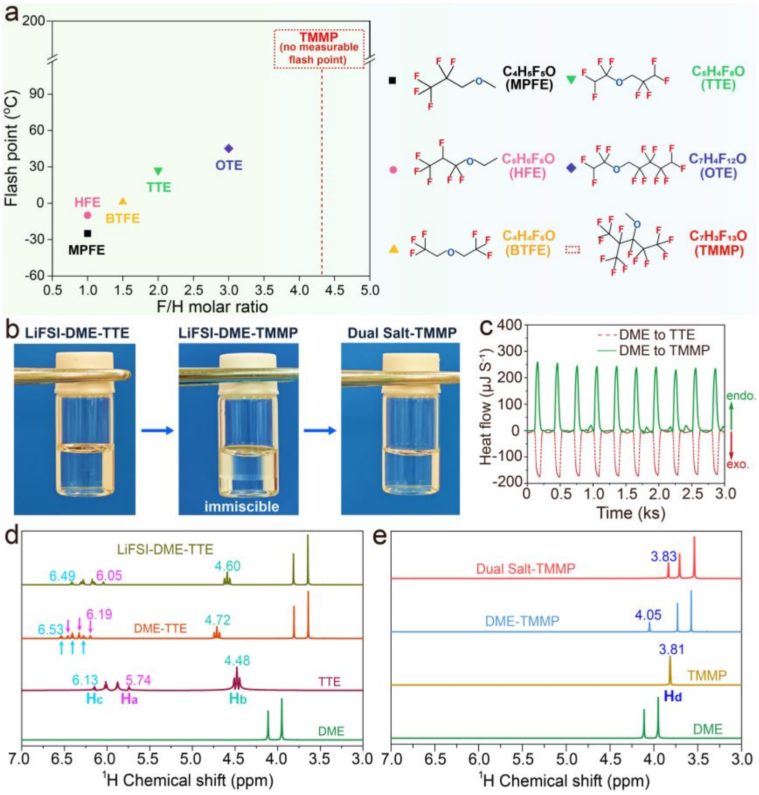

Figure 1. (a) Flashpoints for fluorinated ethers with different F/H molar ratios and their molecular structures. (b) The photos of LiFSI-DME-TTE, LiFSI-DME-TMMP (1:2:2 by molar ratio), and Dual Salt-DME-TMMP electrolytes. (c) Nano ITC data for mixing DME with TTE or TMMP. (d)-(e) ^{1}H NMR spectrum of different diluents, solvent mixture, and electrolytes.

2. In Fig. 3a, the LiBETI-TMMP electrolyte shows the smallest voltage hysteresis. Could the authors compare this with the others and provide an explanation?

Response: We thank the reviewer for this helpful comment. The smallest voltage hysteresis of the LiBETI-TMMP electrolyte mainly arises from the following two factors: **superior wettability and the formation of a less protective interphase**.

Wettability: As shown in Figure S18, the carbonate and Dual Salt-H electrolytes exhibit poor wettability on both the separator and Li metal (**Figure S18**). This inherent limitation of carbonate-based and high-concentration electrolyte systems directly leads to their large initial voltage hysteresis (**Figure 3a**). In contrast, the introduction of the TMMP diluent lowers viscosity and improves electrolyte spreading, enabling the LiBETI-TMMP and Dual Salt-TMMP electrolyte to achieve a significantly reduced overpotential, both initially and over the long term (**Figure S18**).**Electrode-electrolyte interface:** Because the FSI^- is more readily reduced than $BETI^-$, the Dual Salt-TMMP electrolyte rapidly forms an inorganic-rich SEI (**Figure S49-51**). Although this protective interphase introduces a higher Li^+ transport barrier and thus slightly increases the voltage hysteresis, this trade-off is justified as the resulting robust SEI markedly improves long-term anode compatibility compared to the LiBETI-TMMP system (Figure 3a and S19).

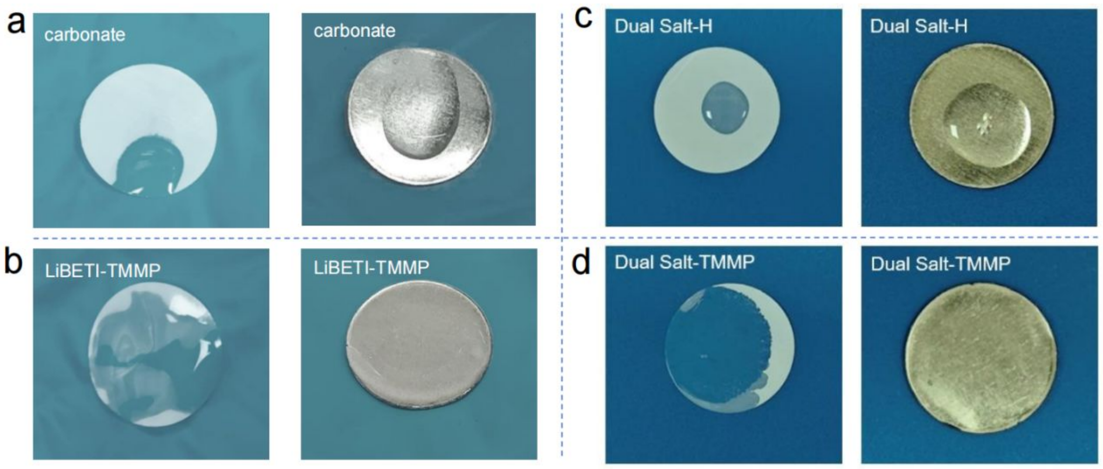

Figure S18. Comparison of electrolyte wettability on the separator and Li anode: (a) for carbonate, (b) for LiBETI-TMMP, and (c) for Dual Salt-H, and (d) for Dual Salt-TMMP.

Changes in the manuscript:

A paragraph has been added on Page 6 to elaborate on this point:

"In contrast, the LiBETI-TMMP electrolyte exhibits the smallest voltage hysteresis among all systems, likely due to its enhanced wettability and Li^+ transport kinetics (Figure S15). However, the higher reduction stability of $BETI^-$ limits LiF formation, resulting in a less protective SEI and a slightly lower CE."

3. Beyond the Li||Cu cell's nucleation data, the Li||Li symmetric cell can better inform SEI formation/growth during re-plating. Please analyze representative cycles (e.g., 1/10/50) and comment on what this implies for SEI formation/growth.

Response: We thank the reviewer for this insightful comment. To further investigate SEI formation and growth behavior, Li||Li symmetric cells were tested using various electrolytes, as shown in **Figure S24**.

SEI formation: The Li||Li symmetric data are consistent with the Li||Cu nucleation overpotential results. Dual Salt-TMMP shows the lowest nucleation overpotential, indicating the formation of a kinetically favorable SEI. Moreover, the absence of a noticeable rise in the deposition/stripping-end overpotential suggests that the SEI in Dual Salt-TMMP remains uniform and protective throughout cycling.

SEI growth: All three electrolytes display an initial decrease in overpotential followed by a subsequent increase, reflecting the typical SEI evolution process, including formation, rearrangement, and repair. However, the magnitude of these changes differs significantly among the systems. Dual Salt-TMMP shows only minimal variation, indicating a uniform, robust, and mechanically stable SEI that maintains homogeneous Li^+ flux and promotes dense lithium deposition. In contrast, both carbonate and Dual Salt-H exhibit large increases and late-cycle instability, consistent with repeated fracture and accumulated byproducts, which continually raise interfacial impedance.

These results clearly show that Dual Salt-TMMP forms the most stable and slowest-growing SEI, while the carbonate and Dual Salt-H electrolytes suffer from progressive SEI thickening and instability during long-term cycling.

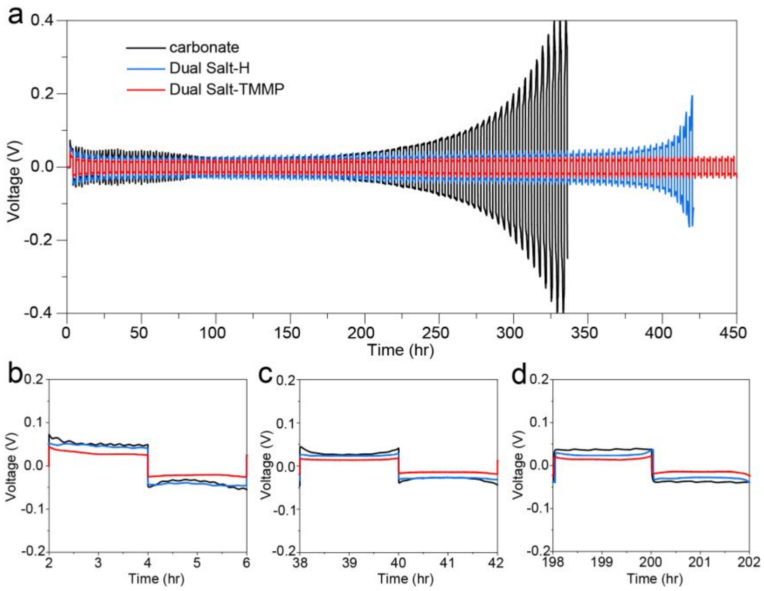

Figure S24. Voltage profiles of Li||Li symmetric cells using various electrolytes: (a) full cycling process; (b-d) magnified views of the 1st, 10th, and 50th cycles, respectively.

Changes in the manuscript:

A paragraph has been added on Pages 6 to elaborate on this point:

"As illustrated in Figure S24a, the Dual Salt-TMMP electrolyte exhibits the most stable cycling with minimal polarization over 450 hours, while the carbonate and Dual Salt-H electrolytes display gradually increased voltage hysteresis. The magnified views of the 1st, 10th, and 50th cycles (Figures S24b-S24d) show that the Dual Salt-TMMP cell maintains the lowest and most stable overpotential during repeated Li plating/stripping, indicating the formation of a robust and uniform SEI that effectively mitigates dendritic growth and interfacial impedance accumulation. In contrast, the carbonate-based electrolyte suffers from continuous voltage increase, suggesting unstable SEI formation and parasitic reactions, whereas the Dual Salt-H system exhibits intermediate stability. These findings corroborate the Li||Cu results and demonstrate that the TMMP-containing dual-salt system facilitates the development of a stable, ionically conductive SEI, thereby ensuring superior interfacial reversibility."

4. Electrochemical impedance is underreported. Please include Nyquist plots for Li||Cu, Li||Li, and Li||cathode cells. Adding DRT analysis together with an accompanying equivalent circuit would substantially strengthen the electrochemical interpretation and the manuscript's overall rigor.

Response: We sincerely appreciate the reviewer's valuable suggestion regarding the inclusion of more comprehensive electrochemical impedance analysis. In response, we performed additional EIS measurements for Li||Cu, Li||Li, and Li||NCM811 cells using the three representative electrolytes: carbonate, Dual Salt-H, and Dual Salt-TMMP. The corresponding Nyquist plots and DRT analyses are presented in **Figures S22, S25, and S37**, and the equivalent circuit used for fitting is shown in **Figure S24**.

For the Li||Cu cells (**Figures S22-S23**), the impedance of the carbonate electrolyte increases rapidly upon cycling, indicating unstable SEI growth and continuous interfacial reactions. For Dual Salt-H, the reduced impedance may arise from the increased electrochemically active surface area associated with rough Li deposition, consistent with its moderate Coulombic efficiency. In comparison, Dual Salt-TMMP more effectively regulates Li morphology and forms a compact, stable SEI, leading to the smallest variation in impedance and the most stable DRT features after 20 cycles. These results demonstrate that Dual Salt-TMMP promotes the formation of a dense and ionically conductive SEI, which stabilizes the Li surface and accounts for the significantly improved Li plating/stripping reversibility observed in Li||Cu cells.

Similar trends are observed in Li||Li symmetric cells (**Figure S25**) and Li||NCM811 full cells (**Figure S37**). While the carbonate electrolyte maintains high interfacial impedance throughout cycling, both Dual Salt-H and Dual Salt-TMMP exhibit a pronounced impedance decrease. Among them, Dual Salt-TMMP consistently achieves the lowest overall impedance and most stable DRT response, demonstrating superior Li^+ transport kinetics and interfacial stability at both electrodes.

Collectively, these EIS results confirm that the Dual Salt-TMMP electrolyte facilitates the most stable SEI and CEI across all cell configurations, enabling highly reversible Li^+ transport and sustained interfacial stability.

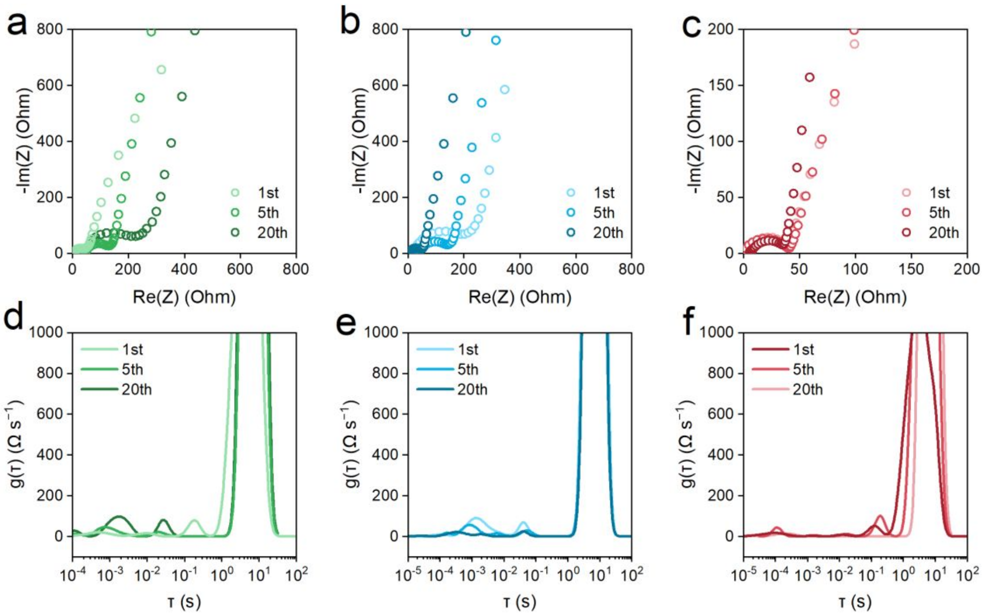

Figure S22. EIS and corresponding DRT analysis of Li||Cu cells using different electrolytes: (a, d) carbonate, (b, e) Dual Salt-H, and (c, f) Dual Salt-TMMP.

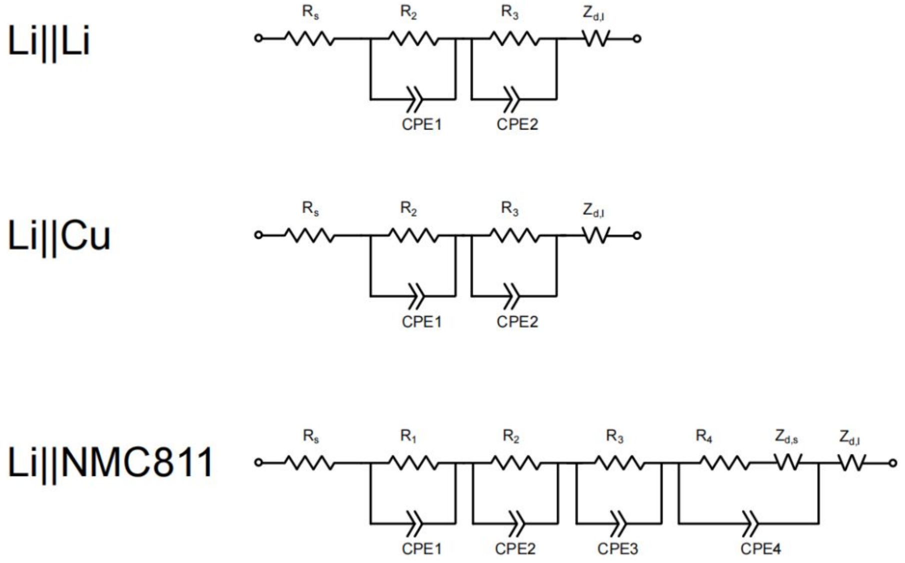

Figure S23. The equivalent circuit employed for fitting the electrochemical impedance spectra (EIS) of Li||Cu, Li||Li, and Li||NCM811 cells.

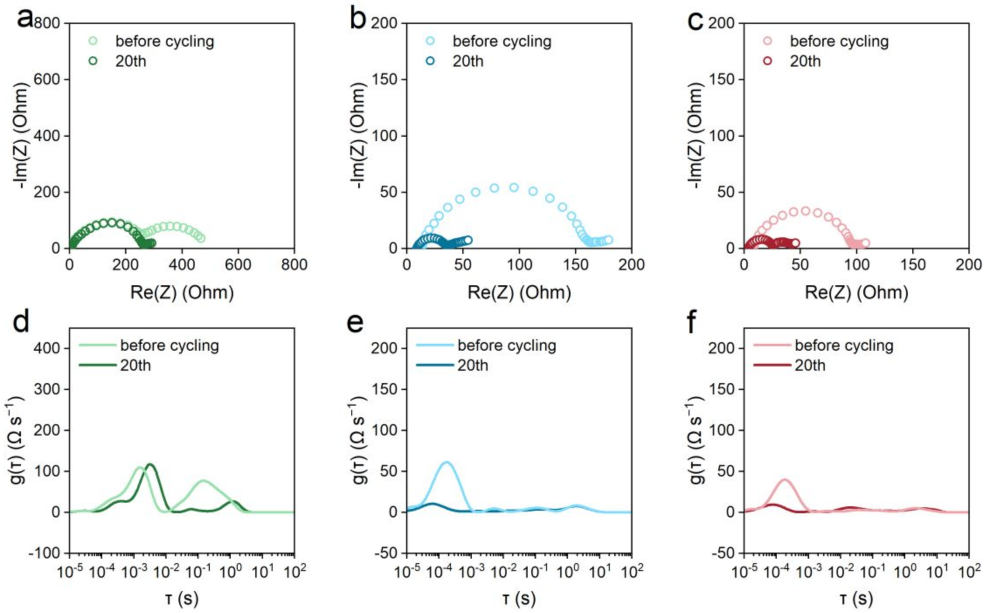

Figure S25. EIS and corresponding DRT analysis of Li||Li cells using different electrolytes: (a, d) carbonate, (b, e) Dual Salt-H, and (c, f) Dual Salt-TMMP.

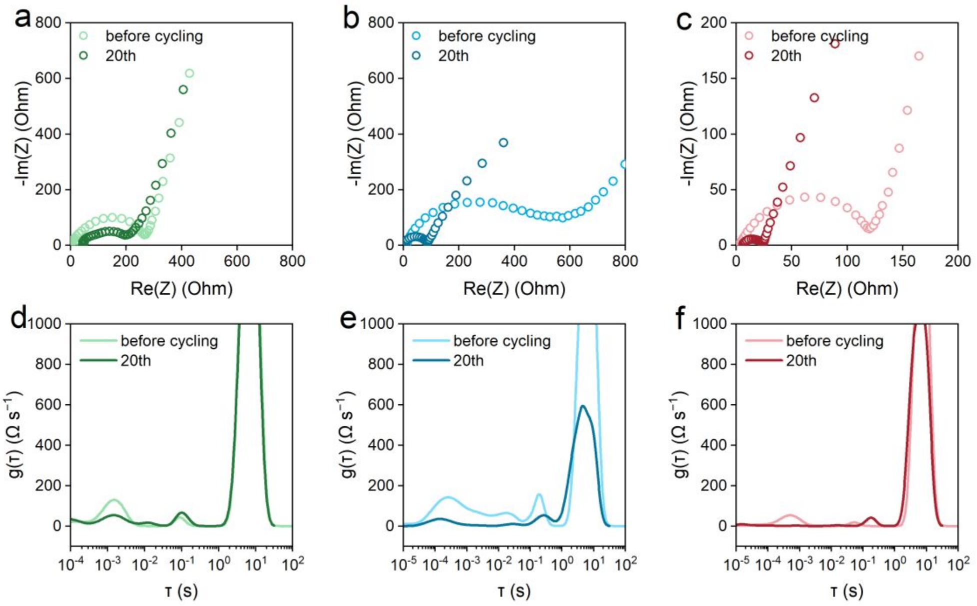

Figure S37. EIS and corresponding DRT analysis of NCM811||Li cells using different electrolytes: (a, d) carbonate, (b, e) Dual Salt-H, and (c, f) Dual Salt-TMMP.

Changes in the manuscript:

A paragraph has been added on **Pages 6** to elaborate on this point:

➢ "Consistently, electrochemical impedance spectroscopy (EIS) measurements (Figures S22-S23) reveal that, compared with the carbonate and Dual Salt-H

electrolytes, the Dual Salt-TMMP system exhibits minimal impedance growth and highly stable distribution of relaxation time (DRT) features, further evidencing enhanced interfacial stability and Li plating/stripping behavior."

- "EIS measurements further confirm this trend (Figure S23 and S25), with the Dual Salt-TMMP electrolyte exhibiting the lowest and most stable interfacial resistance and consistent DRT features over cycling, highlighting its superior ability to maintain a highly conductive and stable SEI compared to the other electrolytes."

A paragraph has been added on **Pages 7** to elaborate on this point:

- "As shown in Figures S23 and S37, the EIS results indicate that the Dual Salt-TMMP electrolyte effectively suppresses the growth of interfacial resistance over 25 cycles, whereas the carbonate system exhibits a pronounced increase in polarization. Corresponding DRT analysis further confirms that Dual Salt-TMMP minimizes both SEI and charge-transfer related relaxation processes, highlighting improved Li^+ transport and enhanced interfacial stability at both electrodes."

5. Depth-profiled XPS is shown only for Dual Salt-TMMP. Please add comparable SEI/CEI depth profiles for a control electrolyte, for example, LiBETI-TMMP and/or Dual Salt-H, to substantiate the claimed compositional advantages.

Response: We thank the reviewer for this constructive suggestion. In response, we have added the depth-profiled XPS results for the SEI formed in the carbonate and Dual Salt-H electrolytes (see new **Figures S48-S51**). Since the main focus of this work is to evaluate the interfacial stability of the anode, these additional depth profiles provide a direct comparison of SEI composition and evolution under different electrolyte systems.

Furthermore, we have also included the surface XPS analyses of the CEI for both carbonate and Dual Salt-H electrolytes to give a more comprehensive understanding of interfacial chemistry (see revised **Figures S53-S55**). These results clearly highlight the distinct compositional differences that account for the enhanced electrochemical stability of the Dual Salt-TMMP system.

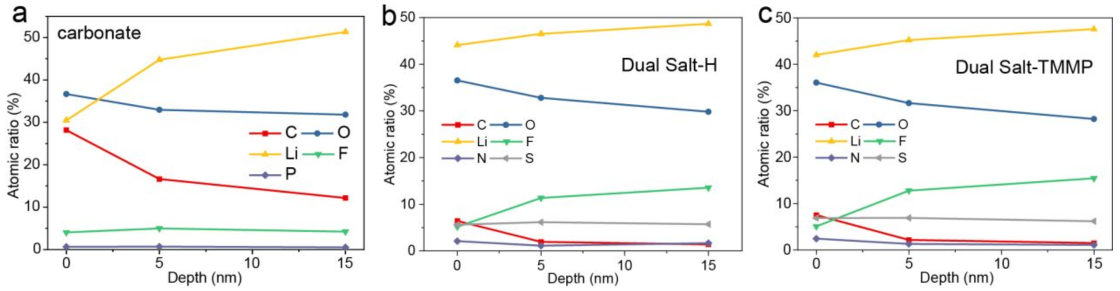

Figure S48. Atomic ratios during sputtering for Li anodes cycled in (a) carbonate, (b) Dual Salt-H, and (c) Dual Salt-TMMP electrolytes.

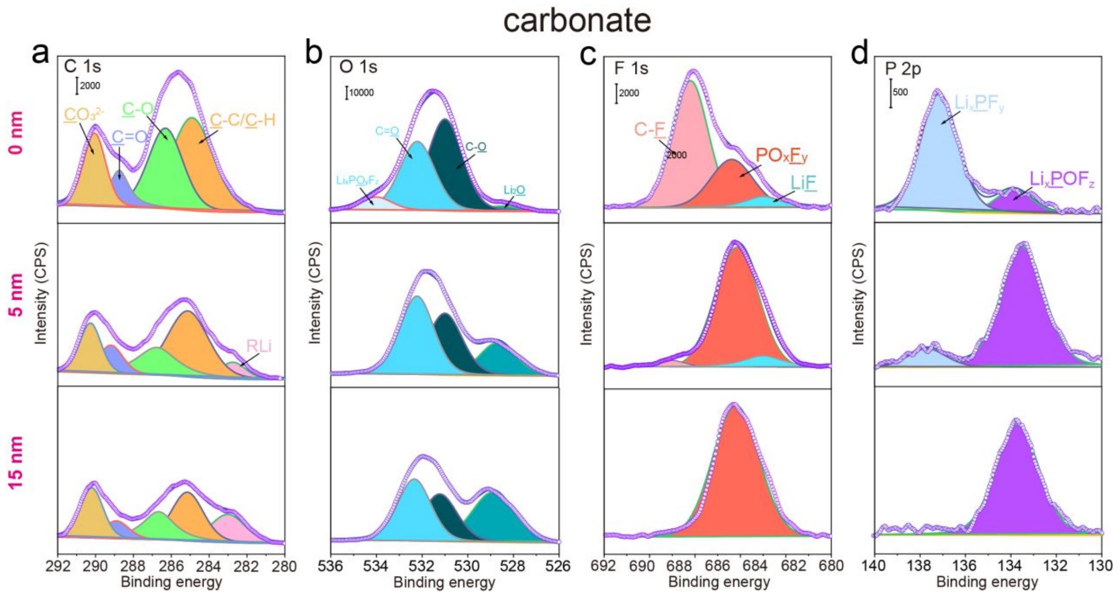

Figure S49. The (a) C 1s, (b) O 1s, (c) F 1s, and (d) P 2p XPS depth profiles of the Li metal cycled in carbonate electrolyte.

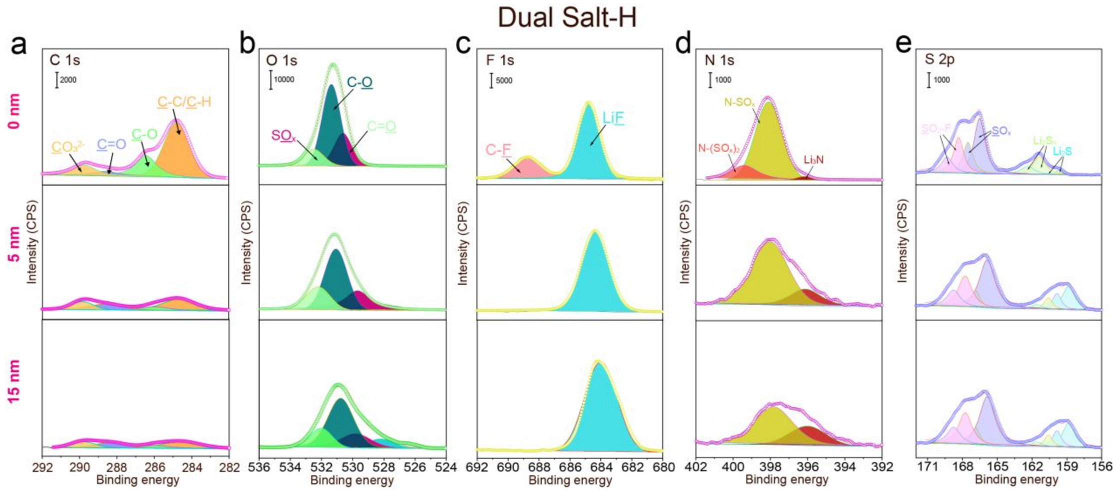

Figure S50. The (a) C 1s, (b) O 1s, (c) F 1s, (d) N 1s, and (e) S 2p XPS depth profiles of the Li metal cycled in Dual Salt-H electrolyte.

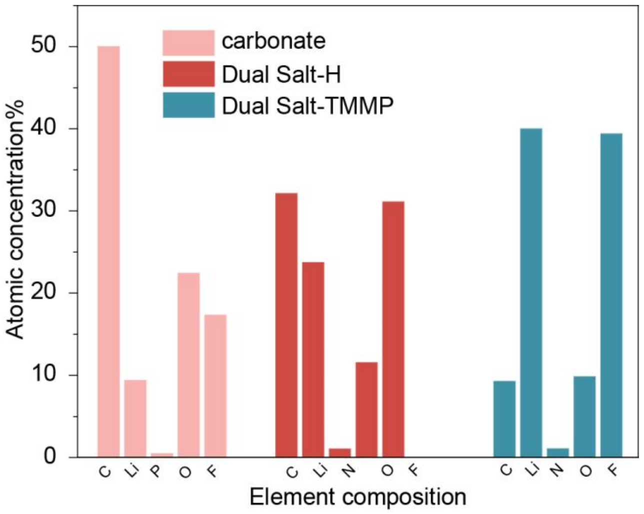

Figure S53. Atomic ratios during sputtering for NCM811 cycled in various electrolytes.

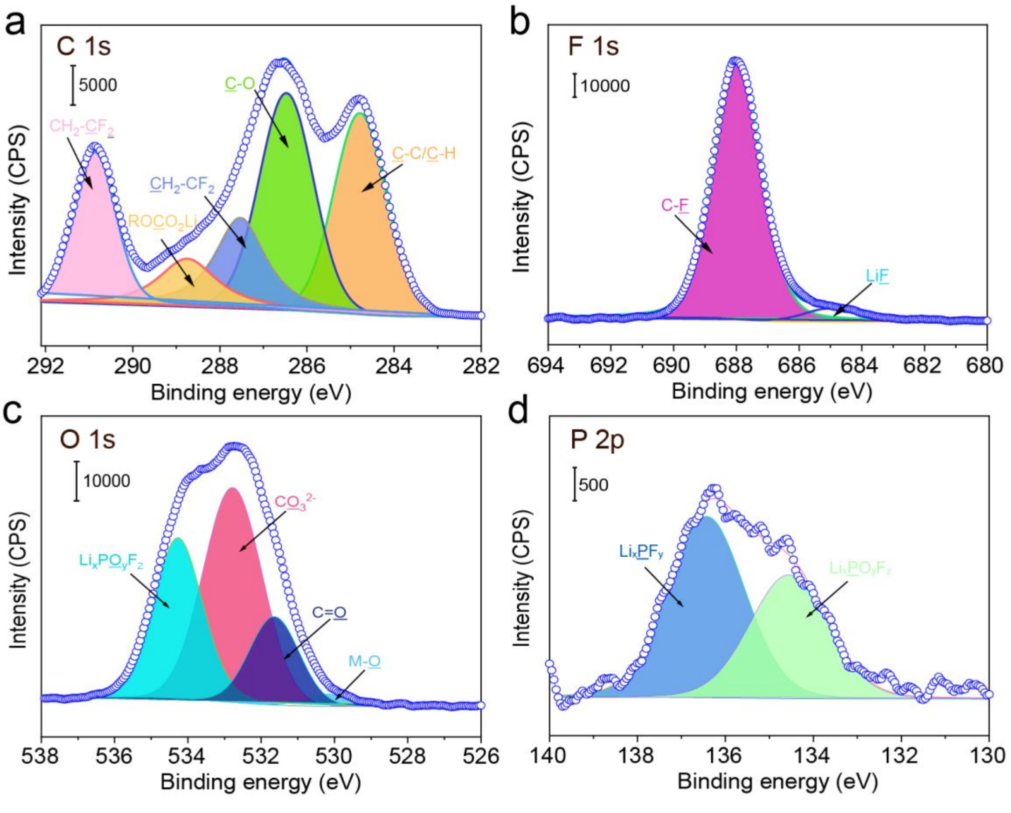

Figure S54. The XPS spectra of C 1s, F 1s, O 1s, and P 2p for NCM811 after 200 cycles in the carbonate electrolyte.

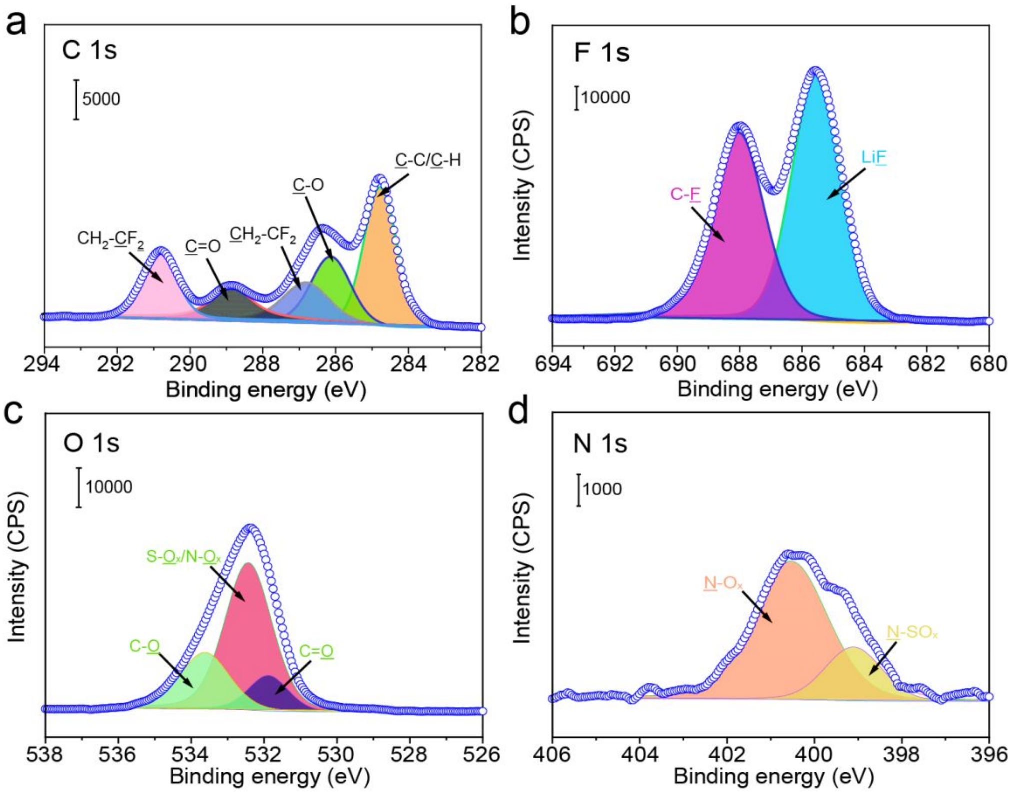

Figure S55. The XPS spectra of C 1s, F 1s, O 1s, and N 1s for NCM811 after 200 cycles in the Dual Salt-H electrolyte.

Changes in the manuscript:

A paragraph has been added on **Page 9** to elaborate on this point:

"XPS depth profiling analysis was conducted to investigate the detailed composition and structure of the SEI layer formed on Li anodes in the Dual Salt-TMMP, carbonate, and Dual Salt-H electrolytes, providing insights into their interfacial compatibility with Li metal (Figures 4a-4c and Figures S48-S51). As shown in Figures 4a-4c and S50-S51, the SEI derived from the Dual Salt-TMMP electrolyte contains a markedly higher fraction of inorganic species, particularly LiF, compared with those formed in the carbonate and Dual Salt-H systems, indicating a denser and more stable interphase. In contrast, the SEI in the carbonate electrolyte (Figure S49) is dominated by organic components such as $ROCO_2Li$ and C-O species, with only limited LiF and Li_2O, suggesting a loose and chemically unstable structure. The enrichment of LiF in the Dual Salt-TMMP SEI is beneficial, as LiF possesses excellent electronic insulating properties and mechanical robustness, effectively mitigating side reactions between Li

metal and the electrolyte.[49] This enrichment implies efficient decomposition of fluorinated anions on the Li surface, consistent with previous observations in localized high-concentration electrolytes.[50, 51] In addition, the XPS spectra of S 2p and N 1s (Figures S50-S51) confirm the presence of inorganic components such as SO_x, N-SO_x, Li_3N, and Li_2S in both the Dual Salt-H and Dual Salt-TMMP systems, further contributing to the stability of the SEI. Overall, these results confirm that the Dual Salt-TMMP electrolyte enables the formation of a robust, inorganic-rich SEI that ensures superior interfacial stability against Li metal."

A paragraph has been added on Page 9 to elaborate on this point:

"In addition, TMMP can also participate in LiF generation through oxidative decomposition at high voltages, as its TMMP-anion complex exhibits a lower-lying HOMO than the isolated TMMP molecule (Figure S52). In contrast, the CEI formed in the carbonate and Dual Salt-H electrolytes (Figures S54-S55) contains a higher fraction of organic species such as $ROCO_2Li$ and C-O components, along with weaker LiF and N-SO_x signals, indicating a less stable interphase. Notably, the CEI in the Dual Salt-TMMP electrolyte exhibits a markedly higher atomic ratio of lithium (Figure S53), indicating efficient electrolyte decomposition and the consequent formation of a protective layer. The absence of pronounced metal-oxygen (M-O) signals on the cycled NCM811 surface (Figure 4f) suggests effective suppression of transition metal dissolution. These findings demonstrate that the Dual Salt-TMMP electrolyte facilitates the formation of a Li-rich and robust CEI, which effectively stabilizes the cathode–electrolyte interface under high-voltage conditions."

6. The context for the description of DME and TTE in ^{1}H-NMR seems to need some correction. (Upon the introduction of TTE into DME, the ^{1}H chemical shift from TTE … while chemical shifts of H(DME) decrease.)

Response: We thank the reviewer for pointing this out. We agree that the original wording could be misleading regarding the ^{1}H-NMR chemical shift changes upon

mixing TTE with DME. We have revised the sentence to clarify the reference and the direction of the shifts. The corrected version now reads:

"Upon mixing TTE with DME, the ^{1}H signals of TTE shift downfield ($\Delta\delta_{Ha}$ = 0.45 ppm, $\Delta\delta_{Hb}$ = 0.24 ppm, and $\Delta\delta_{Hc}$ = 0.40 ppm) (Figure 1d and Figures S3-S4) relative to those in pure TTE, whereas the ^{1}H signals of DME shift upfield."

7. For readability, please add the DSC plots with the exotherm/endotherm direction ("exo up/down") in the axis.

Response: We thank the reviewer for this helpful suggestion. The DSC plots have been updated to indicate the exotherm/endotherm direction on the axis ("Exo up"), improving clarity and readability. The revised Figures 3h and S32 are shown below.

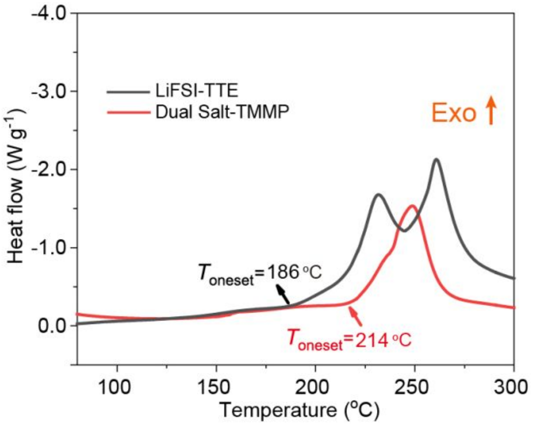

Figure 3h. DSC thermograms for delithiated NCM811 with different electrolytes.

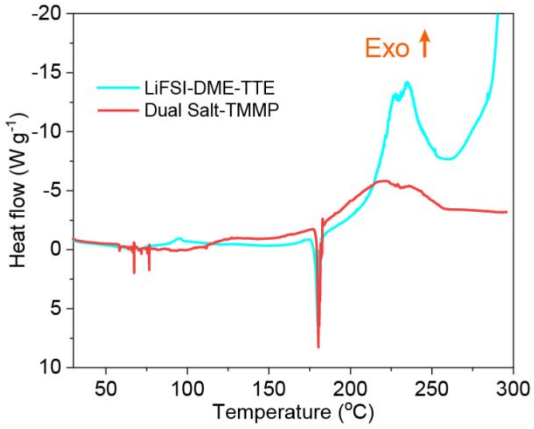

Figure S32. DSC traces for LiFSI-TTE, and Dual Salt-TMMP electrolytes with deposited Li metal.

8. I think the flammability test and the thermal-abuse experiment are closely related. Consider grouping and presenting them in the same section/figure block (with updated figure numbering) so that safety metrics can be compared side-by-side under a consistent methodology.

Response: We sincerely thank the reviewer for this valuable suggestion. We agree that presenting the flammability test and thermal-abuse experiment together can enhance clarity and comparability. Accordingly, the flammability test previously shown in Figure 2h has been moved to the Supporting Information, and its description has been integrated with the thermal-abuse experiment section in the manuscript. Figure 2 and Figure S15 have been revised accordingly to reflect these changes. This reorganization allows all safety-related results to be discussed under a consistent methodology and improves the overall coherence of data presentation.

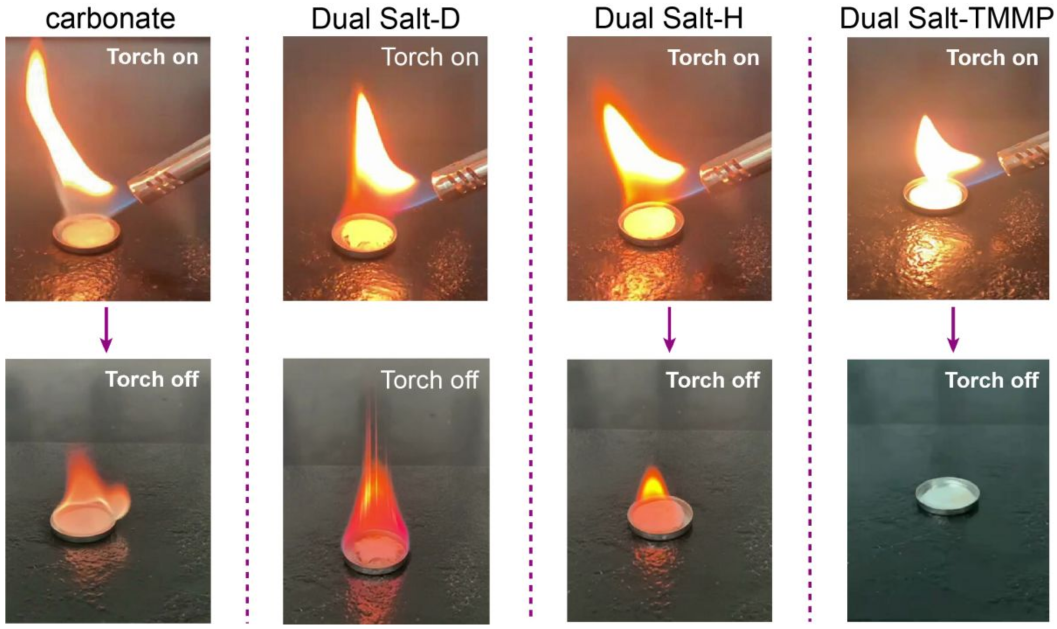

Figure S47. The flammability tests of different electrolytes: carbonate, Dual Salt-D, Dual Salt-H, and Dual Salt-TMMP.

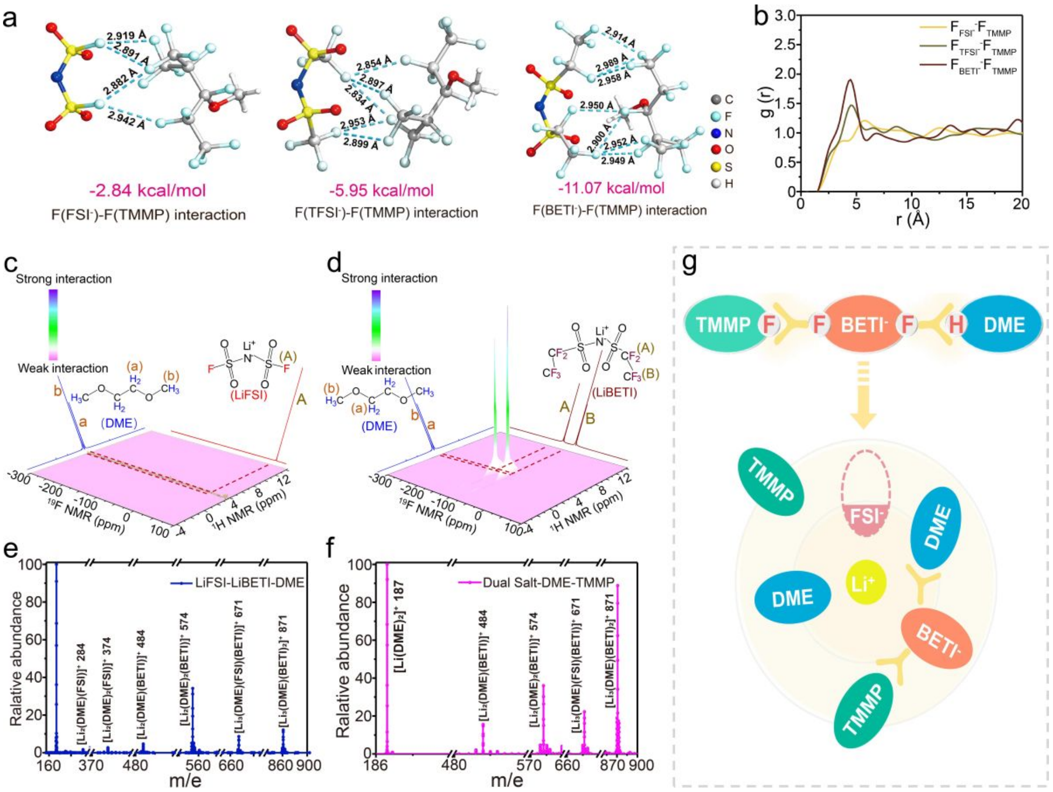

Figure 2. (a) DFT calculations of fluorophilic interactions between different anions and TMMP. (b) Radial distribution functions g(r) of F_{FSI}--F_{TMMP}, F_{TFSI}--F_{TMMP}, and F_{BETI}--F_{TMMP}. The ^{19}F-^{1}H HOESY NMR spectrum of (c) LiFSI-DME mixture and (d) LiBETI-DME mixture. ESI-MS characterizations of (e) LiFSI-LiBETI-DME mixture, and (f) Dual Salt-TMMP. (g) Solvation structure of Dual Salt-TMMP electrolyte.

Reviewer #2:

Chen et al. reported the rational design of safe and high-efficiency ether-based electrolytes for high-voltage lithium metal batteries. By introducing a flame-retardant diluent with an ultrahigh F/H atomic ratio of 4.33 and higher, and employing molecularly engineered anions bearing fluoro-alkyl moieties, they effectively addressed the immiscibility issue of highly fluorinated diluents through synergistic fluorophilic (F···F) and hydrogen-bonding (F···H) interactions. The resulting fluorous electrolytes enabled dendrite-free Li plating/stripping with a high Coulombic efficiency of ~99.5% and long-term cycling stability of Ni-rich NMC811 cathodes. Overall, this study is interesting and the results are promising. Its publication will be of great value to the battery research community. I recommend publication after minor revision, and my specific comments are as follows.

Response: We sincerely thank the reviewer for the positive and insightful comments on our work. We have carefully addressed each of the reviewer's valuable suggestions point by point, and corresponding revisions have been made to improve the quality and clarity of the manuscript.

1. Why is a clear position shown in Figure 1a for TMMP if its flash point cannot be quantified? Why is the flash point of TMMP unmeasurable?

Response: We thank the reviewer for this insightful question. Regarding your first concern, TMMP indeed has no quantifiable flash point up to the temperature limit of the tester (200 ^{o}C), so it should not be noted at a clear position in **Figure 1a**. However, TMMP plays the key role in this study, and it would be better to include it in the figure along with the other diluents. Therefore, we placed TMMP at the position of its corresponding F/H ratio, outlined it with a dashed box and labeled it with the text 'Not measurable flash point' to avoid misunderstanding.

Regarding your second concern, flash point is the minimum temperature at which a liquid forms a vapor above its surface in sufficient concentration that it can be ignited. In this work, we use closed-cup testing to measure the flash point of TMMP, but even when it reached its boiling point (97.8 ^{o}C), we were still unable to ascertain its flash

point. This behavior can be attributed to the intrinsic flame-retardant nature of TMMP. Specifically, the high fluorine content effectively suppresses combustion by quenching the chain reactions of free radicals and reducing the heat release necessary to sustain burning. In order to comprehensively demonstrate the flame-retardant properties of TMMP, the ignition experiments and DSC tests were carried out to complement the flash point testing in this work.

2. This study shows that the amphiphilic $BETI^-$ anion has strong interactions with the TMMP diluent. This finding seems to challenge the traditional view of diluents as purely inert, non-coordinating components in LHCEs. Does the introduction of this "non-inert" diluent, which interacts strongly with the anion, ultimately perturb the inner solvation structure of the Li^+ ion?

Response: We thank the reviewer for this insightful question. Notably, previous works (e.g., by Fan et al. *Chem*, **2023**, 9 (3), 650-664) have already demonstrated that diluents in LHCE may engage in non-trivial interactions with anions rather than acting as purely inert "spectator" components [**Ref. 1**]. In our present study, as illustrated in **Figure 2g**, we indeed observe that the amphiphilic anion $BETI^-$ exhibits noticeable interactions with the diluent TMMP. However, these interactions occur outside the primary solvation sheath of Li^+. The inner solvation shell of Li^+ is mainly composed of solvent (DME) molecules and anions ($BETI^-$ or FSI^-), while TMMP molecules remain in the outer solvation domain, where they interact weakly with $BETI^-$ through dipole-anion and van der Waals interactions.

Such outer-sphere interactions do not perturb the Li^+-solvent coordination structure, as no TMMP-derived Li-O or Li-F coordination features are detected. Instead, TMMP modulates the anion distribution in the outer layer, suppressing anion aggregation and enhancing the homogeneity of the electrolyte microenvironment. Therefore, while TMMP is not completely "inert," its influence is confined to the outer solvation region, maintaining the Li^+ inner solvation motif while improving viscosity, wettability, and safety.

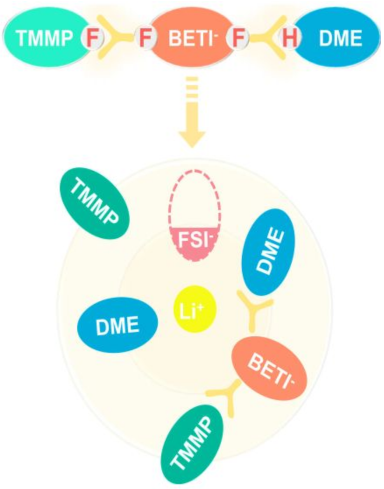

Figure 2g. Solvation structure of Dual Salt-TMMP electrolyte.

3. The manuscript presents NMR data as key evidence for specific molecular interactions. However, the molecules involved in this article contain multiple hydrogen and fluorine atoms in distinct chemical environments. To better enhance the clarity and persuasiveness of the data, the authors should provide detailed signal assignments in the corresponding NMR spectra.

Response: We thank the reviewer for this valuable suggestion. In response, we have carefully revised the NMR data presentation. The detailed signal assignments for both ^{1}H and ^{19}F NMR spectra have now been clearly provided in **Figures 2c-2d, Figure S4, and Figure S6**. These revisions help clarify the distinct chemical environments of hydrogen and fluorine atoms and improve the overall clarity and persuasiveness of the NMR analysis.

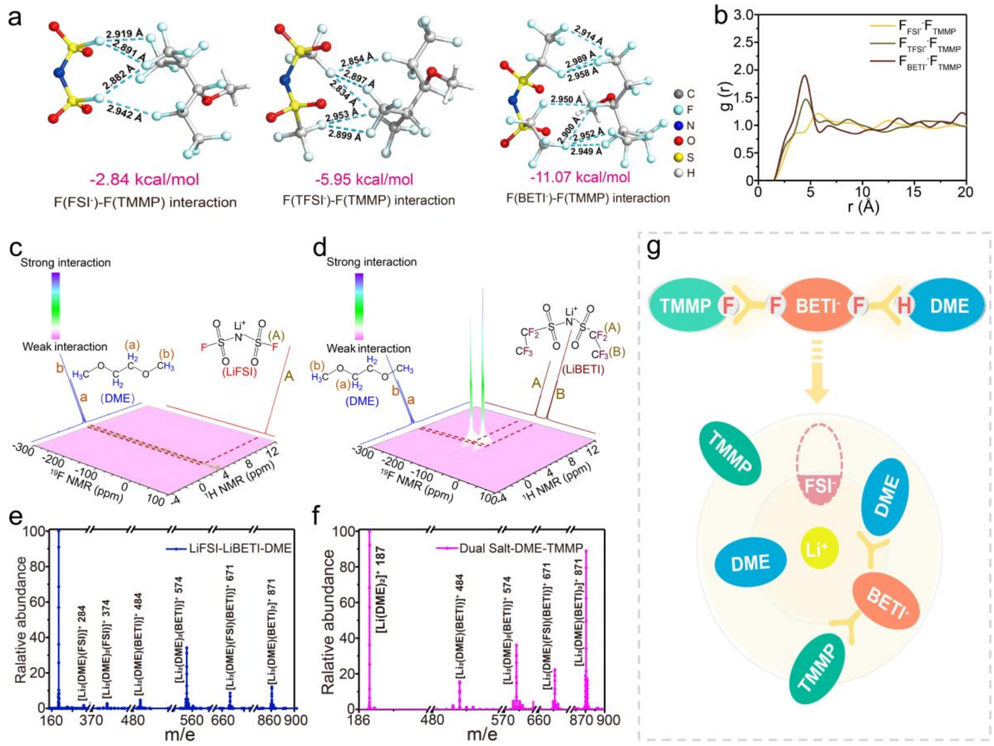

Figure 2. (a) DFT calculations of fluorophilic interactions between different anions and TMMP. (b) Radial distribution functions g(r) of F_{FSI}--F_{TMMP}, F_{TFSI}--F_{TMMP}, and F_{BETI}--F_{TMMP}. The ^{19}F-^{1}H HOESY NMR spectrum of (c) LiFSI-DME mixture and (d) LiBETI-DME mixture. ESI-MS characterizations of (e) LiFSI-LiBETI-DME mixture, and (f) Dual Salt-TMMP. (g) Solvation structure of Dual Salt-TMMP electrolyte.

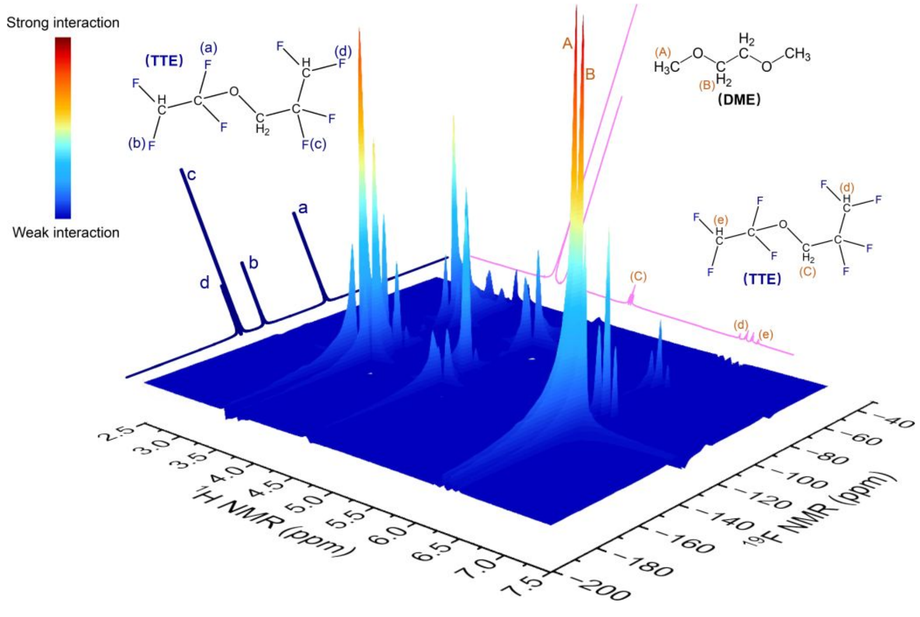

Figure S4. The ^{19}F-^{1}H HOESY NMR spectrum of DME-TTE mixture.

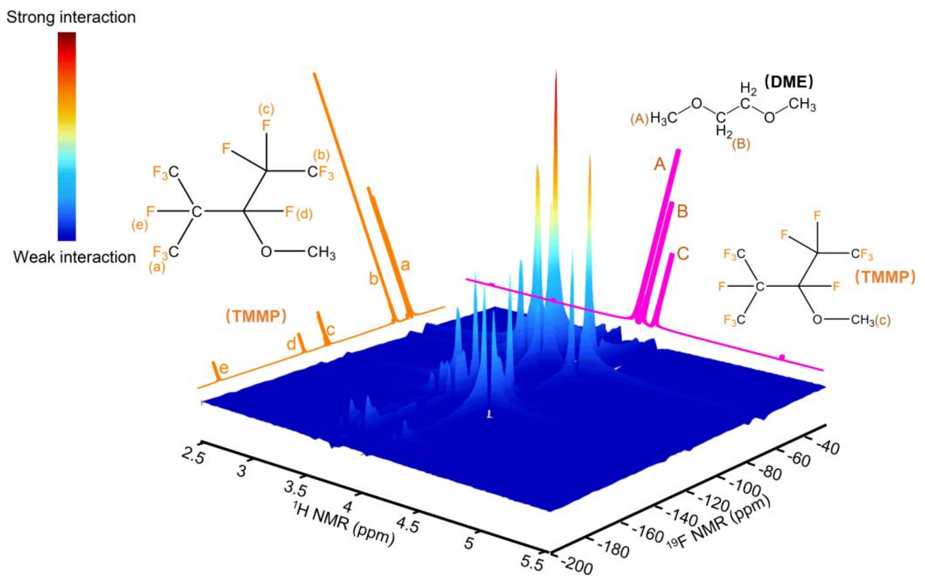

Figure S6. The ^{19}F-^{1}H HOESY NMR spectrum of DME-TMMP mixture.

4. Units and symbols are unclear or missing; for example, "ionic conductivity is 2.12 at 25 °C" lacks units (should be mS·cm^{-1} or S·m^{-1}), and viscosity reported in "cp" should also include the SI unit (mPa·s).

Response: We thank the reviewer for this kind suggestion. All units and symbols have been carefully checked and corrected throughout the manuscript to ensure consistency and clarity. Specifically, the ionic conductivity values are now reported with units of mS·cm^{-1}, and the viscosity values in cP have been converted and uniformly expressed in mPa·s in accordance with SI conventions.

Changes in the manuscript:

A paragraph has been added on Pages 6 to elaborate on this point:

- "As shown in Table S3, the ionic conductivity of Dual Salt-TMMP is 2.12 mS·cm^{-1} at 25 °C, which is higher than that of Dual Salt-H (1.95 mS·cm^{-1})."
- "The improved Li deposition kinetics of Dual Salt-TMMP may be attributed to its lower viscosity (12.65 mPa·s) compared to Dual Salt-H (32.23 mPa·s), as well as its superior wettability on both the Celgard 2500 separator and Li foil (Figure S19)."

5. The discussion of the dual-salt strategy lacks consideration of cost, scalability, and environmental impact. Highly fluorinated compounds (e.g., BETI, TMMP, CFH) are

costly and raise environmental and toxicity concerns. The authors should briefly address these aspects or note the associated limitations.

Response: We sincerely thank the reviewer for this valuable comment. We fully agree that cost, scalability, and environmental impact are important considerations for the practical application of dual-salt electrolytes. Highly fluorinated components such as $BETI^-$, TMMP, and CFH may introduce challenges related to synthesis cost and sustainability. In response to the reviewer's suggestion, we have added a brief discussion in the "*Conclusion*" section to acknowledge these aspects and to highlight that future work will aim to explore less-fluorinated or fluorine-free systems with similar solvation and interfacial properties.

Changes in the manuscript:

A paragraph has been added on Pages 6 to elaborate on this point:

"In conclusion, this study presents a novel approach to addressing the safety and performance challenges in high-energy-density lithium metal batteries through the development of miscible fluorous electrolytes enabled by amphiphilic anion chemistry. By employing anions with fluoro-alkyl moieties, specifically $BETI^-$, we successfully bridged the gap between Li^+-solvating solvents and highly fluorinated diluents, resolving the critical issue of immiscibility in fluorous electrolytes. The Dual Salt-TMMP electrolyte exhibits remarkable lithium metal reversibility, achieving a high CE of 99.5%, and enables stable cycling of Li||NCM811 cells with 87% capacity retention after 200 cycles at 4.4 V, facilitated by LiF-rich electrode-electrolyte interphases. It also shows enhanced thermal stability and flame retardancy, addressing key safety concerns. This amphiphilic anion approach provides new opportunities for tailoring electrolyte compositions to meet the demands of next-generation energy storage systems. Looking ahead, rational anion design can further improve miscibility and electrochemical performance. Beyond fully fluorinated anions like $BETI^-$, amphiphilic or partially fluorinated motifs can balance polarity and fluorophilicity, regulate Li^+ coordination, and enhance compatibility with solvents such as TMMP. Although highly fluorinated components are beneficial for electrochemical stability and battery safety,

they are often associated with high cost and significant environmental concerns. Therefore, future efforts should focus on developing green recycling strategies, designing degradable fluorinated species, or exploring fluorine-free molecular alternatives to achieve sustainable electrolyte systems for safe and high-energy batteries."

6. There are grammatical errors and misspellings in the abstract and main text, such as "impatability", and the authors are advised to carefully proofread and thoroughly revise the grammar throughout the manuscript.

Response: We thank the reviewer for pointing this out. The manuscript has been carefully proofread, and all grammatical errors, misspellings (including "impatability"), and language issues throughout the manuscript have been corrected to improve clarity and readability.

7. Why were different CD rates chosen in Figure 3e?

Response: We thank the reviewer for the question. In practical pouch-cell tests of Li metal batteries, fast-charging conditions often lead to severe dendrite growth and rapid cell failure. Therefore, a relatively lower charging rate was adopted to mitigate these issues. This approach is also consistent with common practices in current Li metal pouch-cell evaluations [**Refs. 1-2**].

[**Ref. 1**] Liu, L.; Xiang, Y.X.; Wang, J.H. From cell to atomic level: understanding the degradation in 99% coulombic efficiency and 450 Wh kg^{-1} anode-free pouch cells. *J. Am. Chem. Soc.* **2025**, 147, 41342-41354.

[**Ref. 2**] Liu, X. X.; Li, Y.; Liu, J. D.; Wang, H. P.; Zhuang, X. J.; Ma, J. M. 570 Wh kg^{-1}-grade lithium metal pouch cell with 4.9 V highly Li^{+} conductive armor-like cathode electrolyte interphase via partially fluorinated electrolyte engineering. *Adv. Mater.* **2024**, 36, 2401505.

8. It is recommended to provide the quantitative analysis results of XPS. In addition, NMC811 is commonly written as NCM811.

Response: We thank the reviewer for the helpful suggestion. The quantitative analysis results of XPS have been added to the revised manuscript to provide more detailed information. In addition, all instances of "NMC811" have been corrected to the commonly used notation "NCM811" throughout the manuscript.

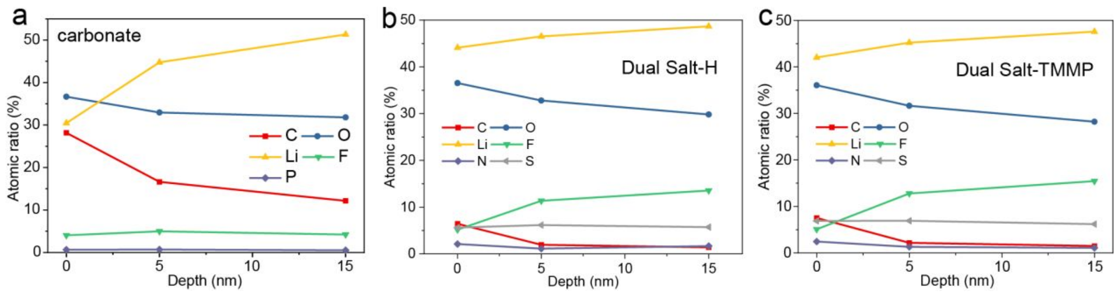

Figure S43. Atomic ratios during sputtering for Li anodes cycled in (a) carbonate, (b) Dual Salt-H, and (c) Dual Salt-TMMP electrolyte.

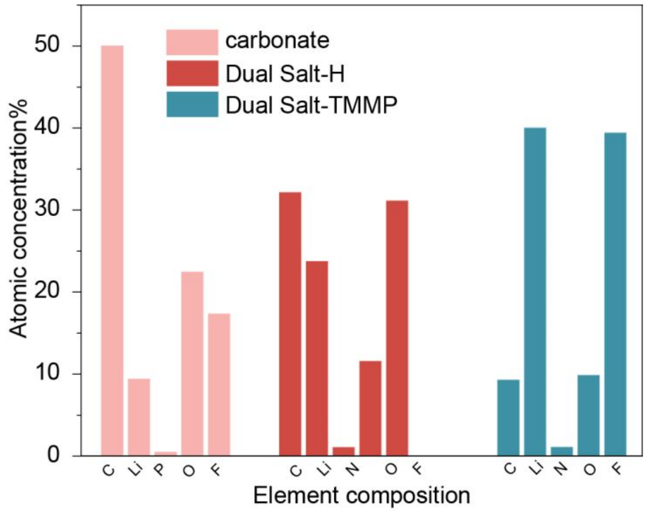

Figure S48. Atomic ratios during sputtering for NCM811 cycled in various electrolytes.

Reviewer #3:

The manuscript designs "amphiphilic" anions to compatibilize highly fluorinated diluents with ether solvents and salts, aiming to deliver non-flammable, Li-metal-compatible electrolytes. The core formulation is a dual-salt ether electrolyte in which LiBETI is introduced alongside LiFSI to stabilize a DME plus TMMP diluent mixture that would otherwise phase-separate. The study combines ITC, multinuclear NMR including HOESY, ESI-MS speciation, short classical MD, small-cluster DFT, flammability tests, Li‖Cu CE by the Aurbach protocol, Li‖Li cycling, Li‖NMC811 coin and pouch cells, and a thermal abuse test with external heating. Performance trends are favorable versus the chosen internal controls and the fire behavior is clearly improved.

Response: We thank the reviewer for the careful summary of our work and for recognizing the key aspects of our study. We are pleased that the design of amphiphilic anions, the dual-salt strategy, and the combination of experimental and computational techniques are clearly understood. As noted, the dual-salt ether electrolyte effectively stabilizes the DME/TMMP diluent mixture and improves both electrochemical performance and fire safety.

1. Mechanistic evidence versus strength of methods

The central mechanism is that $BETI^-$ simultaneously engages in F···F interactions with the fluorous diluent and F···H interactions with the ether, bridging immiscible components. The experimental indicators are ITC sign changes, small chemical-shift movements, HOESY cross-peaks, and ESI-MS adducts, while calculations use 0 K gas-phase DFT of dimers and very short MD with a generic non-polarizable force field. These signals are suggestive, not decisive.

Response: We thank the Reviewer for this important comment. We fully agree that each single probe (ITC sign changes, small NMR shifts, HOESY cross-peaks, and ESI-MS adducts) is suggestive rather than conclusive by itself. We therefore revised the manuscript to temper our wording and explicitly discuss method limitations. Importantly, the independent techniques converge on a consistent interaction picture: calorimetry indicates a reorganization of the solvation/interaction energetics, NMR-

HOESY shows spatial proximity of BETI⁻ to both fluorous and etheric moieties, and ESI-MS reveals persistent ion–diluent associations under gentle ionization conditions. Together these observations provide a compelling, cross-validated case that BETI⁻ can simultaneously interact with fluorous and ether domains. We also clarified in the revised Supporting Information that the DFT and short classical MD runs were used to illustrate feasible local minima and binding geometries rather than to provide fully converged thermodynamic quantities.

These experimental observations are mutually reinforcing. ITC measurements indicate an enthalpic inversion associated with reorganization of the TMMP and DME solvation environments. NMR spectroscopy, particularly HOESY, reveals spatial proximities between the anion and both diluent and ether hydrogen sites, consistent with the proposed dual-interaction mode. We note that gas-phase adducts may not fully represent the solution ensemble; nonetheless they corroborate a propensity for ion–diluent association under gentle ionization.

On the computational side, we acknowledge that 0 K gas-phase DFT calculations and short-timescale classical MD simulations cannot provide fully converged thermodynamic data. These calculations are intended to identify representative local minima and feasible binding geometries, serving as qualitative support to visualize how BETI⁻ may act as a molecular bridge between otherwise immiscible components.

Taken together, this multi-pronged strategy, integrating calorimetry, NMR spectroscopy, mass spectrometry, and theory, provides compelling mechanistic support for the unique amphiphilic role of BETI⁻ in structuring LHCEs. Compared with previous studies that relied on single-probe characterizations or bulk thermodynamic observations, our integrated spectroscopic-calorimetric-computational framework offers a methodologically innovative and mechanistically grounded understanding of ion-diluent interactions, bridging molecular-level insights with macroscopic electrolyte behavior.

a) HOESY cross-peaks demonstrate proximity, not bond character or directionality. Please report mixing times, distance calibration, and control spectra that exclude nuclear Overhauser artifacts from spin diffusion.

Response: We thank the reviewer for the insightful comment regarding the interpretation of the HOESY cross-peaks. We fully agree that HOESY mainly provides information about spatial proximity rather than direct bond connectivity or directionality. To prevent any potential misunderstanding, we have carefully revised the relevant statements in the manuscript to clarify this point.

In our experiments, the following NMR parameters were used: measurements were performed on a JNM-ECZ600R/S1 spectrometer at a field strength of 14.09637 T (600 MHz for ^{1}H) at 298 K, using a ^{1}H-observed/ ^{19}F-irradiated HOESY pulse sequence. The mixing time was fixed at 1 s, which is consistent with established practice in qualitative HOESY measurements. As demonstrated across multiple studies, small-molecule ^{19}F-^{1}H HOESY experiments routinely employ mixing times in the range of 0.5-1 s, reflecting the characteristic T_1 values of protons and fluorine nuclei in such systems (*Organometallics* **1992**, *11*, 3566-3570; *J. Am. Chem. Soc.* **1995**, *117*, 10405-10406; *J. Phys. Chem. B* **2015**, *119*, 9225-9235; *Magn. Reson. Chem.* **2006**, *44*, 76-82). The use of a 1 s mixing time therefore lies well within the standard experimental window and provides reliable qualitative information on spatial proximity. The acquisition matrix consisted of 1024 points for ^{1}H and 128 points for ^{19}F, with 4 scans on the ^{1}H dimension and no scans on the ^{19}F dimension. Spectral widths and resolutions were 11.28159 kHz / 11.01718 Hz for ^{1}H and 227.27273 kHz / 1.77557 kHz for ^{19}F, with offsets of 5 ppm (^{1}H) and -100 ppm (^{19}F).

Regarding distance calibration, we note that a quantitative NOE-distance calibration was not performed because the aim of our HOESY experiment was basically to qualitatively confirm the presence of spatial proximity between specific ^{1}H and ^{19}F nuclei, rather than to extract accurate internuclear distances. For heteronuclear HOESY experiments used solely for qualitative assignment or interaction identification, the absence of distance calibration is standard practice. We have clarified this point in the revised manuscript. Furthermore, our discussion of the interactions does not rely solely

on HOESY data. Instead, it is supported by complementary analyses, including ^{1}H NMR, Nano ITC, and ESI-MS, which together provide chemical shift information, thermodynamic parameters, and stoichiometric evidence, respectively. The integration of these multi-technique results ensures that our conclusions are self-consistent and robust, while minimizing potential artifacts from spin diffusion in HOESY.

We believe that this multi-technique approach provides a thorough and reliable basis for the interpretations presented in the manuscript, ensuring that the conclusions are supported from structural, thermodynamic, and solution-phase perspectives. We sincerely appreciate the reviewer's suggestions and will continue to refine our HOESY methodology, including more extensive mixing-time-dependent controls, in future studies.

Changes in the Supporting Information:

A paragraph has been added on **Page 4** to elaborate on this point:

"To qualitatively examine the spatial proximity between solvent and anion molecules in the inner solvation sheath, two-dimensional ^{19}F-^{1}H HOESY NMR spectra were recorded. The analysis revealed a striking contrast between the LiFSI-DME and LiBETI-DME mixtures (Figures 2c-2d). The LiFSI-DME mixture showed no significant HOESY signal, indicating negligible spatial correlation between the FSI^- anion and DME solvent molecules (Figure 2c). In contrast, the LiBETI-DME mixture exhibited a distinct HOESY signal, providing clear evidence of a close spatial association between the $BETI^-$ anion and DME molecules (Figure 2d). This observation has important implications for our electrolyte design. The strong interaction between $BETI^-$ and DME suggests that amphiphilic $BETI^-$ plays a crucial role in organizing the solvation structure. When TMMP is introduced as a diluent, $BETI^-$ can effectively bridge the gap between the solvent (DME) and the highly fluorinated diluent (TMMP), facilitating the formation of a stable, miscible electrolyte."

Changes in the manuscript:

A paragraph has been added on Page 2 to elaborate on this point:

"HOESY experiments were performed on a JNM-ECZ600R/S1 spectrometer at 14.09637 T (600 MHz for ^{1}H) and 298 K, using a ^{1}H-observed/ ^{19}F-irradiated HOESY pulse sequence. The mixing time was fixed at 1 s, which is a commonly adopted parameter in many small-molecule HOESY studies. The acquisition matrix comprised 1024 points for 1H and 128 points for ^{19}F, with 4 scans on the ^{1}H dimension and no scans on the ^{19}F dimension. Spectral widths and digital resolutions were 11.28159 kHz/ 11.01718 Hz for ^{1}H and 227.27273 kHz/ 1.77557 kHz for ^{19}F, with offsets of 5 ppm (^{1}H) and -100 ppm (^{19}F)."

b) ESI-MS is prone to gas-phase rearrangement of weak associates. A solution-phase quantification of association constants by VT-NMR, Raman band-shape analysis with constrained deconvolution, or diffusion-ordered NMR would be more convincing.

Response: We thank the reviewer for the valuable suggestion regarding solution-phase quantification methods such as VT-NMR, Raman band-shape analysis, or DOSY-NMR. These techniques indeed provide complementary information on association constants.

In our study, ESI-MS was chosen because it is highly sensitive to weak and transient complexes, allowing us to detect species that might be challenging to observe by solution-phase methods. To minimize potential gas-phase rearrangements, the ESI-MS experiments were performed under gentle ionization conditions (e.g., low spray voltage). Previous studies have shown that under such conditions, ESI-MS can reliably reflect solution-phase associations [**Refs. 1-5**].

We note that solution-phase methods also have inherent limitations, especially for probing the structure of complex electrolyte solutions. VT-NMR, while capable of providing temperature-dependent association information, often requires relatively high solute concentrations and long equilibration times, which can perturb the delicate equilibrium in low-concentration electrolyte systems. Raman band-shape analysis, although useful for detecting specific solvation interactions, relies on constrained deconvolution and is highly sensitive to overlapping bands and background solvent signals, making quantitative interpretation challenging in multicomponent electrolytes. DOSY-NMR provides diffusion-based separation, but its resolution is limited when

species have similar diffusion coefficients, which is often the case for small solvent molecules and weakly associated complexes. Therefore, no single solution-phase method is fully capable of capturing all aspects of electrolyte solvation and association behavior in our system.

By carefully optimizing ionization conditions, gas-phase artifacts are minimized, ensuring that the observed species reflect true solution-phase tendencies. Therefore, while no single technique is perfect, **ESI-MS arguably the most practical and informative method** for dissecting weak yet chemically important anion–solvent interactions in our electrolytes. Our results, together with complementary analyses, robustly support the conclusions regarding anion-solvent coordination.

[**Ref. 1**] Gireaud, L.; Grugeon, S.; Pilard, S.; Guenot, P.; Tarascon, J.M.; Laruelle, S. Mass spectrometry investigations on electrolyte degradation products for the development of nanocomposite electrodes in lithium ion batteries. *Anal. Chem.* **2006**, 78 (11), 3688-3698.

[**Ref. 2**] Matsuda, Y.; Fukushima, T.; Hashimoto, H.; Arakawa, R. Solvation of lithium ions in mixed organic electrolyte solutions by electrospray ionization mass spectroscopy. *J. Electrochem. Soc.* **2002**, 149 (8), A1045.

[**Ref. 3**] Ren, X. Y.; Dou, R. J.; Wang, Q.; Hu, K. X.; Su, K. H.; Liu, C.; Lu, L. H. A biocompatible deep eutectic electrolyte enables ultra-fast charging in lithium-ion batteries. *Adv. Funct. Mater.* **2025**: 2500464.

[**Ref. 4**] Hu, J.; Wang, T.; Zhang, W.J. et al. Dissecting the flash chemistry of electrogenerated reactive intermediates by microdroplet fusion mass spectrometry. *Angew. Chem. Int. Edit.* **2021**, 60 (34), 18494-18498.

[**Ref. 5**] Chen, J. X.; Wang, X. Y.; Cui, X.; Li, Y. Y.; Feng, Y. Q.; Wei, Z. W. In situ probing and identification of electrochemical reaction intermediates by floating electrolytic electrospray mass spectrometry. *Angew. Chem. Int. Edit.* **2023**, 135 (12), e202219302.

c) The MD windows are far too short to characterize mixing thermodynamics and outer-shell organization. Provide longer trajectories with a validated force field, density checks, and Kirkwood-Buff integrals or PMFs that directly show the proposed F-rich co-ordination to $BETI^-$.

Response: We sincerely thank the reviewer for this valuable comment. We realize that our original description of the molecular dynamics (MD) simulations in the Methods section was not sufficiently clear. In fact, two types of simulations were performed in this work: classical MD and ab initio molecular dynamics (AIMD). We have now revised the computational details to clearly distinguish between them. This revision clarifies that the 50 ps trajectory refers to the AIMD simulations, while the classical MD simulations were conducted with 2 ns total simulation time (1.0 ns for equilibration and 1.0 ns for production). The AIMD simulations were mainly used to verify the local coordination environment at the molecular level, while the classical MD simulations provided statistical information on the overall coordination and dynamic behavior. We have carefully rewritten this section in the revised manuscript for clarity.

Changes in the supporting information:

A paragraph has been added on **Page 6** to elaborate on this point:

"The molecular dynamics (MD) simulations were also used to characterize the coordination in the studied ether-based electrolytes.[6, 7] The classical MD simulations were performed using the Forcite module in Materials Studio with the COMPASS III force field. A charge-scaling factor of 0.7 was applied to both the cation (Li^+) and anions. The amorphous cell was constructed as a cubic cell with a side length of approximately 30 Å, into which the electrolyte components were packed according to the desired stoichiometry. After geometry optimization and annealing, the systems were first equilibrated for 1.0 ns in the isothermal-isobaric (NPT) ensemble at 303 K, followed by a 1.0 ns production run in the canonical (NVT) ensemble. The mean-squared displacement (MSD) and radial distribution functions (RDFs) were obtained from the converged portion of the production trajectories. Ab initio molecular dynamics (AIMD) simulations were performed using the Vienna Ab initio Simulation Package

(VASP) with projector-augmented wave (PAW) potentials and the Perdew-Burke-Ernzerhof (PBE) generalized gradient approximation (GGA). The simulations were conducted for 50 ps at 300 K in the NVT ensemble using a Nosé thermostat (damping parameter 2.0) and a 0.5 fs time step."

d) The DFT dimer binding energies are small in magnitude and lack a solvation thermodynamic cycle. Present these as qualitative trends only and avoid quantitative causality.

Response: We thank the reviewer for the suggestion regarding the solvation thermodynamic cycle. Constructing a complete thermodynamic cycle would require explicit consideration of solvation free energies, entropic contributions, and temperature/pressure effects, which is computationally very demanding for the complex molecular systems studied here and beyond the scope of the current work.

In this study, we used DFT (e.g., B3LYP/6-311++G(d,p)) to optimize the geometries of selected dimers and compute their binding energies in the gas phase. These computed energies do **not** include explicit solvation or entropic/thermal corrections and should therefore be interpreted as **qualitative trends** of interaction strengths rather than absolute free energies.

Such qualitative DFT analysis of weak intermolecular interactions are widely adopted in the literature (e.g., *Nat. Chem.* **2024**, *16*, 1427-1435; *Angew. Chem. Int. Ed.* **2021**, *60*, 22683-22687; *Nat. Commun.* **2024**, *15*, 1206). Importantly, the calculated F···F interaction energies are consistent with previously reported magnitudes for halogen-halogen or fluorine-fluorine contacts in molecular clusters and ionic systems (see, e.g., *Cryst. Growth Des.* **2020**, *20*, 2943-2951; *J. Phys. Chem. A* **2012**, *116*, 1435-1444; *Chem. Soc. Rev.* **2005**, *34*, 22-30; *Phys. Chem. Chem. Phys.* **2016**, *18*, 20381-20388.). These comparisons indicate that the DFT-derived interaction trends presented here are physically reasonable and in good agreement with literature precedents.

Changes in the manuscript:

A paragraph has been added on Pages 3 to elaborate on this point:

"The number of C-F bonds in anions significantly influences their fluorophilic interactions with TMMP. Among the studied anions (FSI^-, $TFSI^-$, and $BETI^-$), DFT calculations indicate a qualitative trend of increasing fluorophilic interactions in the order $FSI^- < TFSI^- < BETI^-$ (Figure 2a). These computed binding energies are small in magnitude and do not account for explicit solvation or entropic effects, and thus should be interpreted as relative trends rather than absolute interaction strengths. Consistently, MD simulations also reflect this trend, supporting the relative ordering of fluorophilic interactions (Figure 2b)."

2. Transport numbers and conductivity need statistics and cross-checks

Conductivity differences between the new and control electrolytes are modest, and the reported Li transference relies on self-diffusion ratios or Bruce-Vincent polarization which are method-dependent. Please report n for each metric, mean ± SD, fitting residuals for VTF and EIS, and add a complementary transference method such as restricted diffusion or electrophoretic NMR. Where σ_{Li+} is discussed, propagate uncertainties rather than quoting single values.

Response: We thank the reviewer for the valuable comments. The ionic conductivity measurements were not performed using coin cells, but with custom-made molds, which provide higher accuracy. For the two electrolytes at 25 °C, each measurement was independently conducted three times. The reported values are the mean of the three measurements: for Dual Salt-H, the values are 1.38, 1.35, and 1.37 mS cm^{-1}, and for Dual Salt-TMMP, the values are 7.18, 7.10, and 7.25 mS cm^{-1}. According to the reviewer's suggestion, we have calculated the mean ± standard deviation (mean ± SD) and updated the corresponding values in **Table S3**. As this work focuses on a fixed temperature of 25 °C, we did not study the temperature dependence of ionic conductivity, and therefore VTF fitting residuals were not provided. The mean ± SD at a fixed temperature provides a direct and transparent representation of data reliability.

Table S3. The physical properties of the studied electrolytes (25 °C)

Electrolytes	Ionic conductivity (mS cm^{-1})	Ion mobility number (t_{Li})	Diffusion coefficient (D_{Li}) (m^2/s)
Dual Salt-H	1.38 ± 0.03	0.53	1.36×10^{-11}
Dual Salt-TMMP	7.18 ± 0.08	0.56	6.98×10^{-11}

Note: The ionic conductivity (σ) of each electrolyte was measured three times independently at a fixed temperature of 25 °C using a calibrated conductivity meter. The reported values are presented as mean ± standard deviation (mean ± SD) to reflect experimental reproducibility.

Regarding your other comment on the Li^+ transference number (t_{Li+}), the values reported in the manuscript were determined using the PFG-NMR method, which directly measures self-diffusion coefficients of the ions and is widely recognized for its accuracy in evaluating ion transport in liquid electrolytes. To further verify the reliability of these measurements and address the reviewer's concern, we performed cross-validation using Li||Li symmetric cells following the Bruce–Vincent method (**Figure R1**). This complementary technique provides an independent evaluation of Li^+ transport behavior and enables direct comparison with the PFG-NMR results. The calculated Li^+ transference numbers are 0.52 for Dual Salt-H and 0.63 for Dual Salt-TMMP, which are in close agreement with the corresponding PFG-NMR values. This consistency strongly supports the reliability of our measurements and confirms that the observed trends in Li^+ mobility are intrinsic to the electrolyte systems rather than artifacts of a specific method. The cross-validation confirms that the originally reported t_{Li+} values are accurate and consistent, supporting the conclusions regarding the ion transport characteristics of the two electrolytes in this study.

We hope these additions and clarifications satisfactorily address the reviewer's concerns regarding method dependence and statistical rigor.

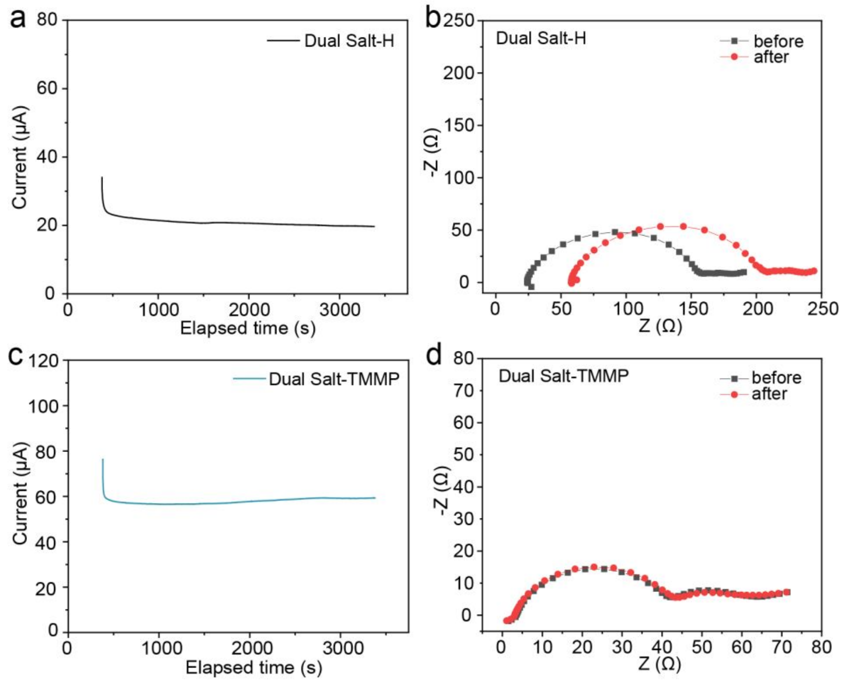

Figure R1. Determination of t_{Li+} using the Bruce-Vincent method. Panels (a) and (c) show the polarization current profiles of Li||Li symmetric cells using the Dual Salt-H and Dual Salt-TMMP electrolytes, respectively, under a constant DC voltage of 5 mV. Panels (b) and (d) display the Nyquist plots obtained through EIS before and after polarization for each corresponding electrolyte system. Measurements were conducted with an AC amplitude of 10 mV over a frequency range of 1 MHz to 100 mHz.

3. Electrochemical stringency and practicality

Li||Cu CE values near 99.5 percent at 0.5 to 2 mA cm^{-2} are promising but depend sensitively on protocol details. State plating/stripping capacity per cycle, rest times, electrolyte volume per area, stack pressure, and the number of repeats. For Li||Li and Li||NMC811, provide areal loadings, N/P, and E/C ratios for every data set, include error bars across independent cells, and show post-mortem morphology to support claims of "dendrite-free" deposition. The pouch cell result is interesting; please include replicates and confidence intervals.

Response: We thank the reviewer for the constructive suggestions. We have addressed all points as follows:

For the **Li||Cu CE measurements**, detailed electrochemical parameters, including

plating/stripping capacity per cycle, rest time, electrolyte volume per area, stack pressure, and the number of repeats, are now provided in the **Methods** section.

Experimental Details (Aurbach method, partial plating/stripping):

The cells were allowed to rest for 2 hours before cycling. A Cu foil (2.11 cm^2) was used as the working electrode. Plating and stripping were carried out at current densities of 0.5, 1.0, and 2.0 mA cm^{-2} (corresponding to 1.05, 2.11, and 4.22 mA, respectively), with a full plating/stripping capacity of 5.28 mAh per cycle. For the partial plating/stripping protocol, a capacity of 1.05 mAh per cycle was applied for 10 cycles. The electrolyte volume-to-area ratio was 35.54 μL cm^{-2}, and the stack pressure was maintained at 850 psi. Data were recorded every 10 s, and two parallel cells were tested for reproducibility.

Experimental Details (Full stripping):

The cells were allowed to rest for 2 hours before cycling. A Cu foil with an areal area of 2.11 cm^2 was used as the working electrode. Plating and stripping were conducted at a current of 1.055 mA (0.5 mA cm^{-2}), with a full plating/stripping capacity of 2.11 mAh per cycle. The electrolyte volume-to-area ratio was 35.54 μL cm^{-2}, and a stack pressure of 850 psi was applied during cycling. Data were recorded every 10 s, and two parallel cells were tested to ensure reproducibility.

For **Li||Li** and **Li||NCM811** measurements, the requested parameters have now been added in the Methods section:

- **Li||Li:** Areal loading of Li: 1.0 mAh cm^{-2}; current density: 0.5 mA cm^{-2}; electrolyte volume per area: 35.54 μL cm^{-2}; E/C ratio: 75 μL mAh^{-1}; stack pressure: 850 psi; rest time: 2 hours; number of parallel cells: 2.
- **Li||NCM811**: Areal loading of NCM811: 10 mg ± 0.4 mg cm^{-2}; E/C ratio: 133 mWh mL^{-1}; stack pressure: 850 psi; rest time: 2 hours; number of parallel cells: 2.

It should be noted that the Li metal used in the Li||NCM811 cells is a thick foil (≈450 μm), corresponding to an areal capacity far exceeding that of the cathode. Therefore, the Li anode is in significant excess, and the N/P ratio is not explicitly defined in this configuration. This design ensures that the observed electrochemical behavior primarily

reflects the cathode utilization and electrolyte stability rather than Li depletion effects.

Regarding the cycling performance of Li||NCM811 with the Dual Salt-TMMP electrolyte, two parallel experiments were conducted, showing capacity retention of ~85% and ~87% after 200 cycles, resulting in an average of ~86%. The CE was 99.7% in both parallel experiments. While only two replicates were performed due to resource limitations, the results are highly consistent and clearly demonstrate greatly improved cycling stability. To reflect this, we have updated **Figure 3d** and **S36** to include the two parallel datasets for the Dual Salt-TMMP electrolyte, highlighting their consistency. We agree with the reviewer that additional replicates and statistical error bars would further strengthen the quantitative analysis, and we plan to address this in future work.

The pouch cell details and corresponding data have been summarized in **Table S5**. Although each dataset currently includes two parallel cells due to resource limitations, the results are consistent and sufficient to support the reported trends. We plan to increase the number of replicates and include more comprehensive post-mortem analyses in future work to further enhance statistical confidence and morphological understanding.

These revisions collectively ensure transparency, reproducibility, and robustness of our electrochemical conclusions.

Table S5. Cell parameters of the Li||NCM811 pouch cell

**Cell component**	**Details**
Cathode	NCM811 with Al collector<br>12 pieces of double-side<br>2.3 mAh cm^{-2} for each-side<br>12 μm for Al foil thickness
Separator	PE (25 μm)
Anode	free-standing 50 μm Li for double side
N/P ratio	2.2
E/C ratio	2.3 g Ah^{-1}

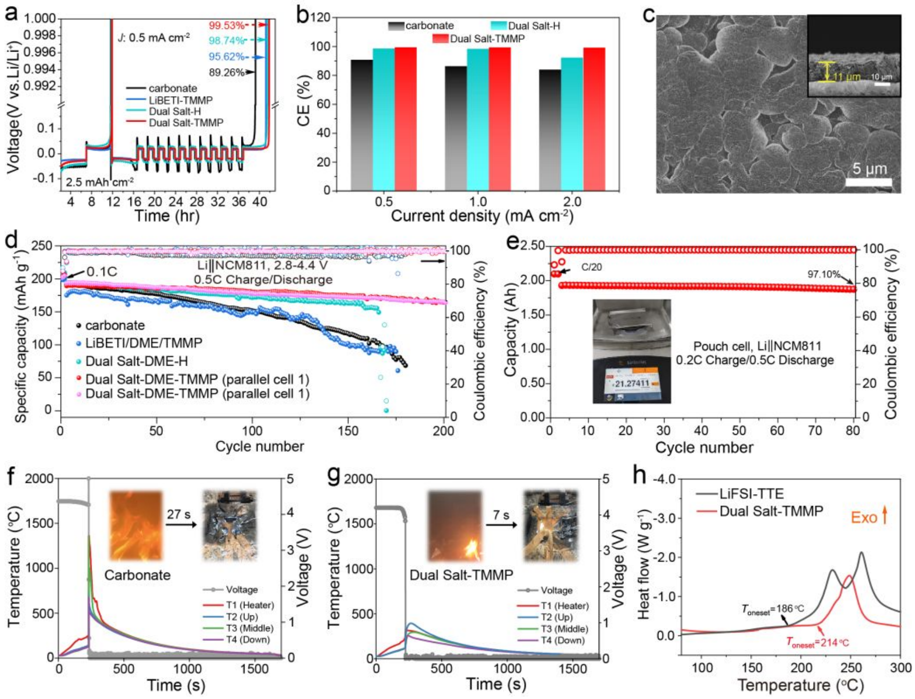

Figure 3. Electrochemical properties of different electrolytes. (a)-(b) CE tests of Li plating/stripping at current densities of 0.5, 1.0, and 2.0 mA cm^{-2} measured by the Aurbach method. (c) Top and cross-sectional SEM images of Li deposits on Cu in the Dual Salt-TMMP electrolyte (2 mAh cm^{-2}, 0.5 mA cm^{-2}). (d) The cyclability of Li||NCM811 batteries with a high cut-off voltage of 4.4 V at 30 °C. (e) Cycling performance of the Li||NCM811 pouch cell using Dual Salt-TMMP electrolyte (2.3 g Ah^{-1}). Temperature and voltage curves during thermal runaway for Li||NCM811 pouch cells using different electrolytes: (f) carbonate and (g) Dual Salt-TMMP electrolytes. (h) DSC thermograms for delithiated NCM811 with different electrolytes.

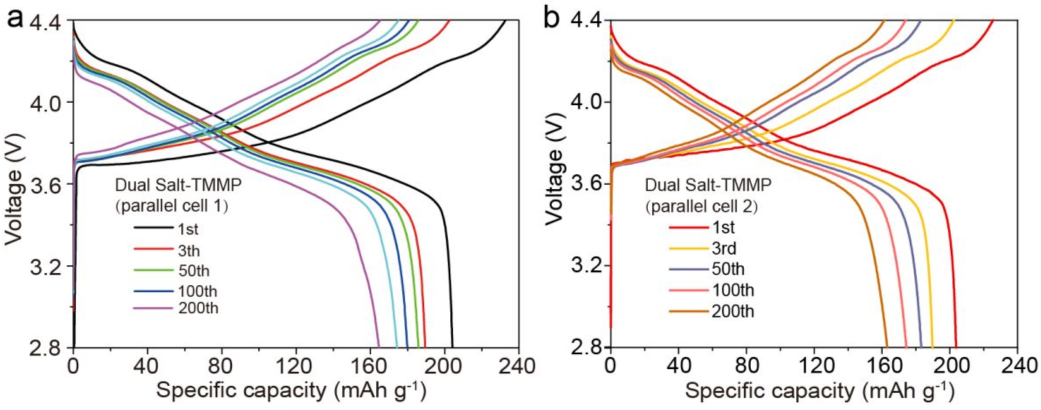

Figure S36. Voltage profiles of two parallel Li||NCM811 cells in the Dual Salt-TMMP electrolyte: (a) parallel cell 1, (b) parallel cell 2.

Changes in the manuscript:

A paragraph has been added on Pages 7 to elaborate on this point:

"Importantly, the Li||NCM811 battery with the Dual Salt-TMMP electrolyte demonstrates greatly improved cycling stability, exhibiting an average capacity retention of ~86% after 200 cycles, while the average CE reached 99.7% in both parallel experiments (Figures 3d and S36)."

4. High-voltage stability and Al corrosion

Voltammetry and cathode cycling suggest tolerance up to about 4.4-4.5 V. To make a broad high-voltage claim, include constant-potential leakage-current holds on inert electrodes with area normalization and statistics, and quantify Al dissolution by ICP after potential holds. Cyclic scans alone are not sufficient for a general stability statement.

Response: We thank the reviewer for the constructive suggestions. To address this comment, we have supplemented two sets of key experiments: constant-potential leakage-current measurements on inert electrodes and ICP-OES quantification of Al dissolution after potential holds, with details as follows:

We performed area-normalized (mA cm^{-2}) constant-potential holds on Al inert electrodes (2.83 cm^2) across voltage steps from 4.0 V to 4.6 V (vs. Li/Li^+). Each voltage step was maintained for 3600 s. As shown in the revised manuscript (**Figure S40**), the leakage current of the Dual Salt-TMMP electrolyte remains negligible up to 4.5 V, indicating excellent anodic stability. In comparison, the Dual Salt-H electrolyte exhibits slightly higher but still limited leakage current within the same voltage range. These results collectively confirm the superior high-voltage tolerance and anodic stability of the Dual Salt-TMMP electrolyte.

After the 4.6 V constant-potential hold, 0.60 mL of electrolyte was collected from each cell, diluted with deionized water, and adjusted to a final volume of 50.0 mL prior to ICP-OES analysis (corresponding to a dilution factor of ~83). The measured Al concentrations in the diluted samples were 0.000620 μg mL^{-1} for the Dual Salt-H electrolyte and 0.000263 μg mL^{-1} for Dual Salt-TMMP. After applying the dilution

correction, the Al concentrations in the original electrolytes were calculated to be 0.052 ppm and 0.022 ppm, respectively. These results clearly indicate that Al dissolution in both systems is extremely low, and that the Dual Salt-TMMP electrolyte exhibits roughly 2.6-fold lower Al corrosion than Dual Salt-H under identical high-voltage conditions, confirming its superior anodic stability.

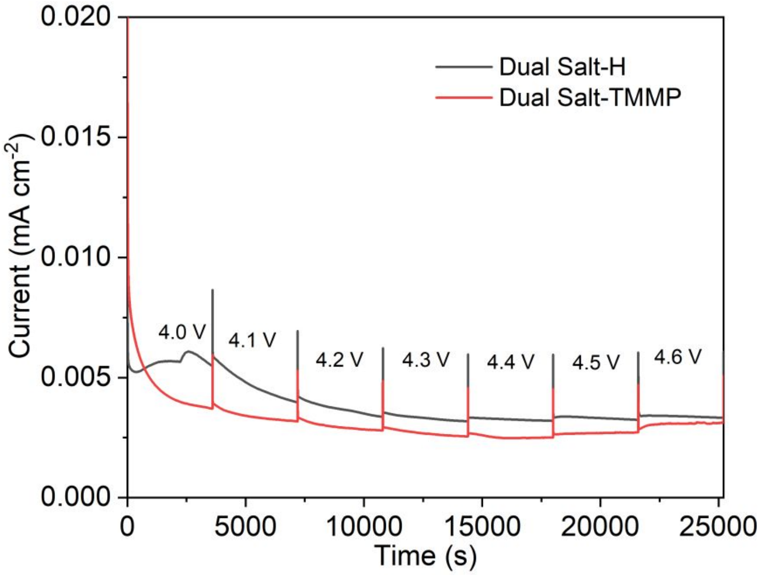

Figure S40. Constant-potential measurements of Li/Al cells in Dual Salt-H and Dual Salt-TMMP electrolytes. The potential was increased stepwise from 4.0 V to 4.6 V (vs. Li/Li^+), each step held for 3600 s, to assess the anodic stability of the electrolytes.

Changes in the manuscript:

A paragraph has been added on **Page 7** to elaborate on this point:

"Area-normalized constant-potential holds from 4.0 to 4.6 V (vs. Li/Li^+) revealed negligible leakage current for the Dual Salt-TMMP electrolyte, confirming its superior anodic stability compared with Dual Salt-H (Figure S40). ICP-OES analysis after the 4.6 V hold, with samples diluted 83-fold prior to measurement, showed minimal Al dissolution, with concentrations of 0.022 ppm for Dual Salt-TMMP (at the detection-limit level) and 0.052 ppm for Dual Salt-H. These results demonstrate that both electrolytes exhibit extremely low Al corrosion under high-voltage conditions, while Dual Salt-TMMP provides approximately 2.6-fold better suppression of anodic Al dissolution."

5. Flammability and thermal safety methodology

The closed-cup flash-point statement for TMMP and torch-flame tests show a clear safety advantage, but readers will expect standardized protocols and statistics. Please specify the standard used, sample mass and geometry, environment, and replicate counts for ignition and thermal-abuse tests. For the externally heated pouch cells, justify sensor placement and quantify onset and peak temperatures with uncertainties.

Response: We sincerely thank the reviewer for the valuable comments regarding the safety evaluation methodology. We fully agree that standardized testing details and statistical information are essential for reproducibility and fair comparison.

Accordingly, the Experimental Section has been revised to include the following details:

Flash point test: The flash point of TMMP was determined using a Pensky-Martens closed-cup method according to ASTM D6450, using 1 mL of sample in a micro-scale closed cup to minimize evaporation. Measurements were conducted at ambient pressure (~101 kPa) in air (25 ± 2 ^{o}C). Ignition was initiated via an electric arc according to the instrument protocol. Each measurement was repeated three times, and the average value with an uncertainty of ±1 °C is reported.

Torch-flame test: Conducted on 1.0 mL of electrolyte soaked into 19 mm diameter glass fiber discs, placed in open glass dishes (30 mm diameter) at 25 ± 2 °C under ambient pressure. Each test was performed in triplicate to ensure reproducibility.

Thermal-abuse test: A fully assembled pouch cell (2.3 Ah g^{-1}) was externally heated using a ceramic plate at a constant rate of 5 °C·min^{-1} under ambient conditions (~25 °C, 40–50 % RH). Temperature was monitored using K-type thermocouples: T1 was attached to the side adjacent to the heating plate, while T2, T3, and T4 were placed on the opposite side (upper edge, middle, lower edge) to capture spatial temperature variations. The actual thermocouple positions are shown in **Figure R2**, and the onset and peak temperatures are summarized in **Table S7**. This arrangement allows monitoring of both the heated surface and the opposing side, providing a comprehensive view of thermal progression. Thermocouples were fixed with high-temperature adhesive to prevent displacement during heating.

Due to the inherent complexity for preparation and safety considerations of pouch-

cell thermal-abuse testing, including the need for cell sealing, reinforced protective measures, and continuous temperature monitoring, each experiment was conducted once per electrolyte type. We acknowledge the limitations of this approach, and nevertheless, the measured onset and peak temperatures are consistent with the trends observed in the flammability and flash-point tests, providing strong evidence for the superior thermal safety of the TMMP-based electrolytes. We kindly ask for the understanding of reviewers regarding the practical constraints of these experiments.

Table S7. Onset and peak temperatures of pouch cells under external heating measured at different positions.

Pouch cells	Temperature (°C)	T1 (Heater)	T2 (Up)	T3 (Middle)	T4 (Down)
carbonate	Onset	28.3	28.2	26.2	28.2
	Peak	1360	747.3	1357	678.2
Dual Salt-TMMP	Onset	29.1	28.9	26	29
	Peak	334.6	393.9	295.8	256.8

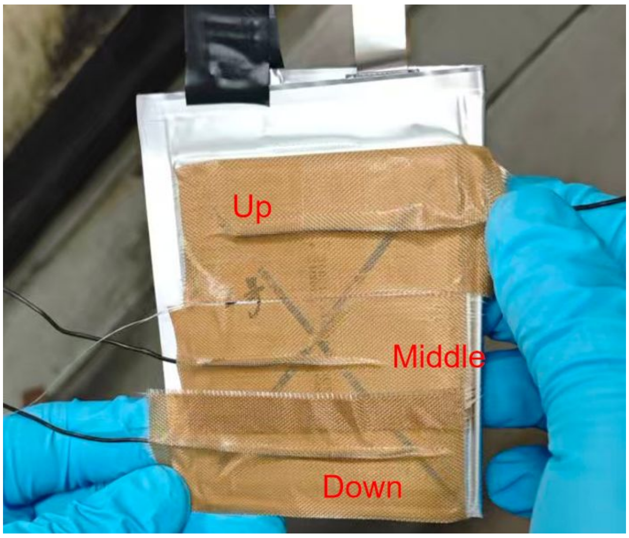

Figure R2. Schematic of thermocouple placement on a pouch cell during thermal-abuse testing.

Changes in the supporting information:

A paragraph has been added on Pages 4 to elaborate on this point:

"The flash point of TMMP was determined using a Pensky-Martens closed-cup method according to ASTM D6450, with 1 mL of sample placed in a micro-scale closed cup to

minimize evaporation. Measurements were carried out at ambient pressure (~101 kPa) in air at 25 ± 2 °C, and ignition was initiated via an electric arc following the instrument protocol. Each measurement was repeated three times. The torch-flame test was conducted on 1.0 mL of electrolyte, which was first soaked into 19 mm diameter glass fiber discs and then placed in open glass dishes (30 mm diameter) at 25 ± 2 °C under ambient pressure. Each test was performed in triplicate to ensure reproducibility. The thermal-abuse test was performed on a fully assembled pouch cell (2.3 Ah g^{-1}) subjected to external heating using a ceramic-plate heater at a constant rate of 5 °C·min^{-1} under ambient conditions (room air, ~25 °C, relative humidity ~40-50%). K-type thermocouples (TCs) were used to monitor temperature: one TC (T1) was attached to the side of the pouch cell directly adjacent to the heating plate, while three additional TCs (T2, T3, T4) were placed on the opposite side at distinct locations (center, upper edge, lower edge) to capture spatial temperature gradients. The sensor placement was chosen to monitor both the direct heating interface and the opposing surface, providing a comprehensive picture of thermal progression. Thermocouples were affixed with high-temperature adhesive and secured to prevent displacement during heating."

6. DFT HOMO/LUMO language

Where molecular orbital energies are listed and then qualitatively connected to reduction or oxidation tendencies, the analysis is not physically rigorous. Kohn–Sham HOMO/LUMO values are not redox potentials. For anions in particular, the "LUMO" has no direct meaning for the real condensed-phase electron uptake process unless an extra electron is explicitly included and stabilized by a proper solvation model. If the goal is to discuss reductive decomposability, compute solution-phase reduction free energies for explicit first-shell clusters or refrain from making such connections. This is important because widespread casual use of HOMO/LUMO for electrochemical stability has led to misleading interpretations in the electrolyte literature.

Response: We sincerely appreciate the reviewer's insightful comment regarding the use of HOMO/LUMO analysis in predicting electrochemical stability. We fully agree that Kohn–Sham orbital energies are not rigorous indicators of redox potentials, and we

recognize that their overuse in the electrolyte literature has sometimes led to misleading interpretations. However, for the preliminary screening of molecular components, HOMO/LUMO analysis remains a useful qualitative tool to identify relative oxidative or reductive tendencies. This approach helps narrow down promising candidates before performing more sophisticated solvation and redox free energy calculations.

In addition to the HOMO/LUMO discussion, our work provides multiple layers of experimental and theoretical validation to support the electrolyte stability trends. Specifically, we have conducted electrochemical tests (such as LSV and cycling performance), surface characterizations (XPS and SEM), and impedance analyses (EIS and DRT). These comprehensive data consistently corroborate the DFT-predicted stability order, indicating that the HOMO/LUMO-derived trends are in line with experimental observations.

Moreover, the reviewer's suggestion to compute the redox stability of solvated clusters was also a central focus of our study. Accordingly, we have calculated the oxidation and reduction free energies of explicit solvation clusters to assess their solution-phase thermodynamic stability. The results (**Figure S52**) further confirm that the dual-salt system, particularly in the TMMP-based solvent, exhibits enhanced oxidative and reductive robustness. These cluster-level calculations provide a more physically meaningful picture of electrolyte stability, complementing the qualitative orbital analysis.

Changes in the manuscript:

A paragraph has been added on **Pages 9** to elaborate on this point:

✧ "In addition, the XPS spectra of S 2p and N 1s (Figures S50-S51) confirm the presence of inorganic components such as SO_x, N-SO_x, Li_3N, and Li_2S in both the Dual Salt-H and Dual Salt-TMMP systems, further contributing to the stability of the SEI. DFT calculations were performed to qualitatively examine the electronic characteristics of the electrolyte components. As shown in Figure S52, TMMP exhibits a relatively high reduction potential compared with FSI^- and $BETI^-$, suggesting its intrinsically higher susceptibility to reduction. However, due to the

absence of TMMP in the inner solvation structure, its contribution to SEI formation is minimal. In contrast, when FSI^- and $BETI^-$ are coordinated with Li^+, their reduction potentials decrease markedly. These results qualitatively support that the observed inorganic species mainly originate from anion-derived decomposition."

✧ "In addition, TMMP can also participate in LiF generation through oxidative decomposition at high voltages, as its TMMP-anion complex exhibits a lower-oxidation potential than the isolated TMMP molecule (Figure S52). In contrast, the CEI formed in the carbonate and Dual Salt-H electrolytes (Figures S54-S55) contains a higher fraction of organic species such as $ROCO_2Li$ and C-O components, along with weaker LiF and N-SO_x signals, indicating a less stable interphase."

7. Internal consistency and unit hygiene

Viscosity, density, conductivity, and CE figures appear in multiple places with slightly different values or formats. Please consolidate into one summary table with units, significant figures, and n for each formulation. Ensure that all comparisons are at the same temperature.

Response: We thank the reviewer for this valuable comment. We have carefully re-examined all reported data for viscosity, density, ionic conductivity, and CE.

The viscosity and density data are provided in **Table S4**, where each value represents the average of three independent measurements. To ensure consistency and clarity, all viscosity and density values are now reported with two decimal places. Similarly, the ionic conductivity data are summarized in **Table S3**, with each value corresponding to the average of three measurements and also reported with two decimal places. In addition, several CE values previously exhibited inconsistent decimal formatting, which has now been standardized to two decimal places throughout the manuscript.

We would like to emphasize that these datasets were not merged into a single table, as each physical property reflects distinct scientific aspects and was measured for different solvent or electrolyte systems. Combining them into one table would obscure the contextual meaning of the data and introduce unnecessary redundancy. Presenting

them separately ensures clarity, readability, and scientific precision.

All measurements were conducted at 25 °C, and all values have been carefully verified for internal consistency, proper units, and significant figures.

Table S3. The physical properties of the studied electrolytes (25 °C)

Electrolytes	Ionic conductivity (mS cm^{-1})	Ion mobility number (t_{Li})	Diffusion coefficient (D_{Li}) (m^2/s)
Dual Salt-H	1.38 ± 0.03	0.53	1.36×10^{-11}
Dual Salt-TMMP	7.18 ± 0.08	0.56	6.98×10^{-11}

Note: The ionic conductivity (σ) of each electrolyte was measured three times independently at a fixed temperature of 25 °C using a calibrated conductivity meter. The reported values are presented as mean ± standard deviation (mean ± SD) to reflect experimental reproducibility.

Table S4. Viscosity data for the studied solvents and electrolytes at 25°C (All values represent the average of three independent measurements)

Solvents/Electrolytes	Viscosity (mPa·s)	Density (g/cm^3)
TTE	1.43 ± 0.02	1.54 ± 0.01
TMMP	1.38 ± 0.01	1.67 ± 0.02
LiFSI-DME-TTE (1.25:2:2 by molar ratio)	7.18 ± 0.04	1.32 ± 0.02
LiFSI-LiBETI-DME-TTE (0.25:1:2:2 by molar ratio)	11.85 ± 0.03	1.30 ± 0.01
Dual Salt-TMMP	12.65 ± 0.02	1.31 ± 0.01

8. Provide the equivalent circuits, fitting bounds, and residuals for every EIS plot used to extract R_{bulk} and interfacial resistances.

Response: We sincerely appreciate the reviewer's valuable suggestion regarding the inclusion of more detailed information on EIS analysis. Although the original manuscript did not discuss EIS results, in response to this comment, we have conducted additional EIS measurements for Li||Cu, Li||Li, and Li||NCM811 cells using three representative electrolytes: carbonate, Dual Salt-H, and Dual Salt-TMMP.

In the Nyquist plots, the mid- to low-frequency semicircle corresponds to the combined contributions of SEI/CEI and charge-transfer, whereas the inclined line in the low-frequency region is associated with Li^+ diffusion. The corresponding equivalent circuit used for fitting is provided in Figure S23. However, because the SEI, CEI, and charge-transfer processes are strongly convoluted, the fitting results of equivalent-circuit models are highly sensitive to the selected frequency range. Such subjectivity may lead to ambiguous interpretations of the underlying physical processes. To address this limitation, we employed the distribution of relaxation times (DRT) method to re-analyze the impedance spectra. Unlike equivalent-circuit fitting, DRT does not require any predefined model and instead uses mathematical deconvolution to resolve the individual electrochemical processes operating at different time scales. This approach provides a more objective and reliable basis for understanding interfacial evolution in the studied electrolytes.

For the Li||Cu cells (**Figures S22**), the impedance of carbonate electrolyte increases rapidly upon cycling, indicating unstable SEI growth and continuous interfacial reactions. In contrast, both the Dual Salt-H and Dual Salt-TMMP electrolytes show a clear impedance reduction over the tested cycles, suggesting that after initial SEI formation, the interphase undergoes structural rearrangement that enhances interfacial kinetics. Remarkably, the Dual Salt-TMMP delivers the smallest charge-transfer resistance and most stable DRT features after 20 cycles, demonstrating the formation of a dense and ionically conductive SEI that effectively stabilizes the Li surface. These results align with the significantly improved Li plating/stripping reversibility observed in Li||Cu cells.

Similar trends are observed in Li||Li symmetric cells (**Figure S25**) and Li||NCM811 full cells (**Figure S37**). While the carbonate electrolyte maintains high interfacial impedance throughout cycling, both Dual Salt-H and Dual Salt-TMMP exhibit a pronounced impedance decrease. Among them, Dual Salt-TMMP consistently achieves the lowest overall impedance and most stable DRT response, demonstrating superior Li^+ transport kinetics and interfacial stability at both electrodes.

Collectively, these EIS results confirm that the Dual Salt-TMMP electrolyte facilitates the most stable SEI and CEI across all cell configurations, enabling highly reversible Li^+ transport and sustained interfacial stability.

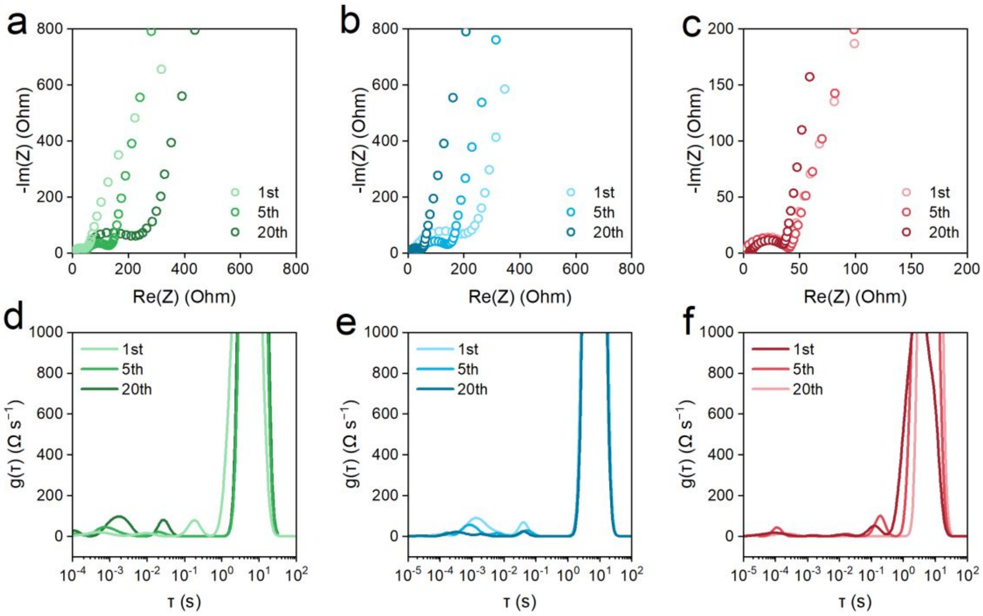

Figure S22. EIS and corresponding DRT analysis of Li||Cu cells using different electrolytes: (a, d) carbonate, (b, e) Dual Salt-H, and (c, f) Dual Salt-TMMP.

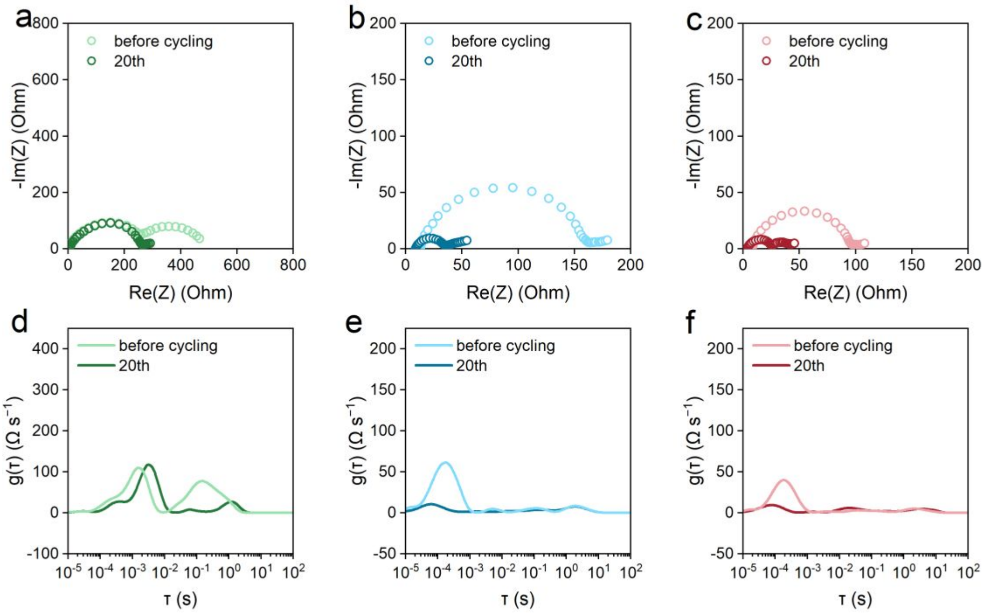

Figure S25. EIS and corresponding DRT analysis of Li||Li cells using different electrolytes: (a, d) carbonate, (b, e) Dual Salt-H, and (c, f) Dual Salt-TMMP.

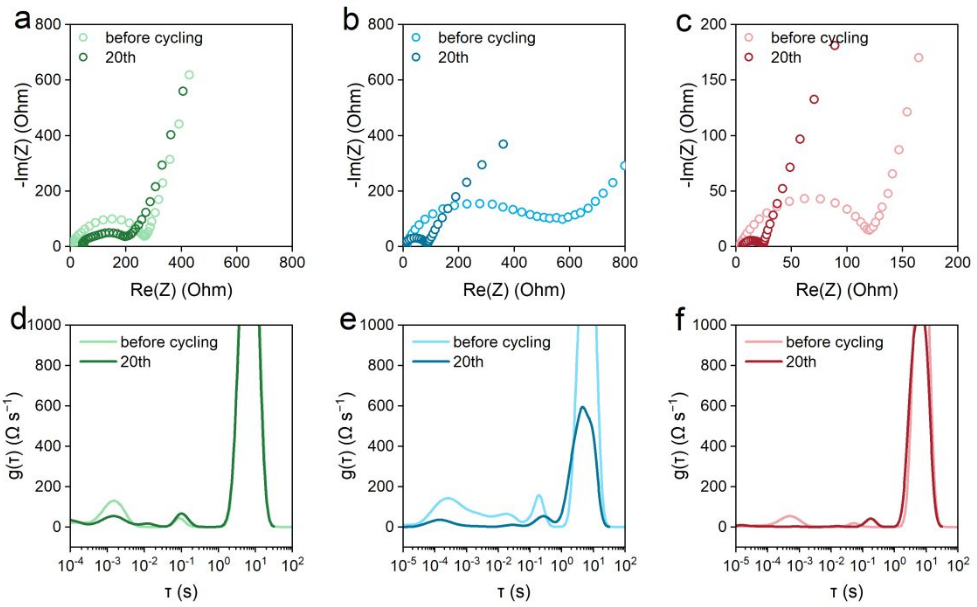

Figure S37. EIS and corresponding DRT analysis of NCM811||Li cells using different electrolytes: (a, d) carbonate, (b, e) Dual Salt-H, and (c, f) Dual Salt-TMMP.

Changes in the manuscript:

A paragraph has been added on Pages 6 to elaborate on this point:

- "Consistently, electrochemical impedance spectroscopy (EIS) measurements (Figures S22-S23) show that, compared with the carbonate and Dual Salt-H electrolytes, the Dual Salt-TMMP system exhibits minimal impedance growth and highly stable distribution of relaxation time (DRT) features, further evidencing enhanced interfacial stability and reversible Li deposition."
- "EIS measurements further confirm this trend (Figure S23 and S25), with the Dual Salt-TMMP electrolyte exhibiting the lowest and most stable interfacial resistance and consistent DRT features over cycling, highlighting its superior ability to maintain a highly conductive and stable SEI compared to the other electrolytes."

A paragraph has been added on Pages 7 to elaborate on this point:

- "As shown in Figures S23 and S37, the EIS results indicate that the Dual Salt-TMMP electrolyte effectively suppresses the growth of interfacial resistance over 25 cycles, whereas the carbonate system exhibits a pronounced increase in polarization. Corresponding DRT analysis further confirms that Dual Salt-TMMP

minimizes both SEI and charge-transfer related relaxation processes, highlighting improved Li^+ transport and enhanced interfacial stability at both electrodes."

9. Report water content of each electrolyte mixture and the handling steps, since trace water strongly affects LiFSI chemistry.

Response: We thank the reviewer for this important comment. Both LiFSI and LiBETI salts were dried in a vacuum oven inside an Ar-filled glovebox at 100 °C for 72 h prior to use. All solvents (chromatography grade, anhydrous, ≥99.9%) were thoroughly dried over 4 Å molecular sieves for at least 72 h before use to remove trace moisture. The water content of each prepared electrolyte mixture was measured by Karl-Fischer titration and found to be below 20 ppm. All electrolyte preparation, storage, and handling were conducted entirely in the glovebox with H_2O and O_2 levels below 0.1 ppm, minimizing moisture uptake. These procedures ensure the chemical stability of the salts and the reproducibility of the electrochemical data.

Changes in the supporting information:

A paragraph has been added on **Pages 2** to elaborate on this point:

"**Materials**: Li foils (Φ = 15.6 mm, thickness = 450 μm) were purchased from China Energy Lithium Co. Ltd. The ethylene carbonate (EC), diethyl Carbonate (DEC), methyl ethyl carbonate (EMC), and vinylene carbonate (VC) were purchased from Sigma-Aldrich. The TMMP was purchased from Aladdin Co. Ltd. All solvents (chromatography grade, anhydrous, ≥99.9%) were thoroughly dried over 4 Å molecular sieves for at least 72 h before use to remove trace moisture. Battery-grade $LiPF_6$ and LiFSI were purchased from DodoChem Co. Ltd. The LiBETI was purchased from the Tokyo Chemical Industry. Both LiFSI and LiBETI salts were dried in a vacuum oven inside an Ar-filled glovebox at 100 °C for 72 h prior to use. The 2 Ah-class NCM811||Li pouch cell, a dry cell supplied by EVE Battery Ltd., was used in the experiment."

10. Clarify whether any sample rinsing precedes XPS and how potential reconstruction artifacts were mitigated.

Response: We thank the reviewer for the comment. Prior to XPS analysis, the electrodes were gently rinsed with anhydrous DME inside an Ar-filled glovebox to remove residual electrolyte. The samples were then transferred directly to the XPS instrument using an air-free transfer device, avoiding exposure to air or moisture. These procedures minimize potential reconstruction artifacts and ensure that the acquired spectra accurately reflect the original electrode surface. Furthermore, mild sputtering conditions were employed during XPS depth profiling to preserve the original chemical state of the samples and ensure reliable XPS measurements. Relevant experimental details have been added to the "**Characterization**" section of the manuscript to provide clarity and reproducibility.

Changes in the Supporting Information:

A paragraph has been added on **Pages 3** to elaborate on this point:

"The cycled Li metal and cathodes were analyzed using a Physical Electronics Quantera scanning X-ray microprobe with a focused monochromatic Al $K\alpha$ X-ray source. Prior to XPS measurement, the electrodes were gently rinsed several times with anhydrous DME inside an Ar-filled glovebox to remove residual electrolyte. The samples were then transferred to the XPS instrument using a self-designed hermetically sealed container filled with argon gas, preventing exposure to ambient oxygen and moisture."

11. Quote separator type and thickness, stack pressure, and electrolyte volume for every electrochemical test figure.

Response: We thank the reviewer for the valuable comment. The "**Electrochemical Measurements**" section of the Supporting Information originally provides detailed specifications for the separator type and thickness, stack pressure, and electrolyte volume used in all electrochemical tests. Based on your suggestion, we have further supplemented these details to ensure that all electrochemical test figures are fully documented and easily reproducible. The results regarding the PE separator used in the pouch cells can be found in **Table S5** of the Supporting Information.

Changes in the Supporting Information:

A paragraph has been added on **Pages 5** to elaborate on this point:

1. "LSV, Tafel plots, and CV tests were carried out on BioLogic VMP-3. The electrochemical tests of Li||Cu, Li||Li, Li||NCM811, and Li||LCO were conducted using LANHE battery testers at 30 °C in environmental chambers, with Li foil as the anode. During the assembly of Li||Li cells, a spring clip was used, along with a 0.5 mm spacer, two 450 μm Li foils, and a Celgard 2500 separator with a thickness of 25 μm."

2. "The oil press of all the batteries was set to 850 psi, with no additional pressure applied."

2. "75 μL of electrolyte was added to each cell mentioned above."

Table S5. Cell parameters of the Li||NCM811 pouch cell

**Cell component**	**Details**
Cathode	NCM811 with Al collector<br>12 pieces of double-side<br>2.3 mAh cm^{-2} for each-side<br>12 μm for Al foil thickness
Separator	PE (25 μm)
Anode	free-standing 50 μm Li for double side
N/P ratio	2.2
E/C ratio	2.3 g Ah^{-1}

Reviewer #4:

In current submission, Chen and co-workers focus on the miscibility issues of the fluorinated electrolytes, reporting the unique impact brought by a fluorinated anions containing bulky fluorinated groups. The experimental design is technically sound and the results are of critical importance for battery community. The conclusions are very convincing and the manuscript is well constructed. I would recommend the publication of this work after addressing minor issues as below.

Response: We sincerely thank the reviewer for the very positive and encouraging evaluation of our work. We highly appreciate the reviewer's constructive comments and valuable suggestions, which have helped us further improve the clarity and completeness of the manuscript. In the revised version, we have carefully addressed all the comments raised by the reviewer point by point, with corresponding modifications highlighted in the revised manuscript. We believe that these revisions have further strengthened the scientific quality and readability of our work. The detailed responses to each specific comment are provided below.

1. The anion BETI has been well studied by Passerini et al. (cf. Journal of The Electrochemical Society 149, A891-A897, doi:10.1149/1.1483098 (2002); Journal of The Electrochemical Society 149, A1282-A1285, doi:10.1149/1.1502688 (2002).), the authors are recommended to provide sufficient background for the use of BETI anion in this work.

Response: We thank the reviewer for this valuable suggestion. We have cited the pioneering works of Passerini et al. and emphasized the rationale behind our electrolyte system design in the revised introduction. In our work, the design of the LiFSI-LiBETI dual-salt electrolyte with TMMP as a diluent was motivated by a key challenge: at high salt concentrations, low-polarity TMMP tends to undergo phase separation, which severely limits salt solubility and compromises electrolyte performance.

To overcome this, LiFSI was retained as the primary salt to ensure high ionic conductivity and stable electrode compatibility, while LiBETI was introduced as a dual-salt to enhance intermolecular interactions among anion, solvent, and diluent. This

dual-salt strategy effectively suppresses phase separation, enabling the incorporation of flame-retardant TMMP without sacrificing solubility or battery performance. Overall, our system design highlights a careful balance between safety, electrochemical stability, and molecular compatibility, demonstrating both the innovation and practical significance of this work.

Changes in the manuscript:

A paragraph has been added on **Pages 2** to elaborate on this point:

"To address this critical issue, we delve into the immiscibility issue of fluorous diluents and propose a novel amphiphilic anion chemistry to bridge the solvent and fluorous diluents. Fluorinated anions have long been recognized for their ability to stabilize interfaces and improve ionic transport. In particular, Passerini and co-workers demonstrated that the $BETI^-$ anion [bis(perfluoroethylsulfonyl)imide, $N(SO_2CF_2CF_3)_2^-$] provides excellent interfacial stability with lithium metal and high ionic conductivity in polymer electrolytes.[34, 35] By introducing anions with long fluoro-alkyl moieties (e.g., $BETI^-$), we induce favorable fluorophilic (F···F) interactions with fluorous diluents (e.g., 2-trifluoromethyl-3-methoxyperfluoropentane, TMMP, F/H=4.33; 1H-perfluorohexane, F/H=13) and atypical hydrogen-bonding (F···H) interactions with the solvent (DME), successfully resolving the immiscibility issue."

2. The NMR spectra of the LiBETI salt will be necessary to confirm its chemical structure and possible contamination of impurities.

Response: To address your concern about confirming the chemical structure of the LiBETI salt and identifying potential impurity contamination, we have supplemented the ^{19}F NMR experiment (solvent: CD_3CN, frequency: 377 MHz) and analyzed the spectrum in detail, with the key results as follows:

The molecular structure of LiBETI contains two equivalent perfluoroethylsulfonyl (-$SO_2C_2F_5$) groups. The ^{19}F NMR spectrum of the target sample shows two distinct singlet peaks (s) at chemical shifts (δ) of -79.90 ppm and -118.29 ppm, which are fully consistent with the characteristic fluorine signals of LiBETI: The peak at -79.90 ppm

corresponds to the fluorine atoms on the terminal -CF_3 groups of the perfluoroethyl chains. The peak at -118.29 ppm is attributed to the fluorine atoms on the internal -CF_2- groups adjacent to the sulfonyl group (-$SO_2CF_2CF_3$).

The singlet splitting pattern (no multiplet splitting) and the relative intensity ratio of the two peaks (consistent with the stoichiometric ratio of fluorine atoms in two -$SO_2C_2F_5$ groups of LiBETI) further confirm that the main component of the sample is the target LiBETI salt, with no structural deviations. The LiBETI salt used in this study was commercially purchased, so it was not included in the article.

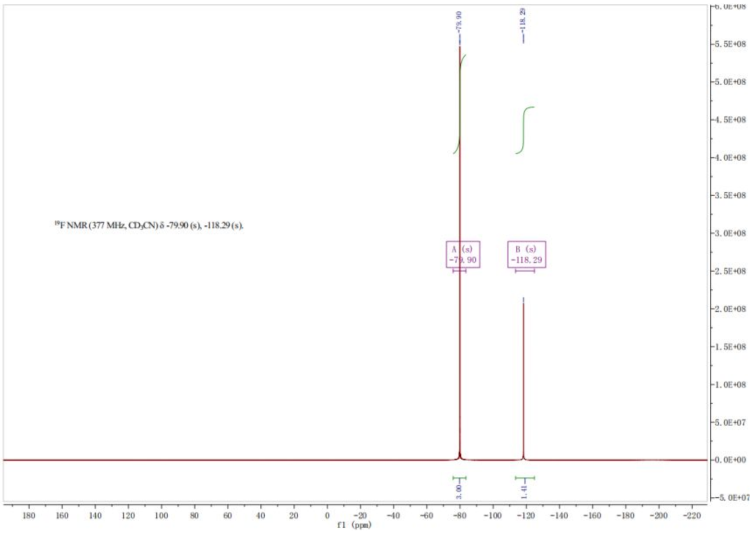

Figure R2. ^{19}F NMR spectrum of LiBETI.

3. The authors may expand the discussion on future design of new anions and how this could be aligned with solvent optimization.

Response: We thank the reviewer for this valuable suggestion. We have expanded the discussion in the revised manuscript to include future perspectives on rational design of new anions and their alignment with solvent optimization. Specifically, we have added a paragraph in the discussion section highlighting how the structural tuning of anions (e.g., introducing amphiphilic or partially fluorinated motifs) can be synergistically coupled with solvent selection to balance solvation strength, miscibility, and interfacial stability. This addition provides guidance for future electrolyte development strategies.

Changes in the manuscript:

A paragraph has been added on **Pages 10** to elaborate on this point:

"In conclusion, this study presents a novel approach to addressing the safety and performance challenges in high-energy-density lithium metal batteries through the development of miscible fluorous electrolytes enabled by amphiphilic anion chemistry. By employing anions with fluoro-alkyl moieties, specifically $BETI^-$, we successfully bridged the gap between Li^+-solvating solvents and highly fluorinated diluents, resolving the critical issue of immiscibility in fluorous electrolytes. The Dual Salt-TMMP electrolyte exhibits remarkable lithium metal reversibility, achieving a high CE of 99.5%, and enables stable cycling of Li||NCM811 cells with 87% capacity retention after 200 cycles at 4.4 V, facilitated by LiF-rich electrode-electrolyte interphases. It also shows enhanced thermal stability and flame retardancy, addressing key safety concerns. This amphiphilic anion approach provides new opportunities for tailoring electrolyte compositions to meet the demands of next-generation energy storage systems. Looking ahead, rational anion design can further improve miscibility and electrochemical performance. Beyond fully fluorinated anions like $BETI^-$, amphiphilic or partially fluorinated motifs can balance polarity and fluorophilicity, regulate Li^+ coordination, and enhance compatibility with solvents such as TMMP. Although highly fluorinated components are beneficial for electrochemical stability and battery safety, they are often associated with high cost and significant environmental concerns. Therefore, future efforts should focus on developing green recycling strategies, designing degradable fluorinated species, or exploring fluorine-free molecular alternatives to achieve sustainable electrolyte systems for safe and high-energy batteries."

oc-2025-01711t.R2

Name: Peer Review Information for "Molecularly Engineered Amphiphilic Anions Enable Flame-Retarding Fluorous Electrolytes for Lithium Metal Batteries"

Second Round of Reviewer Comments

Reviewer: 3

Comments to the Author

Authors have mostly addressed my points, anyhow, there is still lack or rigorous statistical treatment.

Specifically, conductivity now has n = 3 with mean ± SD at 25 °C and tLi^+ is cross-validated by Bruce–Vincent, consistent with PFG-NMR values (Table S3; Figure R1; response pages ~33–35). But no uncertainty propagation to derived quantities such as $\sigma \cdot tLi^+$

Reviewer: 1

Comments to the Author

The authors have substantially improved the manuscript in this revision, and I now recommend acceptance. The central concept—using an amphiphilic fluoroalkyl anion to bridge the ether solvent and fluorinated diluent, thereby resolving immiscibility while enabling high performance and enhanced safety—is clearly articulated and supported by a comprehensive set of ITC, multinuclear NMR (including HOESY), ESI-MS, DFT/AIMD, and transport measurements that together provide convincing mechanistic evidence for the proposed “molecular bridge” behavior. In addition, the expanded electrochemical data (Li|Cu CE, Li|Li cycling, high-voltage Li|NCM811 coin cells, and pouch-cell demonstrations) and the flammability/thermal-runaway tests convincingly demonstrate the practical relevance of the electrolyte design. Overall, the concerns regarding mechanism, generality, and cell-level performance have been appropriately addressed, only minor wording/typographical issues remain that can be handled during production, and the work

now represents a timely and significant contribution suitable for publication in its current form.

Author's Response to Peer Review Comments:

Dear Editor,

Thanks very much for the great efforts from you and the reviewer for improving our manuscript. We have carefully addressed the question raised by the reviewer and provided a detailed response and revision change in our response letter attached.

Please let us know if anything else is need.

Thanks again,

Xiaodi Ren

Responses to reviewers' suggestions and comments for manuscript No. oc-2025-01711t.R1

We would like to thank the reviewers for their valuable comments. We have incorporated most of the reviewers' comments and suggestions into the revised manuscript. The changes to the supporting information are marked yellow in this response and in the revised supporting information.

Reviewer #3:

Authors have mostly addressed my points, anyhow, there is still lack or rigorous statistical treatment. Specifically, conductivity now has n = 3 with mean ± SD at 25 °C and t_{Li+} is cross-validated by Bruce-Vincent, consistent with PFG-NMR values (Table S3; Figure R1; response pages ~33-35). But no uncertainty propagation to derived quantities such as $\sigma \cdot t_{Li+}$.

Response: We sincerely thank the reviewer for highlighting the need for more rigorous statistical treatment. Following your suggestion, we have strengthened the quantitative analysis of Li-ion transport parameters in the revised supporting information. Ionic conductivity (σ) has been reported as mean ± SD (n = 3) at 25 °C (**Table S3**). The Li^+ transference number ($\boldsymbol{t_{Li^+}}$) was independently validated using both the Bruce-Vincent method and PFG-NMR. To further improve statistical reliability, three independent Bruce-Vincent measurements were performed for each sample, and the resulting averaged values with standard deviations are 0.52 ± 0.02 for Dual Salt-H and 0.60 ± 0.05 for Dual Salt-TMMP. The propagated uncertainty for the derived parameter $\boldsymbol{\sigma \cdot t_{Li^+}}$ has also been calculated and incorporated into the revised supporting information using standard error-propagation:

$$\boldsymbol{X = \sigma \cdot t_{Li^+}, \Delta X = X\sqrt{\left(\frac{\Delta\sigma}{\sigma}\right)^2 + \left(\frac{\Delta t_{Li^+}}{t_{Li^+}}\right)^2}}$$

Notably, the propagated uncertainties of $\boldsymbol{\sigma \cdot t_{Li^+}}$ (Li^+) are relatively small (e.g., only 0.03

for Dual Salt-H), this directly confirms the accuracy and stability of the derived values in reflecting electrolyte ionic transport properties. The results align with the cross-validation of Bruce-Vincent/PFG-NMR methods, fully proving the reliability/reproducibility of Li-ion transport parameters. This rigorous treatment further solidifies the validity of our conclusions on Li^+ mobility and electrolyte performance.

Table S3. The physical properties of the studied electrolytes (25 °C)

Electrolytes	Ionic conductivity (σ) (mS cm^{-1})	Ion mobility number (t_{Li^+}) (PFG-NMR)	Diffusion coefficient (D_{Li^+}) (m^2/s)	Ion mobility number (t_{Li^+}) (Bruce-Vincent)	$\sigma \cdot t_{Li^+}$ (mean ± propagated SD, mS·cm^{-1})
Dual Salt-H	1.38 ± 0.03	0.53	1.36×10^{-11}	0.52 ± 0.02	0.72 ± 0.03
Dual Salt-TMMP	7.18 ± 0.08	0.56	6.98×10^{-11}	0.60 ± 0.05	4.31 ± 0.36

Changes in the Supporting Information:

A paragraph has been added on Page 10 to elaborate on this point:

"**Note：** The ionic conductivity (σ) of each electrolyte was measured three times independently at a fixed temperature of 25 °C using a calibrated conductivity meter. The reported values are presented as mean ± standard deviation (mean ± SD) to reflect experimental reproducibility. The propagated uncertainty for the derived parameter $\sigma \cdot t_{Li^+}$ (Li^+) has also been calculated and incorporated into the Table S3 using standard error-propagation:

$$\boldsymbol{X = \sigma \cdot t_{Li^+}, \Delta X = X\sqrt{\left(\frac{\Delta\sigma}{\sigma}\right)^2 + \left(\frac{\Delta t_{Li^+}}{t_{Li^+}}\right)^2}}$$

The resulting propagated uncertainties of $\sigma \cdot t_{Li^+}$ (Li^+) are relatively small (e.g., 0.03 for Dual Salt-H), confirming the accuracy and stability of the derived values in reflecting

electrolyte ionic transport properties. These results are consistent with the cross-validation between Bruce-Vincent and PFG-NMR measurements, demonstrating the reliability and reproducibility of the Li-ion transport parameters. This rigorous statistical treatment further reinforces the validity of our conclusions regarding Li^+ mobility and overall electrolyte performance.
